# Differently increased volumes of multiple brain areas in *Npc1* mutant mice following various drug treatments

**DOI:** 10.3389/fnana.2024.1430790

**Published:** 2024-07-16

**Authors:** Veronica Antipova, Diana Heimes, Katharina Seidel, Jennifer Schulz, Oliver Schmitt, Carsten Holzmann, Arndt Rolfs, Hans-Jürgen Bidmon, Estibaliz González de San Román Martín, Pitter F. Huesgen, Katrin Amunts, Jonas Keiler, Niels Hammer, Martin Witt, Andreas Wree

**Affiliations:** ^1^Institute of Anatomy, Rostock University Medical Center, Rostock, Germany; ^2^Division of Macroscopic and Clinical Anatomy, Gottfried Schatz Research Center for Cell Signaling, Metabolism and Aging, Medical University of Graz, Graz, Austria; ^3^Department of Oral and Maxillofacial Surgery, University Medical Center Mainz, Mainz, Germany; ^4^Klinik für Frauenheilkunde und Geburtshilfe, Dietrich-Bonhoeffer-Klinikum, Neubrandenburg, Germany; ^5^Department of Anatomy, Medical School Hamburg, University of Applied Sciences and Medical University, Hamburg, Germany; ^6^Institute of Medical Genetics, Rostock University Medical Center, Rostock, Germany; ^7^Centre of Transdisciplinary Neuroscience Rostock, Rostock, Germany; ^8^Medical Faculty, University of Rostock, Rostock, Germany; ^9^Institute of Neurosciences and Medicine, Structural and Functional Organisation of the Brain (INM-1), Forschungszentrum Jülich, Jülich, Germany; ^10^Central Institute of Engineering, Electronics and Analytics, ZEA-3, Forschungszentrum Jülich, Jülich, Germany; ^11^Centro Joxe Mari Korta, Donostia, Spain; ^12^Institut für Biologie II, AG Funktional Proteomics, Freiburg, Germany; ^13^C. and O. Vogt Institute for Brain Research, University Hospital Düsseldorf, University Düsseldorf, Düsseldorf, Germany; ^14^Department of Orthopedic and Trauma Surgery, University of Leipzig, Leipzig, Germany; ^15^Division of Biomechatronics, Fraunhofer Institute for Machine Tools and Forming Technology, Dresden, Germany; ^16^Department of Anatomy, Technische Universität Dresden, Dresden, Germany; ^17^Department of Anatomy, Institute of Biostructural Basics of Medical Sciences, Poznan Medical University, Poznan, Poland

**Keywords:** NPC1, mouse, lipid storage disorder, treatment, miglustat, 2-hydroxypropyl-β-cyclodextrin, fresh volumes, brain areas

## Abstract

**Background:**

Niemann-Pick disease type C1 (NPC1, MIM 257220) is a heritable lysosomal storage disease characterized by a progressive neurological degeneration that causes disability and premature death. A murine model of *Npc1^−/−^* displays a rapidly progressing form of Npc1 disease, which is characterized by weight loss, ataxia, and increased cholesterol storage. *Npc1^−/−^* mice receiving a combined therapy (COMBI) of miglustat (MIGLU), the neurosteroid allopregnanolone (ALLO) and the cyclic oligosaccharide 2-hydroxypropyl-β-cyclodextrin (HPßCD) showed prevention of Purkinje cell loss, improved motor function and reduced intracellular lipid storage. Although therapy of *Npc1^−/−^* mice with COMBI, MIGLU or HPßCD resulted in the prevention of body weight loss, reduced total brain weight was not positively influenced.

**Methods:**

In order to evaluate alterations of different brain areas caused by pharmacotherapy, fresh volumes (volumes calculated from the volumes determined from paraffin embedded brain slices) of various brain structures in sham- and drug-treated wild type and mutant mice were measured using stereological methods.

**Results:**

In the wild type mice, the volumes of investigated brain areas were not significantly altered by either therapy. Compared with the respective wild types, fresh volumes of specific brain areas, which were significantly reduced in sham-treated *Npc1^−/−^* mice, partly increased after the pharmacotherapies in all treatment strategies; most pronounced differences were found in the CA1 area of the hippocampus and in olfactory structures.

**Discussion:**

Volumes of brain areas of *Npc1^−/−^* mice were not specifically changed in terms of functionality after administering COMBI, MIGLU, or HPßCD. Measurements of fresh volumes of brain areas in *Npc1^−/−^* mice could monitor region-specific changes and response to drug treatment that correlated, in part, with behavioral improvements in this mouse model.

## 1 Introduction

Niemann–Pick type C (NPC) (*NPC*1, MIM 257220) disease is a rare, genetically determined, autosomal, recessive neurodegenerative, neurovisceral storage disorder caused by mutations in the *NPC1* gene (95%) or, only rarely, in the *NPC2* gene (5%) that lead to the progressive neurodegeneration of the central nervous system (Sleat et al., 2004; Millat et al., 2005; Vanier, 2010; Newton et al., 2018; Lee et al., 2020). The loss of function of the NPC1 protein results in the impairment of the regulation of cholesterol efflux and the dysfunction of cholesterol homeostasis. The Npc1-mediated dysfunction of lipid transport has severe consequences for all brain cells. Besides the cell-autonomous contribution of neuronal Npc1, aberrant Npc1 signaling in brain cells is critical for the pathology (Malara et al., 2024). The lipid accumulation in the lysosomes and late endosomes is probably the crucial event in the disease pathogenesis, although the underlying mechanisms are not fully understood (Bi and Liao, 2010; Lloyd-Evans and Platt, 2010; Peake et al., 2011; Wheeler and Sillence, 2020; Burbulla et al., 2021). Interestingly, Nguyen et al. (2024) identified that phosphorylation of T286 on CaMKIIα and S1303 on NR2B increased in mutant animals. These phosphosites are said to be crucial to learning and memory and can trigger neuronal death by altering protein–protein interactions. In addition to cholesterol, the *Npc1* lesion has also been shown to affect the metabolism of sphingolipids (Liscum and Klansek, 1998; Vanier and Suzuki, 1998; Vanier, 1999), resulting in intracellular accumulation of unesterified cholesterol and other composites in various tissues, including an accumulation of the gangliosides and sphingolipids in the brain (Siegel and Walkley, 1994; Vanier, 1999; Zervas et al., 2001; Garver et al., 2007). The metabolic changes are accompanied by gliosis and extensive loss of Purkinje cells in the cerebellum and degeneration of other central nervous compartments (Elleder et al., 1985; Pacheco and Lieberman, 2008; Tang et al., 2010; Patterson et al., 2012; Piroth et al., 2017). The clinical manifestations of NPC1 disease vary and often correlate with the age of onset, which can occur from the prenatal period well into adulthood (Wraith et al., 2009; Vanier, 2010; Patterson et al., 2012; Mengel et al., 2017; Patterson et al., 2017; Baxter et al., 2022). The most typical neuropsychiatric symptoms are: cerebellar ataxia, dysarthria, dysphagia, progressive dementia, cataplexy, seizures and dystonia, psychosis, paranoid delusions or schizophrenia and saccadic eye movement abnormalities or vertical supranuclear gaze palsy (Garver et al., 2007; Sévin et al., 2007; Vanier, 2010; Patterson et al., 2012; Mengel et al., 2017; Bonnot et al., 2019; Rego et al., 2019).

Neuropathological changes in the limbic system, the entorhinal area, or the piriform cortex in NPC1 are largely unexplored; only a few studies examined the limbic system and, in particular, the hippocampus in NPC1-diseased patients. Walterfang et al. (2010) studied gray matter volume and white matter structural differences in 6 adult patients with NPC and 18 sex- and age-matched controls. The NPC patients showed bilateral gray matter reductions in large clusters in bilateral hippocampus, thalamus, upper cerebellum and insula, in addition to smaller regions of inferior-posterior cortex. Moreover, patients showed widespread reductions in fractional anisotropy in main pathways of white matter. Subsequent analysis suggests that these changes are caused by both impaired myelination and altered axonal structure. In a more recent study, the same group (Walterfang et al., 2013) compared 10 adult patients with NPC disease (18–49 years of age) with 27 age- and sex-matched controls. Most structures were smaller in patients with NPC (NPC1 and NPC2 not differentiated) compared with controls. The thalamus, hippocampus, and striatum showed the greatest and most significant volume reductions, and the left hippocampal volume correlated with symptom score and cognition. Vertex analysis of the thalamus, hippocampus and caudate implicated regions involved in memory, executive function, and motor control. The results from vertex analyses also showed a significant decrease in volume of the CA1 region and the subiculum (Walterfang et al., 2013). CA3, on the other hand, showed no morphological changes. The nucleus basalis and the septum showed severely atrophied pyramidal cell terminals. The cell bodies were almost completely absent (Ong et al., 2001). Other neuropathological studies in NPC1 patients also showed neurofibrillary tangles in the basal ganglia, brainstem, thalamus, and hippocampus, similar to those found in Alzheimer’s dementia (Love et al., 1995; Suzuki et al., 1997).

So far, there is no causal therapy for NPC1, and the treatment efforts are focused on slowing the disease progression in man and/or mouse (Bräuer et al., 2019; Cariati et al., 2021; Rodriguez-Gil et al., 2021; Baxter et al., 2022; Campbell et al., 2023). Therapy with the iminosugar glucosylceramide synthase inhibitor N-butyldeoxynojirimycin (miglustat, Zavesca^®^) correlates with reduced glycosphingolipid levels, stabilized neurological phenotypes, slowing of disease progression and significantly reduced risk of mortality in NPC1 (Platt and Jeyakumar, 2008; Ginocchio et al., 2013; Fecarotta et al., 2015; Patterson et al., 2020; Curelaru et al., 2021). Miglustat (MIGLU) is the only treatment approved for NPC in Europe, Canada, and Japan (Lachmann and Platt, 2001; Lachmann, 2006; Patterson et al., 2007; Platt and Jeyakumar, 2008; Wraith et al., 2009). A positive effect of cyclodextrin (2-hydroxypropyl-β-cyclodextrin, HPβCD), a cyclic oligosaccharide believed to help transport cholesterol out of the lysosome (Rosenbaum et al., 2010; Taylor et al., 2012), has also been confirmed by clinical studies in NPC1 patients after intrathecal administration (Liu et al., 2010; Ramirez et al., 2010; Matsuo et al., 2013).

In *Npc1^−/−^* mice, a promising therapy is a combination of MIGLU, HPβCD, and the neurosteroid allopregnanolone (ALLO), leading to a prevention of cerebellar Purkinje cell loss, a significantly improved motor function, a reduced intracellular lipid storage, and an increased lifespan (Zervas et al., 2001; Ahmad et al., 2005; Davidson et al., 2009; Hovakimyan et al., 2013a; Maass et al., 2015; Tanaka et al., 2015; Davidson et al., 2016; Meyer et al., 2017; Ebner et al., 2018; Bräuer et al., 2019). In a mouse model, we recently compared the therapeutic effects of the combination therapy (COMBI) with that of MIGLU alone, HPßCD alone, and Sham injections on body and brain weight and the behavior of *Npc1^−/−^* mice in a larger cohort (Holzmann et al., 2021). The results showed that each drug had the potential to significantly improve body weight deficits in *Npc1^−/−^* mice; however, treatments with COMBI, MIGLU and HPßCD did not significantly increased brain weight in *Npc1^−/−^* mice, their values being increased by 3.58, 0.51 and 7.67%, respectively, compared with Sham-treated mutants (Holzmann et al., 2021).

Here, we used the BALB/cNctr-*Npc1*m1N/−J Jackson *Npc1* mouse strain, which carries a spontaneous mutation of *Npc1* and partly displays pathological hallmarks of the human disease (Higashi et al., 1993; Pentchev, 2004; Maass et al., 2015), resulting in weight loss, increased lipid storage, ataxia, and progressive neurodegeneration characterized by cerebral and cerebellar atrophy, hypomyelination, degeneration of neurons in various parts of the brain, the most pronounced being cerebellar Purkinje cells (Morris et al., 1982; Tanaka et al., 1988; Higashi et al., 1993; Zervas et al., 2001; Loftus et al., 2002; Võikar et al., 2002; Sarna et al., 2003; Walkley and Suzuki, 2004; Luan et al., 2008). The cerebral cortex of *Npc1^−/−^* mice showed mild to moderate signs of degeneration seen in the prefrontal cortex by a significant neuron reduction of 28% and volume reduction of 19% (German et al., 2001a,b). Loss of neurons was also observed in the somatosensory and motor cortices (German et al., 2001a,b; Ohara et al., 2004; Pressey et al., 2012), striatum, globus pallidus, medial geniculate body, cerebellar cortex besides the Purkinje neurons and deep cerebellar nuclei (Ong et al., 2001; Maass et al., 2015); these also led to volume reductions (Tanaka et al., 1988; German et al., 2001a,b). Other affected brain regions of *Npc1^−/−^* mice were thalamus and brainstem with loss of neurons in substantia nigra, nucleus tractus solitarii, and locus coeruleus (German et al., 2002; Luan et al., 2008; Chiba et al., 2014).

As far as was studied in various white matter regions of *Npc1^−/−^* mice, reduced volumes were explained by a degeneration of myelin sheaths (Lope-Piedrafita et al., 2008), supporting the assumption that *Npc1* mutation causes hypomyelination (German et al., 2002). A significant reduction of myelination, especially in the olfactory bulb, cerebral cortex, hippocampus, thalamus, and hypothalamus, was reported by Qiao et al. (2018). Most obvious was hypomyelination by the reduction in height of the corpus callosum in cross sections of the *Npc1^−/−^* mouse brain, accompanied by a reduction in the number of glial cells by 63% (German et al., 2001b, 2002; Ahmad et al., 2005).

Although various quantitative data on the brains and parts thereof in untreated *Npc1^−/−^* mice and respective wild types have been described in the last decades (Supplementary Table S1), a systematic evaluation of region- and areal-specific volumes for comparison with effects of drug treatments in *Npc1^+/+^* and *Npc1^−/−^* mice is lacking. Therefore, in the present study, we analyzed volumes of brain regions and areas of the BALB/cNctr-*Npc1*m1N/−J Jackson Npc1 mouse strain in *Npc1*^+/+^ and *Npc1^−/−^* mice that received either Sham or different drug therapies. Importantly, for the first time, *Npc1^−/−^* mice treated with (i) vehicle injection (Sham), (ii) a combination of MIGLU, ALLO, and HPßCD (COMBI), (iii) MIGLU monotherapy (MIGLU), and (iv) HPßCD monotherapy (HPßCD) were included in the evaluation in order to look for drug-related therapeutic effects. For comparison, four respective *Npc1*^+/+^ control mouse groups were studied in parallel. Because no noticeable lateral differences were detected in random measurement, data were evaluated in the right hemispheres only. The most important point, however, is that all data presented are given as fresh volumes, i.e., all measures were corrected for individual brain-specific tissue shrinkage during the histological workup. Accordingly, the aim of the present work is to firstly evaluate which parts of the brain are affected by volume loss in *Npc1^−/−^* mice compared with *Npc1^+/+^* mice, and, secondly, whether volumes of different parts of the brain of *Npc1^−/−^* mice are affected differently by the different therapies. To do so and to get an overview, brains were divided into 13 structural parts and their fresh volumes determined. In addition, due to olfactory (Hovakimyan et al., 2013b; Meyer et al., 2017, 2018; Witt et al., 2018; Bräuer et al., 2019) and learning (Võikar et al., 2002; Hovakimyan et al., 2013a; Bräuer et al., 2019; Holzmann et al., 2021) deficits in *Npc1^−/−^* mice, functionally associated areas were examined more closely.

## 2 Results

**Figure 1:**
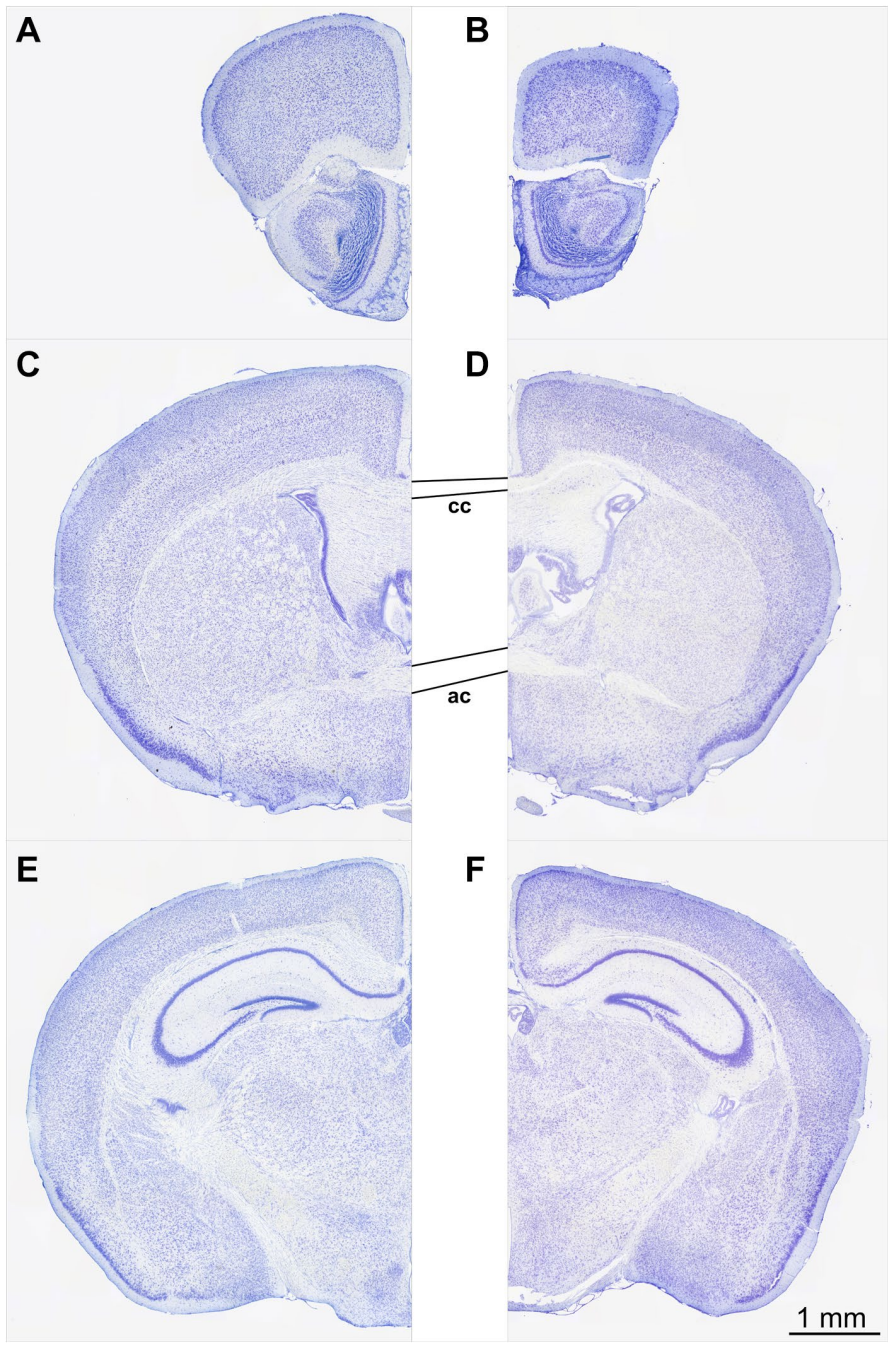
Nissl-stained frontal sections of a *Npc1^+/+^* mouse **(A,C,E)** at the levels with respect to Bregma: +3.08 **(A)**, −0.10 **(C),** and −2.06 **(E)**. The respective sections of the *Npc1^−/−^* mouse **(B,D,F)** were chosen as best corresponding to **(A,C,E)**. **(A,B)** sections at the level of the largest extent of the accessory olfactory bulb, **(C,D)** sections at the level of the largest height of the anterior commissure, **(E,F)** sections at the level of the rostral beginning of the fasciola cinereum. Scale bar: 1 mm for **(A–F)**.

At first glance, the perfusion-fixed brains of *Npc1^−/−^* mice appeared smaller than those of *Npc1^+/+^* mice. The olfactory bulb in particular appeared smaller and the cerebellar folia less prominent. Screening of the Nissl-stained specimens at low microscopic magnification already showed differences in brain cytoarchitecture between *Npc1^−/−^* compared with *Npc1^+/+^* mice (Figures 1A–F). The olfactory bulb of *Npc1^−/−^* mice *in toto* was smaller, mainly due to the reduced white matter structures. Also, the cross-sectional area of the accessory olfactory bulb of *Npc1^−/−^* mice was reduced (Figure 1B). When examining the preparations of the *Npc1^−/−^* mice, the generally narrowed profiles of the subcortical white matter and partly reduced thickness of the cortical areas were particularly noticeable (Figures 1C–F). Moreover, the density of glia cell nuclei in the white matter structures appeared to be lower (Figures 1C–F). As already seen when dissecting the cerebellum out of the skull of *Npc1^−/−^* mice, the respective histological slices showed a smaller cerebellum-containing area compared with *Npc1^+/+^* mice.

In Table 1, the measurements of the analyzed brain structures found in *Npc1^+/+^* and *Npc1^−/−^* mice of the Sham groups are summarized, the data given as fresh volumes (mm^3^), or as maximal heights (mm) for the anterior commissure and the corpus callosum, as seen in frontal sections.

**Table 1:** Mean values ± SEM of the fresh volumes (right hemisphere) and heights of Sham-treated *Npc1*^+/+^ and *Npc1*^−/−^ mice, and relative changes of *Npc1*^−/−^ mice compared with *Npc1*^+/+^ mice.

Parameter	*Npc1*^+/+^	*Npc1*^−/−^	Rel. change
Body weight (g)	24.826 ± 0.573	13.511 ± 0.633^***^	−45.6%
Brain weight (g)	0.474 ± 0.006	0.374 ± 0.007^***^	−21.1%
Brain volume (mm^3^)	0.459 ± 0.006	0.362 ± 0.007 ^***^	−21.1%
Basal forebrain volume (mm^3^)	62.819 ± 1.745	51.833 ± 1.645^***^	−17.5%
CPu + GP + ic volume (mm^3^)	19.910 ± 0.560	15.847 ± 0.528^*^	−20.4%
Mesencephalon volume (mm^3^)	19.556 ± 0.814	14.974 ± 0.768^***^	−23.4%
Brainstem volume (mm^3^)	31.268 ± 0.965	28.044 ± 0.909^*^	−10.3%
Frontal cortex volume (mm^3^)	16.923 ± 0.675	11.999 ± 0.636^***^	−29.1%
Motor cortex volume (mm^3^)	6.394 ± 0.318	3.678 ± 0.300^***^	−42.5%
Sensory cortex volume (mm^3^)	30.082 ± 0.995	21.686 ± 0.939^***^	−27.9%
Insular cortex volume (mm^3^)	5.487 ± 0.417	5.227 ± 0.394	−4.7%
Hippocampus volume (mm^3^)	23.463 ± 0.649	20.667 ± 0.612^**^	−11.9%
Cerebellum volume (mm^3^)	29.196 ± 0.839	21.272 ± 0.791^***^	−27.1%
Ventricle volume (mm^3^)	1.892 ± 0.480	1.635 ± 0.452	−13.6%
White matter volume (mm^3^)	6.558 ± 0.239	3.190 ± 0.225^***^	−51.4%
Anterior commissure volume (mm^3^)	0.470 ± 0.033	0.178 ± 0.032^***^	−62.1%
Corpus callosum height (μm)	418.7 ± 16.8	197.7 ± 17.7^***^	−52.8%
Anterior commissure height (μm)	409.6 ± 21.4	309.9 ± 22.5^**^	−24.3%
Olfactory bulb volume (mm^3^)	8.142 ± 0.219	5.335 ± 0.219^***^	−34.5%
Accessory olfactory bulb vol. (mm^3^)	0.461 ± 0.125	0.364 ± 0.125^***^	−21.0%
Anterior olfactory nucleus vol. (mm^3^)	0.727 ± 0.023	0.601 ± 0.023^***^	−17.3%
Olfactory tubercle volume (mm^3^)	1.890 ± 0.044	1.506 ± 0.046^***^	−20.3%
Lateral olfactory tract volume (mm^3^)	0.350 ± 0.012	0.208 ± 0.012^***^	−40.6%
Piriform cortex volume (mm^3^)	5.077 ± 0.140	3.992 ± 0.148^***^	−21.4%
Medial habenular nucleus vol. (mm^3^)	0.109 ± 0.003	0.099 ± 0.003^*^	−9.1%
Dentate gyrus volume (mm^3^)	2.628 ± 0.076	2.184 ± 0.084^***^	−16.9%
Cornu ammonis field 1 volume (mm^3^)	3.962 ± 0.123	3.071 ± 0.136^***^	−22.5%
Cornu ammonis field 2 + 3 vol. (mm^3^)	2.776 ± 0.086	2.536 ± 0.095	−8.6%
Subiculum volume (mm^3^)	2.222 ± 0.101	1.790 ± 0.111^**^	−19.4%
Pre- + parasubiculum volume (mm^3^)	1.655 ± 0.089	1.459 ± 0.099	−11.8%
Entorhinal cortex volume (mm^3^)	4.863 ± 0.247	3.892 ± 0.274^*^	−20.0%

The double lines delineate the 13 regions into which the whole brains were divided. CPu + GP + ic = caudate putamen + globus pallidus + internal capsule. *Significant difference between Npc1^+/+^ and Npc1^−/−^ mice. ^*^p < 0.05, ^**^p < 0.01, ^***^p < 0.001.

### 2.1 Shrinkage factor

**Figure 2:**
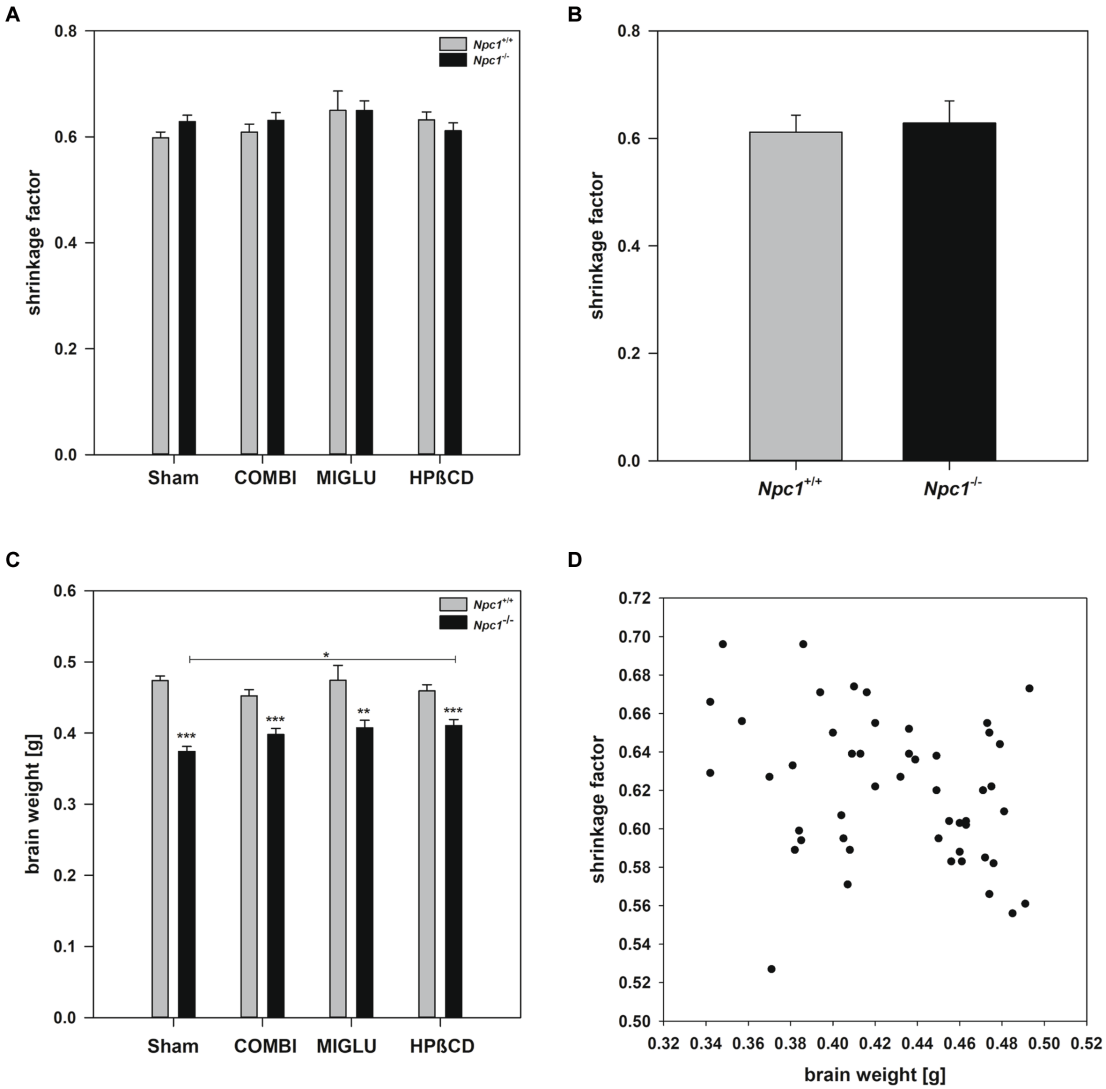
**(A)** Differentiated shrinkage factors of the brains of *Npc1*^+/+^ and *Npc1^−/−^* mice of the Sham, COMBI, MIGLU, and HPßCD groups, and **(B)** mean shrinkage factors of the brains of *Npc1*^+/+^ and *Npc1^−/−^* mice irrespective of treatment. **(C)** Brain weights of *Npc1*^+/+^ and *Npc1^−/−^* mice of the Sham, COMBI, MIGLU, and HPßCD groups, and **(D)** correlation analysis of brain weights and shrinkage factors of all brains investigated. * Significant *post-hoc* tests are indicated by asterisks (*p < 0.05, **p < 0.01, ***p < 0.001). Data are means ± SEM.

In order to obtain a reliable, quantitative fresh volume calculation from paraffin-embedded brain material, the volume of the sectioned brain was corrected for the brain-specific shrinkage (Zilles, 1978; Wree et al., 1981; Beck et al., 1993). The shrinkage factors of the brains of all individual 8 experimental groups did not differ significantly nor did brains of the overall groups of *Npc1^+/+^* versus *Npc1^−/−^* mice showed significant differences (Two-way ANOVA, genotype *p* = 0.553, treatment *p* = 0.430; Figures 2A,B). Testing the correlation between the individual brain weights and the respective individual shrinkage factors in 49 samples (Figure 2D) according to Spearman revealed a correlation coefficient of −0.277, and a *p*-value of 0.0539. However, as there is a low correlation between brain weight and shrinkage factor, it seems justified to speculate about a relationship between both parameters. During embedding of the fixed brains in paraffin wax especially water and lipids were removed by the solvents used. It could be hypothesized that the smaller brains of *Npc1^−/−^* mice, which contain some lipids in lower amounts per gram of brain, but higher concentrations of various proteins, shrink less (Santiago-Mujica et al., 2019; Gläser et al., 2020).

### 2.2 Drug treatment partially altered fresh volumes of brain regions of *Npc1^+/+^* and *Npc1^**−**/**−**^* mice

#### 2.2.1 COMBI treatment

**Figure 3:**
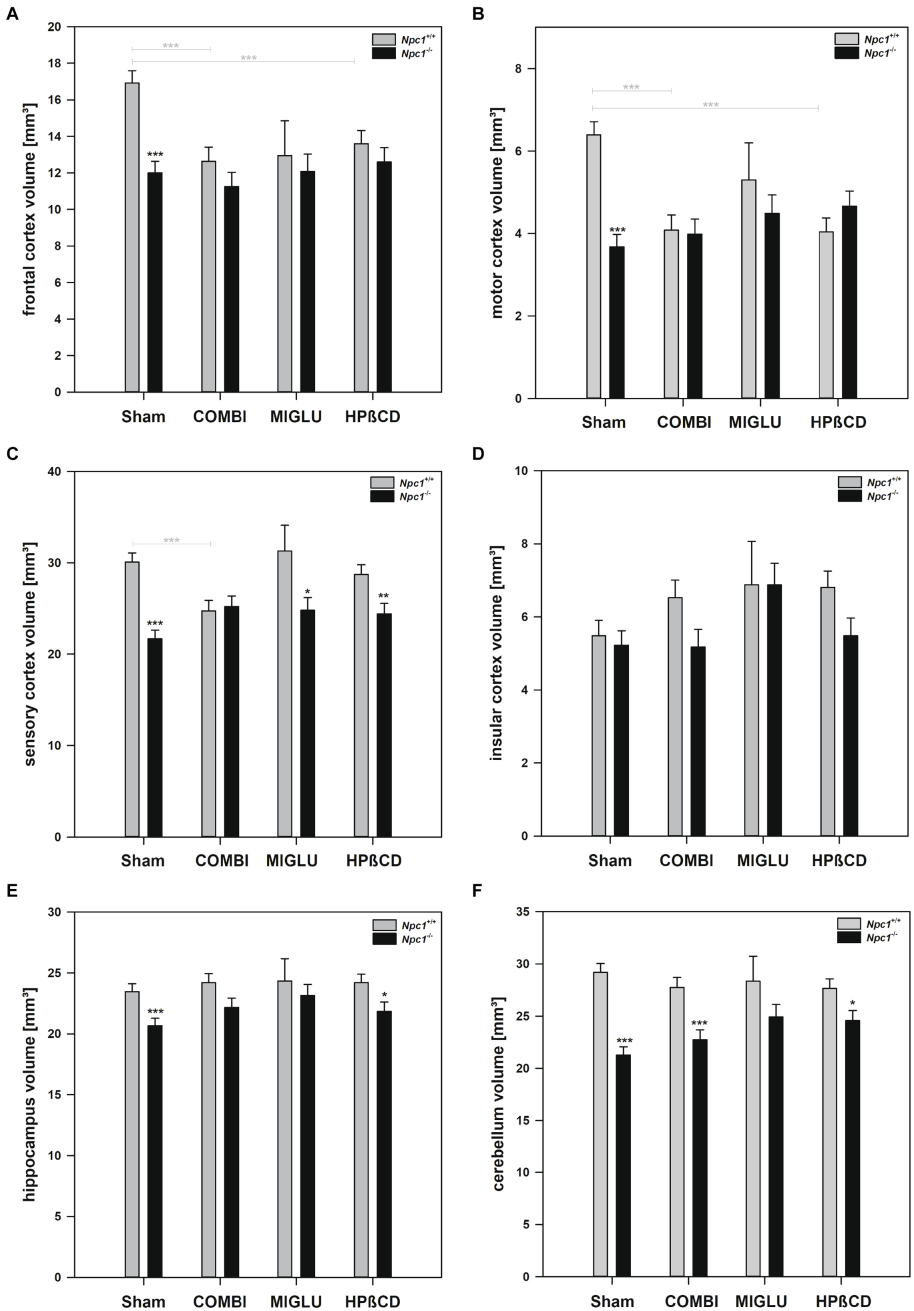
Fresh volumes of **(A)** frontal cortex, **(B)** motor cortex, **(C)** sensory cortex, **(D)** insular cortex, **(E)** hippocampus, and **(F)** cerebellum of the brains of *Npc1*^+/+^ and *Npc1^−/−^* mice of the Sham, COMBI, MIGLU, and HPßCD groups. Significant post-hoc tests are indicated by asterisks (*p < 0.05, **p < 0.01, ***p < 0.001). Data are means ± SEM.

**Figure 4:**
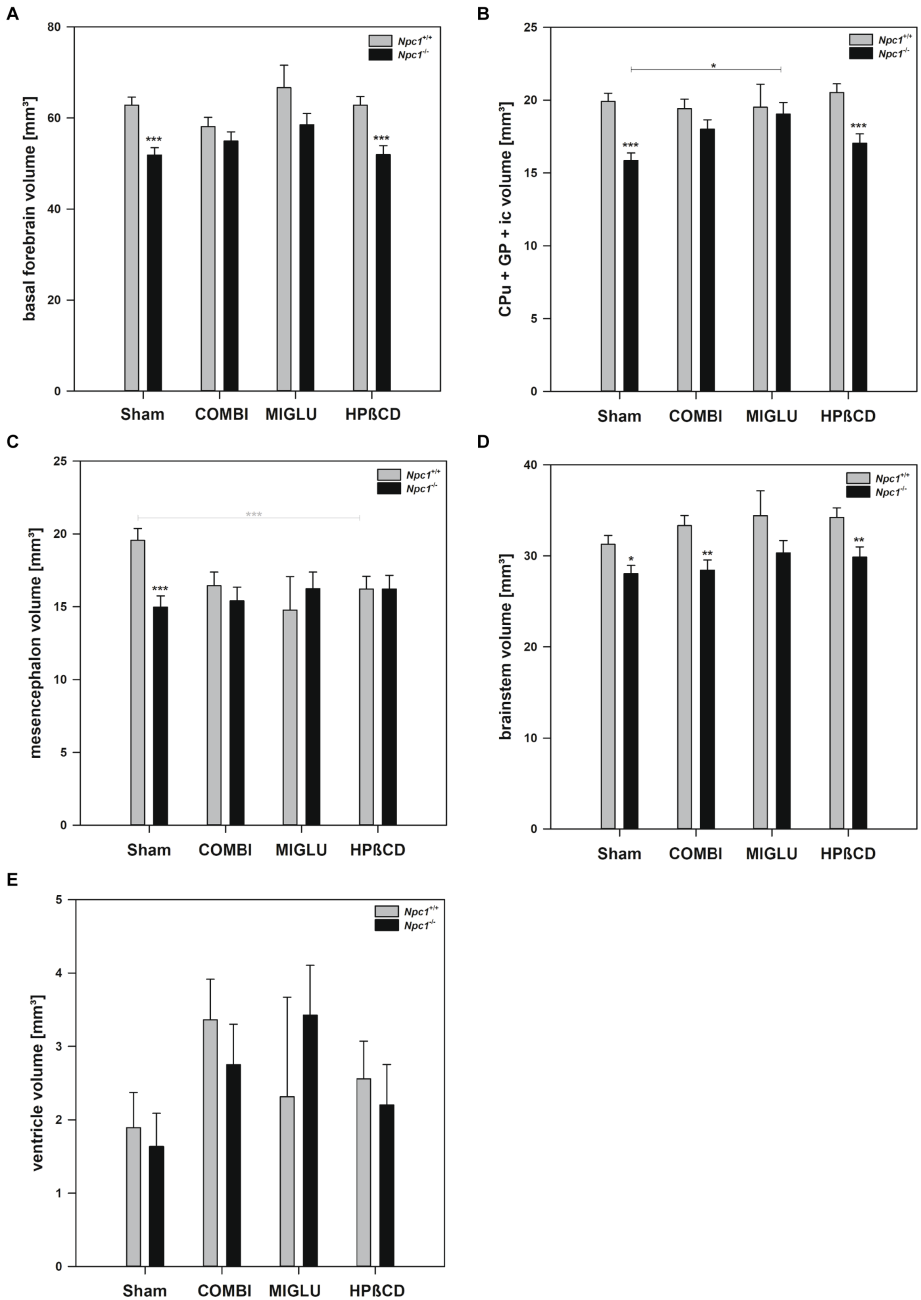
Fresh volumes of **(A)** basal forebrain, **(B)** CPu + GP + ic, **(C)** mesencephalon, **(D)** brainstem, and **(E)** ventricle of the brains of *Npc1*^+/+^ and *Npc1^−/−^* mice of the Sham, COMBI, MIGLU, and HPßCD groups. Significant post-hoc tests are indicated by asterisks (*p < 0.05, **p < 0.01, ***p < 0.001). Data are means ± SEM.

In *Npc1^+/+^* mice, COMBI treatment reduced fresh volumes of the frontal (−25.3%) (Figure 3A), motor (−36.2%) (Figure 3B) and sensory cortices (−17.8%) (Figure 3C) compared with the Sham-treated group; the other regions, however, were left unaltered. In *Npc1^−/−^* mice, COMBI induced in none of the 13 regions significantly changed fresh volumes (Figures 4A–E, 3A–F) compared with Sham treatment. When both COMBI treatment groups were compared, significant differences of fresh volumes between *Npc1^+/+^* and *Npc1^−/−^* mice were found in the brainstem (33.32 ± 1.11 mm^3^ vs. 28.43 ± 1.11 mm^3^), and cerebellum (27.74 ± 0.97 mm^3^ vs. 22.72 ± 0.97 mm^3^).

#### 2.2.2 MIGLU treatment

Compared with Sham treatment, MIGLU treatment of *Npc1^+/+^* mice did not induce significant changes of fresh volumes of any region investigated (Figures 4A–E, 3A–F). In the respective *Npc1^−/−^* mice, MIGLU treatment only resulted in a significant increase of fresh volumes in the CPu + GP + ic region (Sham: 15.85 ± 0.53, MIGLU: 19.04 ± 0.79) (Figure 4B), all other region remained unchanged (Figures 4A,C–E, 3A–F).

#### 2.2.3 HPßCD treatment

*Npc1^+/+^* mice treated with HPßCD showed significantly reduced fresh volumes of the mesencephalon (−17.1%) (Figure 4C), and frontal (−19.6%) (Figure 3A) and motor cortices (−36.9%) (Figure 3B) compared with Sham treatment. In *Npc1^−/−^* mice, no significant changes of fresh volumes of any region compared with the respective Sham group were found (Figures 4A–E, 3A–F). After HPßCD treatment, *Npc1^−/−^* mice still showed significantly lower fresh volumes in the basal forebrain (51.91 ± 2.01 mm^3^ vs. 62.83 ± 1.87 mm^3^), CPu + GP + ic (17.03 ± 0.65 mm^3^ vs. 20.52 ± 0.60 mm^3^), brainstem (29.87 ± 1.11 mm^3^ vs. 34.23 ± 1.03 mm^3^), sensory cortex (24.40 ± 1.15 mm^3^ vs. 28.72 ± 1.06 mm^3^), hippocampus (21.86 ± 0.75 mm^3^ vs. 24.21 ± 0.69 mm^3^), and cerebellum (24.56 ± 0.97 mm^3^ vs. 27.65 ± 0.90 mm^3^) compared with the corresponding *Npc1^+/+^* mice.

### 2.3 Fresh volumes of the white matter structures were reduced in *Npc1*^−/−^ mice

#### 2.3.1 COMBI treatment

**Figure 5:**
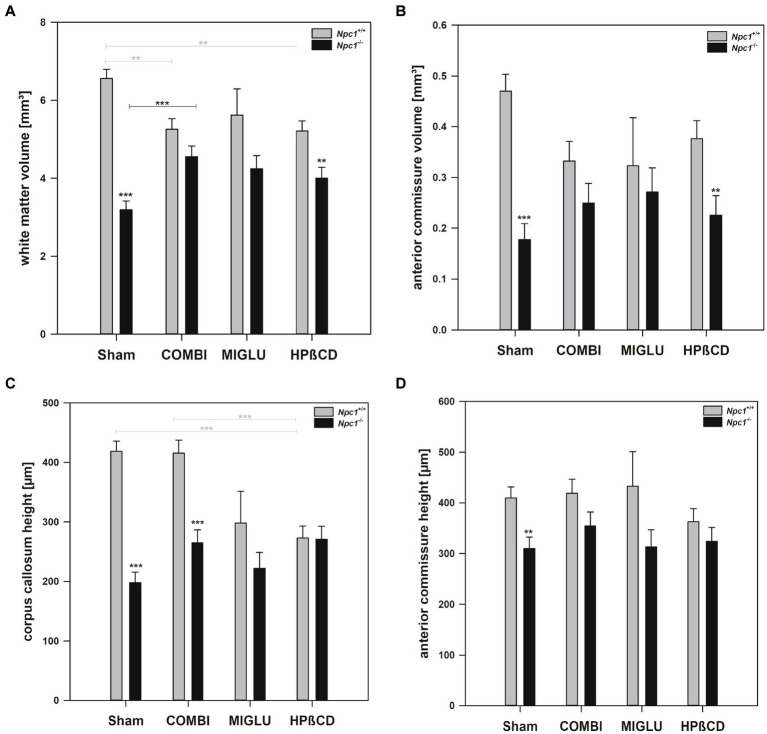
Fresh volumes of **(A)** white matter and **(B)** anterior commissure; **(C)** maximal heights of corpus callosum, and **(D)** maximal heights of anterior commissure of the brains of *Npc1*^+/+^ and *Npc1^−/−^* mice of the Sham, COMBI, MIGLU, and HPßCD groups. Significant post-hoc tests are indicated by asterisks (*p < 0.05, **p < 0.01, ***p < 0.001). Data are means ± SEM.

In *Npc1^+/+^* mice, COMBI treatment reduced the fresh volume of the white matter (−19.9%) (Figure 5A) compared with the Sham-treated group. In *Npc1^−/−^* mice, COMBI induced a significant increase in the white matter fresh volume (+ 42.8%) (Figure 5A) compared with Sham treatment. When white matter and anterior commissure parameters of both COMBI treatment groups were compared, significant differences were found only in the height of the corpus callosum of *Npc1^+/+^* and *Npc1^−/−^* mice (415.57 ± 21.77 μm vs. 264.95 ± 21.77 μm) (Figure 5C).

#### 2.3.2 MIGLU treatment

Compared with Sham treatment, MIGLU treatment of neither *Npc1^+/+^* nor *Npc1^−/−^* mice significantly changed white matter and anterior commissure parameters (Figures 5A–D).

#### 2.3.3 HPßCD treatment

*Npc1^+/+^* mice treated with HPßCD showed a significantly reduced fresh volume of the white matter (−20.5%) (Figure 5A), and corresponding reduced corpus callosum height (−34.8%, Figure 5C), compared with Sham treatment. In *Npc1^−/−^* mice we found no significant changes in white matter and anterior commissure parameters compared with the respective Sham group (Figures 5A–D). After HPßCD treatment *Npc1^−/−^*, mice still showed significantly lower fresh volumes of the white matter (4.01 ± 0.28 mm^3^ vs. 5.21 ± 0.26 mm^3^) and the anterior commissure (0.23 ± 0.04 mm^3^ vs. 0.38 ± 0.04 mm^3^) compared with the corresponding *Npc1^+/+^* mice.

### 2.4 Fresh volumes of all olfactory areas were reduced in *Npc1*^−/−^ mice compared with the respective sham-treated *Npc1*^+/+^ mice

#### 2.4.1 Sham treatment

**Figure 6:**
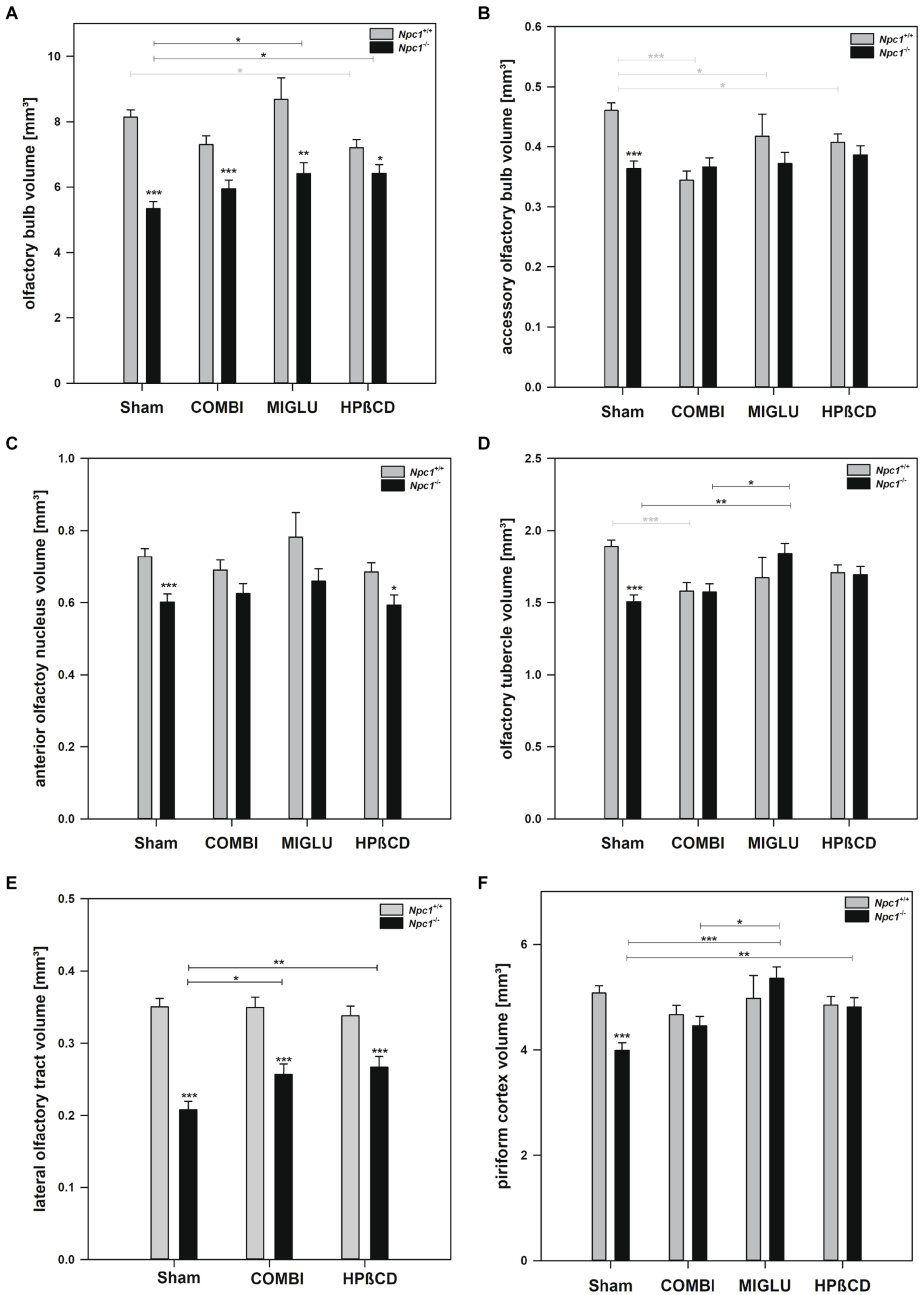
Fresh volumes of olfactory structures of the brains of *Npc1*^+/+^ and *Npc1^−/−^* mice of the Sham, COMBI, MIGLU, and HPßCD groups: **(A)** olfactory bulb, **(B)** accessory olfactory bulb, **(C)** anterior olfactory nucleus, **(D)** olfactory tubercle, **(E)** lateral olfactory tract, and **(F)** piriform cortex. Significant post-hoc tests are indicated by asterisks (*p < 0.05, **p < 0.01, ***p < 0.001). Data are means ± SEM.

In the Sham groups, all olfactory regions investigated showed significantly smaller fresh volumes in *Npc1^−/−^* mice compared with the corresponding *Npc1^+/+^* ones: olfactory bulb (−34.5%) (Figure 6A), accessory olfactory bulb (−21.0%) (Figure 6B), anterior olfactory nucleus (−17.3%) (Figure 6C), olfactory tubercle (−20.3%) (Figure 6D), lateral olfactory tract (−40.6%) (Figure 6E), and piriform cortex (−21.4%) (Figure 6F).

#### 2.4.2 COMBI treatment

COMBI treatment of *Npc1^+/+^* mice reduced fresh volumes of the accessory olfactory bulb (−25.4%) (Figure 6B) and the olfactory tubercle (−16.4%) (Figure 6D). All other olfactory subregions of *Npc1^+/+^* mice were left unaffected in these mice (Figures 6A,C,D). In the COMBI-treated *Npc1^−/−^* mice, a significant increase of fresh volume compared with the Sham group was measured in the lateral olfactory tract only (Figures 6A–F). When the COMBI treatment groups of *Npc1^+/+^* and *Npc1^−/−^* mice were compared, significant differences of fresh volumes were only found in the olfactory bulb (7.29 ± 0.27 mm^3^ vs. 5.95 ± 0.27 mm^3^), and the lateral olfactory tract (0.349 mm^3^ vs. 0.257 mm^3^).

#### 2.4.3 MIGLU treatment

Compared with Sham treatment, MIGLU treatment of *Npc1^+/+^* mice resulted in a significant decrease of the fresh volume of the accessory olfactory bulb (Figure 6B). In *Npc1^−/−^* mice, MIGLU treatment induced an increase of fresh volumes in the olfactory bulb, olfactory tubercle and the piriform cortex compared with the respective Sham group (Figures 6A,D,F). Comparison of the MIGLU treatment groups of *Npc1^+/+^* and *Npc1^−/−^* mice revealed a significant difference of fresh volumes in the olfactory bulb only (8.69 ± 0.66 mm^3^ vs. 6.41 ± 0.33 mm^3^).

#### 2.4.4 HPßCD treatment

HPßCD-treated *Npc1^+/+^* mice showed significantly reduced fresh volume in the olfactory bulb (−11.6%) (Figure 6B) and the accessory olfactory bulb (−11.7%) (Figure 6A) compared with Sham treatment. In *Npc1^−/−^* mice, significant increases of fresh volumes were found in the olfactory bulb (+20.3%) (Figure 6A), the lateral olfactory tract (+28.4%) (Figure 6E), and piriform cortex (+20.5%) (Figure 6F) compared with the respective Sham group. After HPßCD treatment, *Npc1^+/+^* mice still had significantly lower fresh volumes in the olfactory bulb (6.42 ± 0.27 mm^3^ vs. 7.20 ± 0.25 mm^3^), anterior olfactory nucleus (0.593 ± 0.028 mm^3^ vs. 0.685 ± 0.026 mm^3^), and lateral olfactory tract (0.267 ± 0.015 mm^3^ vs. 0.338 ± 0.013 mm^3^) compared with the corresponding *Npc1^+/+^* mice.

#### 2.4.5 All treatments

Irrespective of the drug applied, fresh volumes of the olfactory bulb and the lateral olfactory tract of *Npc1^+/+^* mice significantly exceeded those of the corresponding volumes of *Npc1^−/−^* mice (Figures 6A,E). In all other structures, the corresponding fresh volumes of *Npc1^+/+^* mice did not differ significantly from the respective data of the *Npc1^−/−^* mice (Figures 6B–D,F).

### 2.5 COMBI and MIGLU treatments partly increased fresh volumes of some hippocampal areas of *Npc1*^−/−^ mice

**Figure 7:**
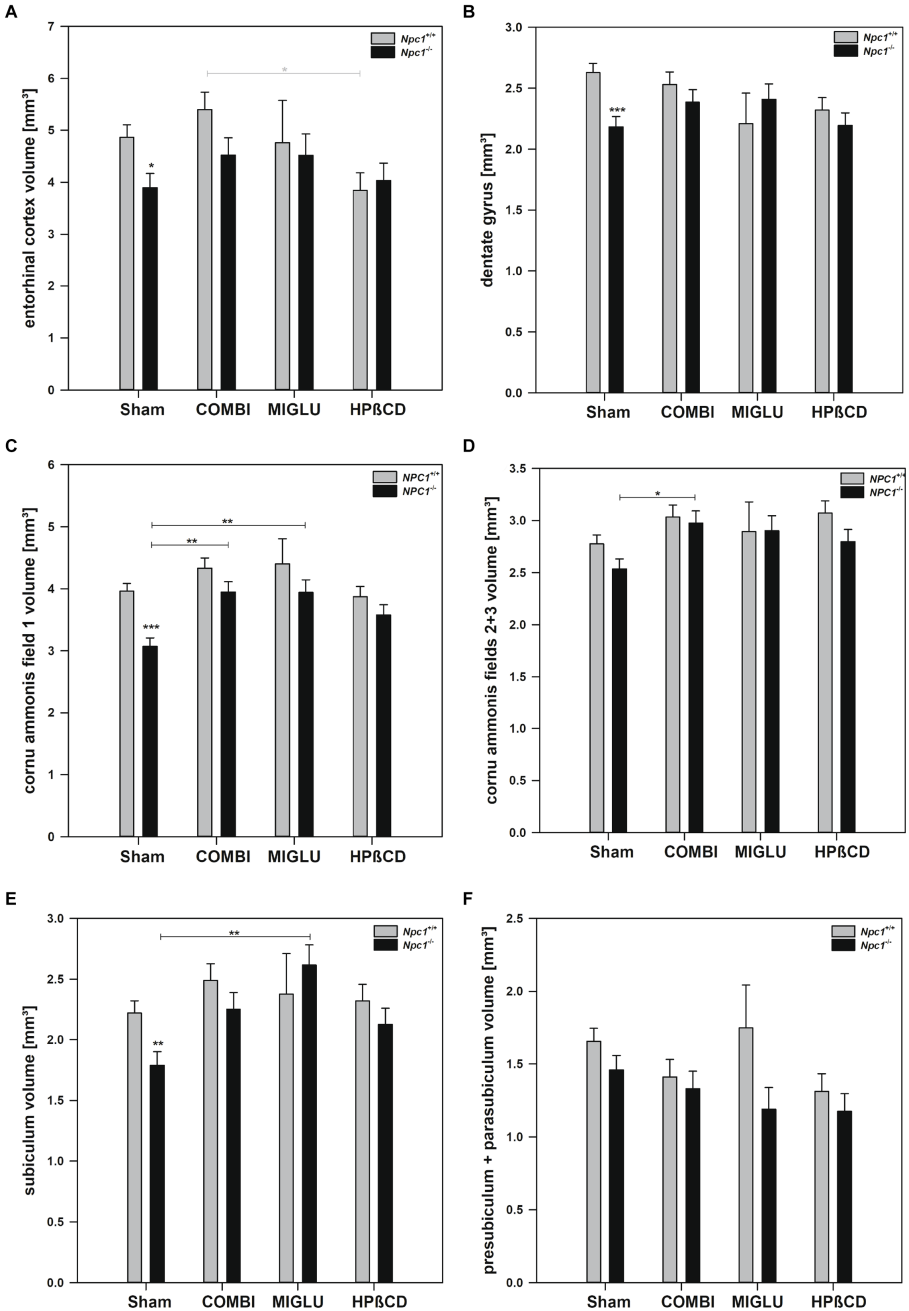
Fresh volumes of hippocampal areas of the brains of *Npc1*^+/+^ and *Npc1^−/−^* mice of the Sham, COMBI, MIGLU, and HPßCD groups: **(A)** entorhinal cortex, **(B)** dentate gyrus, **(C)** cornu ammonis field 1, **(D)** cornu ammonis fields 2 + 3, **(E)** subiculum, and **(F)** presubiculum + parasubiculum. Significant post-hoc tests are indicated by asterisks (*p < 0.05, **p < 0.01, ***p < 0.001). Data are means ± SEM.

After Sham treatment, *Npc1^+/+^* mice had significantly larger fresh volumes than *Npc1^−/−^* mice in 4 of the 6 limbic areas investigated: entorhinal cortex (4.86 ± 0.25 mm^3^ vs. 3.89 ± 0.27 mm^3^), dentate gyrus (2.63 ± 0.08 mm^3^ vs. 2.18 ± 0.08 mm^3^), cornu ammonis field 1 (3.96 ± 0.12 mm^3^ vs. 3.07 ± 0.14 mm^3^), and subiculum (2.22 ± 0.10 mm^3^ vs. 1.79 ± 0.11 mm^3^) (Figures 7A–C,E). Respective measurements of cornu ammonis fields 2 + 3 (2.78 ± 0.09 mm^3^ vs. 2.54 ± 0.10 mm^3^) and presubiculum + parasubiculum (1.66 ± 0.09 mm^3^ vs. 1.49 ± 0.10 mm^3^) did not differ significantly (Figures 7D,F).

#### 2.5.1 COMBI treatment

COMBI treatment of *Npc1^+/+^* mice had no significant effects on fresh volumes of the limbic areas (Figures 7A–F). In the COMBI-treated *Npc1^−/−^* mice, a significant increase of fresh volume compared with the Sham group was measured in the cornu ammonis field 1 by +28.5% and field 2 + 3 by +17.3% (Figures 6C,D). When comparing COMBI treatment groups of *Npc1^+/+^* and *Npc1^−/−^* mice, no significant differences in fresh volumes of hippocampal areas were detected (Figures 7A–F).

#### 2.5.2 MIGLU treatment

Compared with Sham treatment, MIGLU treatment of *Npc1^+/+^* mice resulted in no significant changes of fresh volumes of either limbic region (Figures 7A–F). In respective *Npc1^−/−^* mice, significant MIGLU-based increases of fresh volumes compared with the Sham group was found in cornu ammonis field 1 (+ 28.3%) and subiculum (+ 46.1%) (Figures 7C,E). When comparing MIGLU treatment groups of *Npc1^+/+^* and *Npc1^−/−^* mice, there were no significant differences in the fresh volumes of the hippocampal areas (Figures 7A–F).

#### 2.5.3 HPßCD treatment

Compared with Sham treatment, HPßCD treatment did not significantly change fresh volumes in either hippocampal region of *Npc1^+/+^* mice (Figures 7A–F). The same held true for *Npc1^−/−^* mice (Figures 7A–F). Compared to the Sham-treated *Npc1^−/−^* mice, after HPßCD treatment *Npc1^−/−^* mice did not show any further significantly lower fresh volumes in the entorhinal cortex, the dentate gyrus, the cornu ammonis field 1, and the subiculum (Figures 7A–C,E).

### 2.6 The relative volumes of regions and areas of *Npc1*^+/+^ and *Npc1*^−/−^ mice partly differed

A comparison of relative volumes of regions and areas of *Npc1^+/+^* and *Npc1^−/−^* mice can shed light on whether structures are equally altered in both groups or whether part of the brain of *Npc1^−/−^* mice is significantly larger or smaller.

#### 2.6.1 Sham treatment of *Npc1*^+/+^ and *Npc1*^−/−^ mice

**Figure 8:**
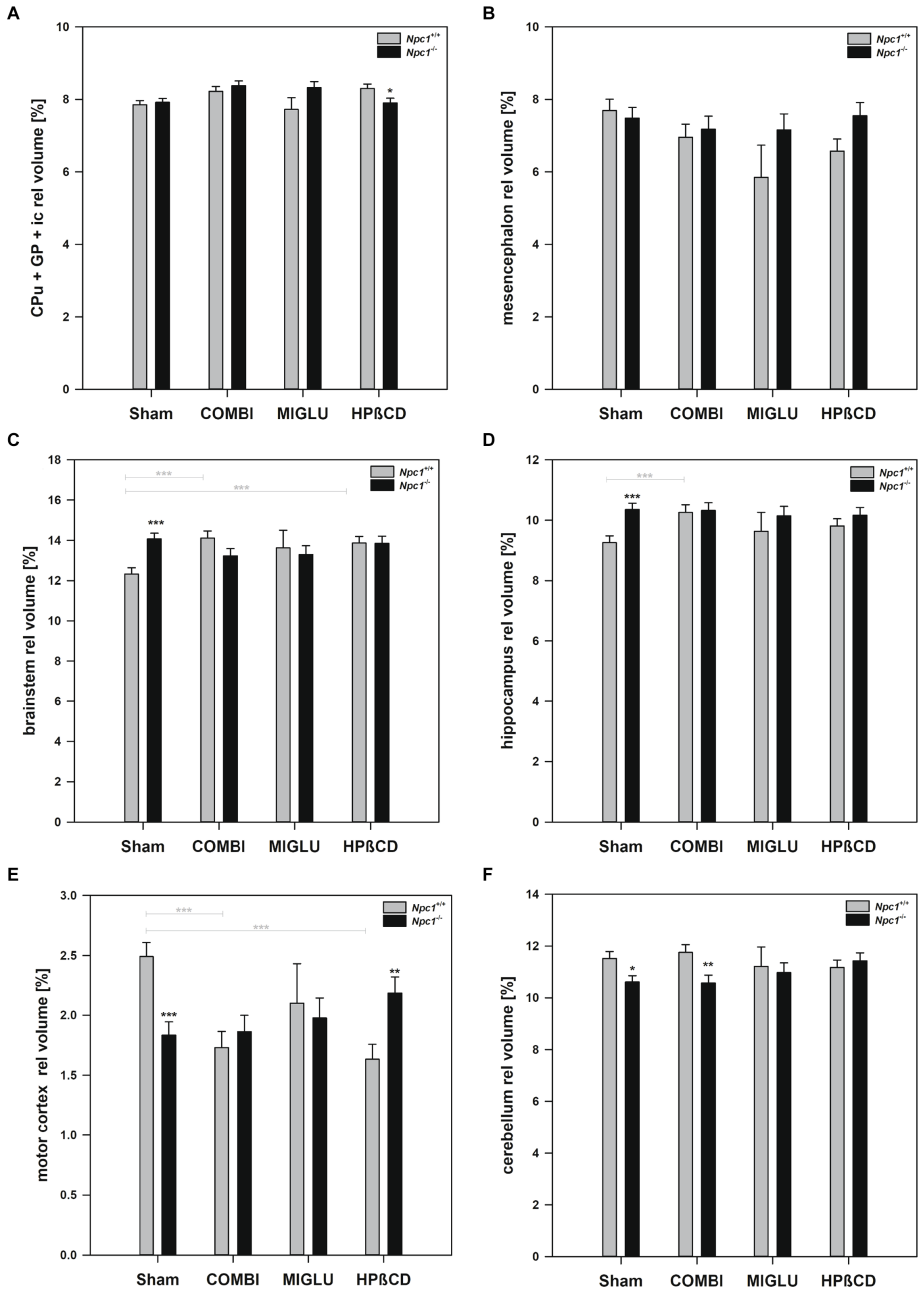
Relative fresh volumes of regions and areas of the brains of *Npc1*^+/+^ and *Npc1^−/−^* mice of the Sham, COMBI, MIGLU, and HPßCD groups: **(A)** CPu + GP + ic, **(B)** mesencephalon, **(C)** brainstem, **(D)** hippocampus, **(E)** motor cortex, and **(F)** cerebellum. Significant post-hoc tests are indicated by asterisks (*p < 0.05, **p < 0.01, ***p < 0.001). Data are means ± SEM.

**Figure 9:**
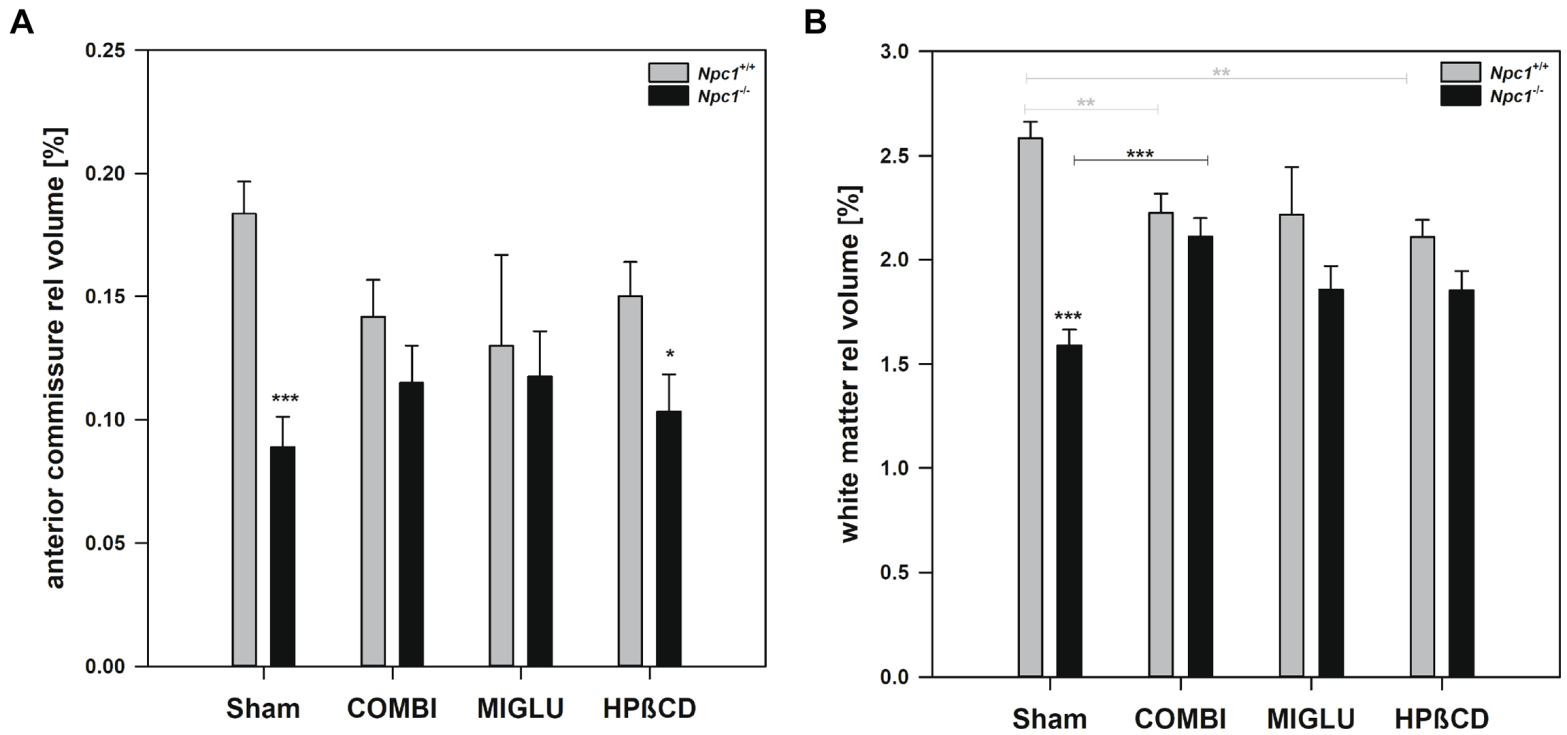
Relative fresh volumes of white matter structures of the brains of *Npc1*^+/+^ and *Npc1^−/−^* mice of the Sham, COMBI, MIGLU, and HPßCD groups: **(A)** white matter, and **(B)** anterior commissure. Significant post-hoc tests are indicated by asterisks (*p < 0.05, **p < 0.01, ***p < 0.001). Data are means ± SEM.

The relative volumes of Sham-treated *Npc1^+/+^* and *Npc1^−/−^* mice showed different patterns in different regions and areas. One pattern, seen in the CPu + GP + ic region and the mesencephalon, showed nearly identical relative volumes in both groups (Figures 8A,B). However, in the brainstem and hippocampus, significantly larger relative volumes were found in *Npc1^−/−^* mice compared with *Npc1^+/+^* mice (Figures 8C,D). In contrast, significantly smaller relative volumes were observed in the motor cortex and cerebellum in *Npc1^−/−^* mice (Figures 8E,F). The most pronounced differences, however, were found in the neuron-free areas of the brain. In the Sham-treated groups, the relative volumes of the white matter were 2.58 ± 0.08% in *Npc1^+/+^* mice, and 1.59 ± 0.08% in *Npc1^−/−^* mice (Figure 9A). In these groups, the anterior commissure showed an even more pronounced difference in relative volumes: in *Npc1^+/+^* mice, the anterior commissure had a relative volume of 0.18 ± 0.01%, the respective *Npc1^−/−^* mice 0.09 ± 0.01% (Figure 9B).

#### 2.6.2 Drug treatment of *Npc1*^+/+^ and *Npc1*^−/−^ mice

The region- and area-specific relative volumes of *Npc1^+/+^* and *Npc1^−/−^* mice partly differed in the drug-treated groups. In the areas where there was no difference in relative volume in both Sham groups, drug treatment did not significantly change the results (Figures 8A,B). If the Sham-treated *Npc1^−/−^* mice had higher relative volumes, drug treatment abolished these differences, resulting in non-significant differences in the COMBI, MIGLU, and HPßCD groups (Figures 8C,D). Even where the Sham-treated *Npc1^−/−^* mice had smaller relative fresh volumes, the differences within the group were largely offset by treatment with COMBI, MIGLU and HPßCD (Figures 8E,F, 9A,B).

### 2.7 First results of mass spectrometric lipid imaging for the *Npc1*^−/−^ mouse model

**Figure 10:**
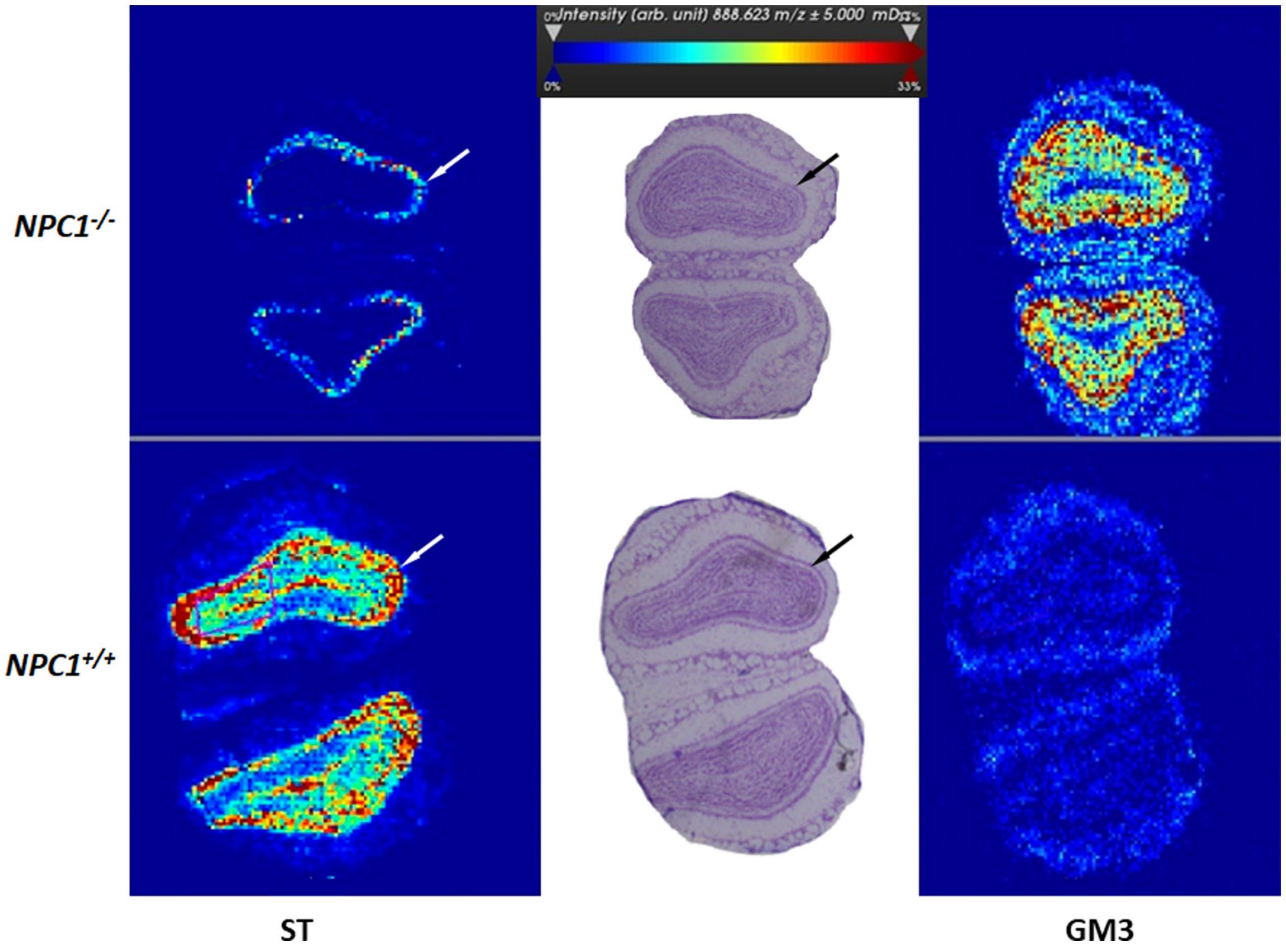
Mass spectrometric lipid imaging of the central olfactory bulb of *Npc1*^−/−^ and *Npc1*^+/+^ mice of the Sham treatment groups. ST (18:1/24:1) - Sulfatide lipid involved in myelin synthesis is minimally synthesized in *Npc1*^−/−^ mice, whereas monosialoganglioside (NeuAc-Gal-Glc-ceramide) GM3 is strongly increased in *Npc1*^−/−^ mice. In both images, differences between *Npc1*^+/+^ and *Npc1*^−/−^ mice are especially seen in the inner plexiform and the granular layers (arrows). The color bar indicates normalized lipid ion intensities (arbitrary units). Resolution: 30 μm per pixel.

Lipids represent the largest component of the brain (Li et al., 2017). With the aim of a better understanding of the molecular details of Npc1 disease, and as a proof of principle, we evaluated lipid distribution and changes in tissue sections of the olfactory bulb. Apparently, the densities of different lipids are changed differently upstream and downstream of the functionally active or non-functional Npc1 protein. As results, we show here the first measurements of ST (18.1/24.1), a sulfatide lipid involved in myelin synthesis (ST) (Figure 10) and the monosialoganglioside (NeuAc-Gal-Glc-ceramide) (GM3) (Figure 10). GM3 was chosen especially because its concentration is known to be increased in *Npc1*^−/−^ mice (Davidson et al., 2009; Tobias et al., 2018). Both lipids represented a clear, layer-specific density distribution in *Npc1*^−/−^ and *Npc1*^+/+^ mice. ST is depleted in the olfactory bulbs of *Npc1*^−/−^ mice, in contrast to high densities in the respective layers in *Npc1^+/+^* mice. In contrast, GM3 is found in high densities in the *Npc1*^−/−^ mice, whereas low densities were exhibited in *Npc1^+/+^* mice. The differences seen in the neuron-containing layers of the olfactory bulb were statistically significant (*p* < 0.001).

## 3 Discussion

### 3.1 Fresh volumes of most regions and areas were reduced in *Npc1*^−/−^ mice

**Figure 11:**
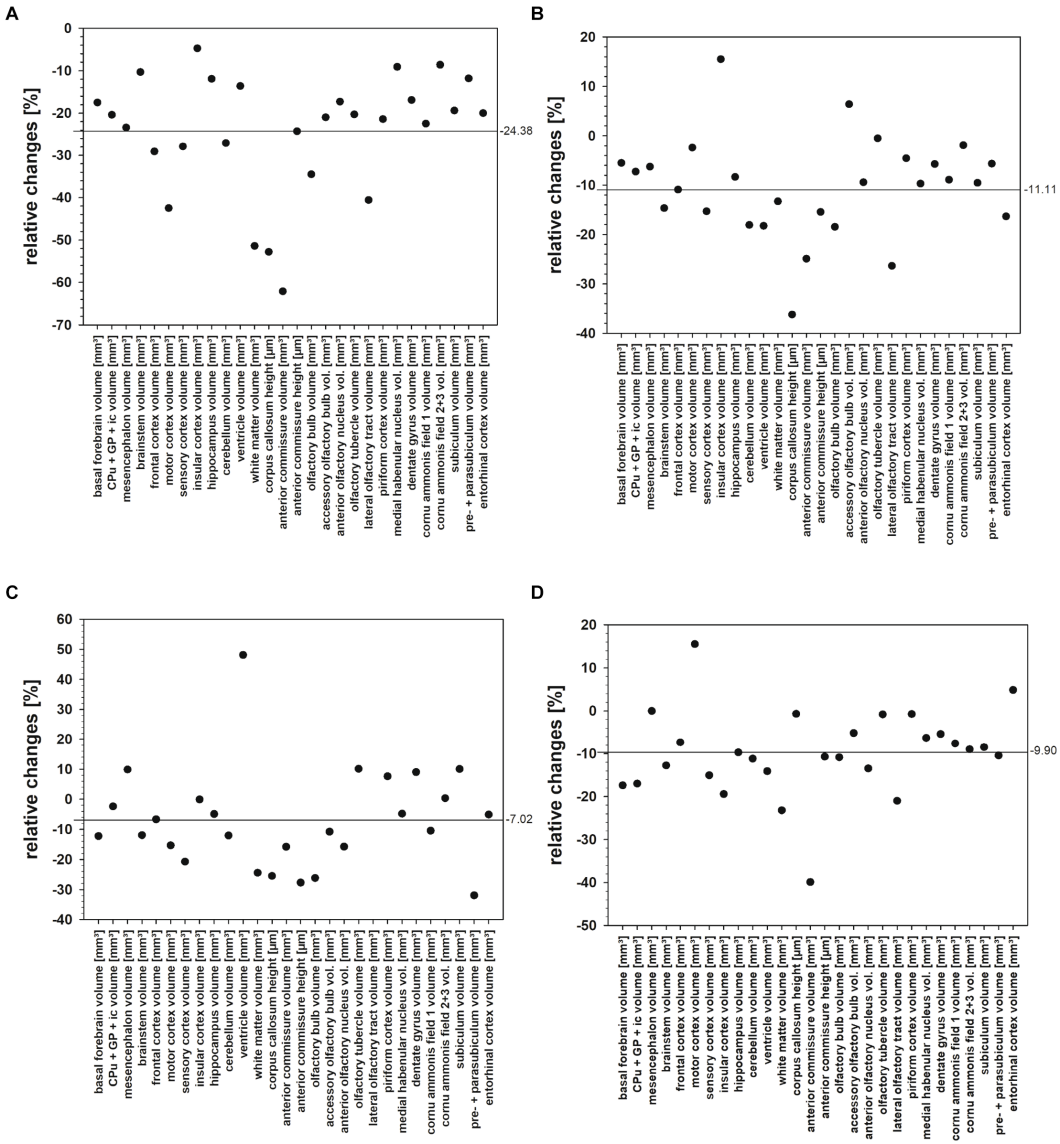
**(A–D)** Relative changes of fresh volume and heights of brain regions and areas of the *Npc1^−/−^* mice compared with the respective *Npc1^+/+^* mice of the Sham, COMBI, MIGLU, and HPßCD treatment groups, calculated as (*Npc1^−/−^* minus *Npc1^+/+^*)/Npc1*^+/+^*. **(A)** Sham treatment, **(B)** COMBI treatment, **(C)** MIGLU treatment, **(D)** HPßCD treatment. The horizontal lines in A-D give the mean values of the relative changes found in the respective treatment groups.

The present study quantified for the first time fresh volumes of 13 regions that encompass the entire brains of *Npc1^−/−^* mice and *Npc1^+/+^* mice, and specifically olfactory and hippocampal areas. It is important to determine fresh volumes as this parameter is independent of the unavoidable shrinkage of brain tissue due to histological procedures. Compared with the Sham-treated *Npc1^+/+^* mice, in the significantly smaller brains of Sham-treated *Npc1^−/−^* mice all regions and areas showed reduced fresh volumes, the differences mostly reaching statistical significance (Table 1). Only a few regions were not significantly reduced in volume, these were those of the insular cortex, the ventricles, the CA2 + 3, and the pre- + parasubiculum (Table 1). The extent of the affectedness of the various parts of the brains of *Npc1^−/−^* mice is illustrated in Figure 11. Compared with *Npc1^+/+^* mice, the mean relative change of brain parts in *Npc1^−/−^* mice was about −24% (Figure 11A); however, the relative changes in the structures of *Npc1^−/−^* mice ranged from −4.7 of the volume of insular cortex (Table 1; Figure 11A) to −62.1% of the volume of the anterior commissure (Table 1; Figure 11A). Interestingly, the motor cortex (−42.5%) (Table 1; Figure 11A), the cerebellum (−27.1%) (Table 1; Figure 11A), the anterior commissure (−62.1%) (Table 1; Figure 11A), the subcortical white matter (−51.4%) (Table 1; Figure 11A), and the lateral olfactory tract (−40.6%) (Table 1; Figure 11A) were most strongly affected in *Npc1^−/−^* mice.

If one calculates the relative proportions of the individual brain regions in the entire brain of the *Npc1*^+/+^ and *Npc1*^−/−^ mice, only a slightly different picture emerges. In many brain regions of Sham-treated *Npc1^−/−^* mice, the relative volumes were in the range of the values calculated in Sham-treated *Npc1^+/+^* mice. Specifically, again the motor cortex (*Npc1^−/−^* mice 1.83%, *Npc1^+/+^* mice 2.49%) (Figure 8E), the cerebellum (*Npc1^−/−^* mice 10.61%, *Npc1^+/+^* mice 11.52%) (Figure 8F), the anterior commissure (*Npc1^−/−^* mice 0.09%, *Npc1^+/+^* mice 0.18%) (Figure 9A), and the subcortical white matter (*Npc1^−/−^* mice 1.59%, *Npc1^+/+^* mice 2.58%) (Figure 9B) were most strongly affected in *Npc1^−/−^* mice.

### 3.2 Hypomyelination is probably a main reason for reduced fresh volumes in *Npc1*^−/−^ mice

Generally, brain volume is mainly dependent on volumes of neurons, glia and neuropil. Thus, lower volumes of brains and parts thereof of *Npc1^−/−^* mice will depend on one or more of these parameters. However, in *Npc1*^+/+^ and *Npc1*^−/−^ mutants, there are actually no published studies available in which quantitative data of the complete brain and its parts with respect to fresh volumes, nerve and glia cell counts, or degree of myelination are presented. It is well known that *Npc1*^−/−^ mice have lower brain weights than the *Npc1*^+/+^ controls (Tanaka et al., 1988; Xie et al., 1999; German et al., 2001a; Loftus et al., 2002; Beltroy et al., 2005; Li et al., 2005; Luan et al., 2008; Maass et al., 2015; Holzmann et al., 2021; Antipova et al., 2022). Also, reduced cell counts of neuron, besides the mostly affected cerebellum (Xie et al., 1999; German et al., 2001b; Griffin et al., 2004; Ko et al., 2005; Li et al., 2005; Repa et al., 2007; Smith et al., 2009; Ramirez et al., 2010; Byun et al., 2011; Lopez et al., 2012; Williams et al., 2014; Maass et al., 2015; Praggastis et al., 2015; Chandler et al., 2017), were dealt with for some specific brain regions, among others for the motor cortex (Tanaka et al., 1988; Xie et al., 1999; German et al., 2001b; Baudry et al., 2003; Beltroy et al., 2005; Repa et al., 2007; Ramirez et al., 2010; Maass et al., 2015), thalamus (Baudry et al., 2003), hippocampus (German et al., 2001b; Baudry et al., 2003; Byun et al., 2011, 2013), medial septum (Cabeza et al., 2012), striatum (German et al., 2001b), and prefrontal cortex (German et al., 2001b; Supplementary Table S1). To what extent the reduced number of neurons and/or their possibly altered dendritic trees (Kulkarni and Firestein, 2012; Kavetsky et al., 2019; Boyle et al., 2020; Kim et al., 2023) or axons (Tanaka et al., 1988; Zervas et al., 2001; Cabeza et al., 2012; Praggastis et al., 2015) contribute to volume reductions in specific brain areas should be clarified in future studies.

**Figure 12:**
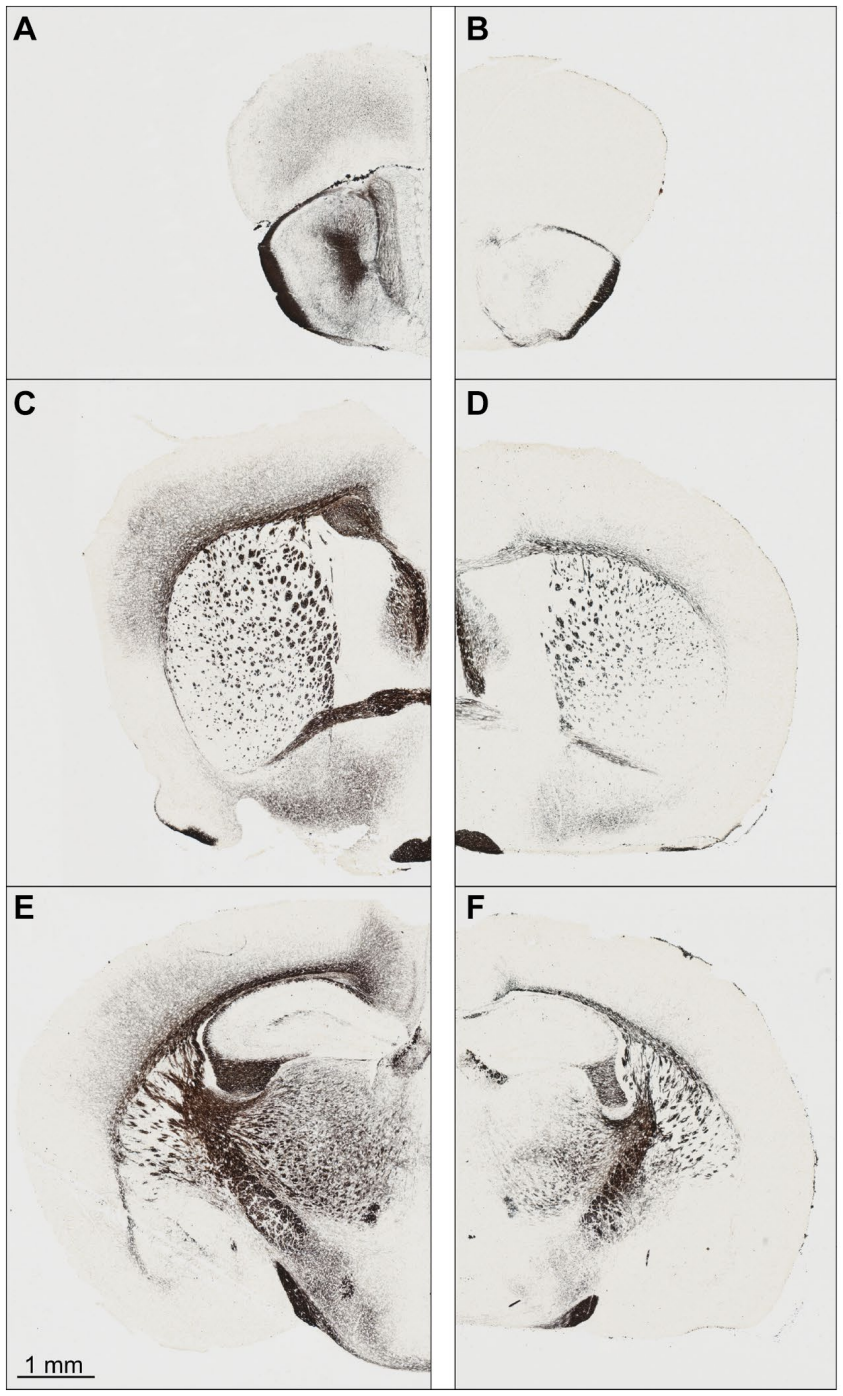
Myelin staining by the Gallyas method of frontal sections of an *Npc1^+/+^* mouse **(A,C,E)** at the levels with respect to Bregma: +3.08 **(A)**, −0.10 **(C)** and − 2.06 **(E)**. The respective sections of the *Npc1^−/−^* mouse **(B,D,F)** were chosen as best corresponding to **(A,C,E)**. **(A,B)** Section at the level of the largest extent of the accessory olfactory bulb, **(C,D)** section at the level of the largest height of the anterior commissure, **(E,F)** section at the level of the rostral beginning of the fasciola cinereum. The scale bar presents 1 mm for **(A–F)**.

As already shown by others (Yan et al., 2011; Yang et al., 2018, 2019; Bernardo et al., 2021; Colombo et al., 2021; Kunkel et al., 2023) hypomyelination can be found in nearly all brain regions of *Npc1^−/−^* mice (Figures 12B,D,F) compared with the respective *Npc1*^+/+^ controls (Figures 12A,C,E). In the Gallyas-stained sections, the reduced myelin staining is most obviously seen in the lateral olfactory tract and other laminae of the olfactory bulb, the subcortical white matter, and the anterior commissure. The nearly missing myelin staining of cortical fibers in the motor, somatosensory (Figure 12D) and retrosplenial cortices (Figure 12F) most probably contributes to their volume reduction. These qualitative reductions seen in the myelin staining are seemingly well in line with the reduction of their fresh volumes.

In particular the nearly perikarya-free structures containing axons of projection neurons, i.e., the lateral olfactory tract, the subcortical white matter, and the anterior commissure, showed reduced fresh volumes (Table 1) and myelin in *Npc1*^−/−^ mice (Figures 11A, 12B,D,F). Remarkably, these fiber tracts originate from perikarya located in areas that themselves show a strong reduction in volume. The lateral olfactory tract and the anterior commissure are associated with the olfactory system. The lateral olfactory tract consists of axons originating in the accessory olfactory bulb and of mitral/tufted axons from the olfactory bulb projecting to olfactory centers (Inaki et al., 2004). In rats, the anterior commissure links the rostroventral parts of the two brain hemispheres, including the olfactory bulbs, the olfactory tubercles, the piriform cortices, the amygdalae, and the perirhinal and entorhinal cortices (Horel and Stelzner, 1981; Jouandet and Hartenstein, 1983). In mice, it consists of 500,000 to 600,000 axons, 20% of which are myelinated (Sturrock, 1987; Livy et al., 1997). Thus, the reduced volumes of the olfactory fiber tracts correspond to the reduced volumes of the accessory olfactory bulb (Table 1). The same holds true for the subcortical white matter and the corpus callosum. The corpus callosum is a large commissural system that mainly links homotopic cortical areas of the two cerebral hemispheres (Yorke and Caviness, 1975; White and Czeiger, 1991; Olavarria and Van Sluyters, 1995); about 30% of the about 1 million axons in the mouse are myelinated (Tomasch and Macmillan, 1957; Vincze et al., 2008). The subcortical white matter is composed of partly myelinated axons of cortical pyramidal neurons projecting locally or to disseminated other cortical areas, thalamus, caudate putamen, brainstem, and spinal cord (White and Keller, 1989), as well as thalamocortical and corticothalamic projections (Caviness and Frost, 1980; Jones, 1998). As seen from Table 1, the main areas of origin of the fibers composing the white matter are those also disproportionately affected in volume reduction, i.e., frontal, motor and sensory cortices.

Additionally, the reduced density of oligodendrocytes in white matter structures of *Npc1^−/−^* mice (Weintraub et al., 1987; Takikita et al., 2004; Yan et al., 2011, 2014; Yu and Lieberman, 2013; Bernardo et al., 2021; Kunkel et al., 2023) should contribute to reduced fresh volume. In *Npc1*^−/−^ mice, from early postnatal development onwards, defects of differentiation of post-mitotic pre-myelinating oligodendrocytes into mature myelinating oligodendrocytes have been described by various groups (Weintraub et al., 1987; Takikita et al., 2004; Miller and Auchus, 2011; Yan et al., 2011, 2014; Hedrich, 2012; Yu and Lieberman, 2013; Mundy et al., 2014; Bernardo et al., 2021; Kunkel et al., 2023). As also seen in the present study, the amount of hypomyelination varied between different parts of the brain (Payne and Hales, 2004; Miller, 2013; Cole et al., 2019). Although the hypomyelination is evident, its contribution to the significant volume reductions in the various white and gray matter brain structures cannot be realistically assessed. Curiously, and as yet not explainable, there is no obvious myelin reduction in the optic tract (Figures 12D,F).

### 3.3 Reduced fresh volumes represent one factor in functional disorders in *Npc1*^−/−^ mice

The spontaneous or forced behavior of a mouse is a complex interplay of various neurophysiological, molecular biological and neuroanatomical parameters, one of which is the fresh volume of the whole brain or specific brain structures. Many studies are being conducted to determine relationships between the volume of parts of the brain and behavior in humans and animals (Francx et al., 2016; Guma et al., 2017; Chen et al., 2024; Gong et al., 2024; Magalhaes et al., 2024). Here, we showed increased fresh volumes of specific brain areas of *Npc1*^−/−^ mice treated with various drugs that, at least in part, showed related improvements in behavioral tests. One should keep in mind, however, that the fresh volume of a brain area represents the sum of the number and size of neurons and glial cells and the amount of neuropil including the degree of myelination.

### 3.4 Motor and sensory cortices in *Npc1*^+/+^ and *Npc1*^−/−^ mice

Locomotor and complex motor behavior and the motor defects in *Npc1*^−/−^ mice can be assigned to changes in motor and sensory areas. The Sham-treated mutant mice exhibited motor impairments in all performed tests, like open field, elevated plus maze and accelerod tests (Võikar et al., 2002; Hovakimyan et al., 2013a; Hung et al., 2016; Santiago-Mujica et al., 2019; Holzmann et al., 2021). The disproportionate reduction in fresh volumes in the motor and sensory areas as well as the reduced cerebellar volume including the Purkinje neuron loss (Maass et al., 2015) in *Npc1^−/−^* mice compared to *Npc1*^+/+^ mice could be the structural basis of these functional deficits. Given the greatly reduced interhemispheric projections passing through the small corpus callosum, inadequate motor coordination as seen in *Npc1*^−/−^ mice could be also expected.

#### 3.4.1 Frontal cortices in *Npc1*^+/+^ and *Npc1*^−/−^ mice

The frontal cortex is essential for decision-making, goal-directed behaviors, and emotional and cognitive processes (Graham et al., 2021; Bukwich et al., 2023). Data measured in open field tests were designed to reflect the abilities of spontaneous locomotion (total distance, total visits) and anxiety (central/total ratio). *Npc1*^−/−^ mice were more anxious than *Npc1^+/+^* mice (Holzmann et al., 2021) and cognitive impairment of *Npc1*^−/−^ mice was noticed in water maze tests (Võikar et al., 2002; Hovakimyan et al., 2013a). The obviously worse outcomes of these tests in *Npc1*^−/−^ mice may be due to reduced fresh volume and hypomyelination of respective brain areas (Figures 3A, 11A).

#### 3.4.2 Olfactory areas and olfaction in *Npc1*^+/+^ and *Npc1*^−/−^ mice

Impaired olfaction, known to occur in many neurodegenerative diseases, may also constitute a key symptom in Npc1. In previous studies, we found that the peripheral branch of the olfactory system, namely the olfactory mucosa, was severely damaged (Hovakimyan et al., 2013b). Olfactory receptor neuron numbers were reduced, proliferating neuroepithelial progenitor cells increased (Meyer et al., 2017), and olfactory receptor expression reduced (Meyer et al., 2018). We also demonstrated massive myelin-like inclusions in olfactory ensheathing cells (olfactory glia) as well as astrogliosis in the glomerular layer of the olfactory bulb, the first central relay of the central olfactory system (Hovakimyan et al., 2013b). The reduced olfactory bulb volume (Figures 6A,B) is difficult to evaluate, because the increased amount of GM3 (Figure 10) can be outweighed by decreased myelination as seen by the ST [ST (18:1/24:1)] (Figure 10). Myelination in the olfactory bulb begins in the outer plexiform layer and is most pronounced in the lateral olfactory tract, which constitutes only a small central portion of the olfactory bulb. The most significant differences between groups occur in olfactory-related structures containing reasonable amounts of myelin (Figures 12A,B). Both central parts (olfactory bulb, accessory olfactory bulb, and lateral olfactory tract) of the central olfactory system have significantly reduced volumes, which may be due, among other factors, to hypomyelination. Thus, this also may be a key factor in impaired olfactory acuity of *Npc1*^−/−^ mice, as evaluated in behavior tests (Meyer et al., 2018).

#### 3.4.3 Therapeutic interventions affected brain regions and areas of *Npc1*^+/+^ and *Npc1*^−/−^ mice differently

Relative changes of fresh volumes and heights of brain regions and areas of *Npc1*^+/+^ and *Npc1*^−/−^ in the Sham groups revealed a mean reduction of 24.38% in *Npc1*^−/−^ mice compared with the respective *Npc1*^+/+^ mice (Figure 11A). Looking at the various drugs, the most beneficial effect was seen following MIGLU treatment with a mean relative difference of 7.02% (Figures 11B,D).

**Figure 13:**
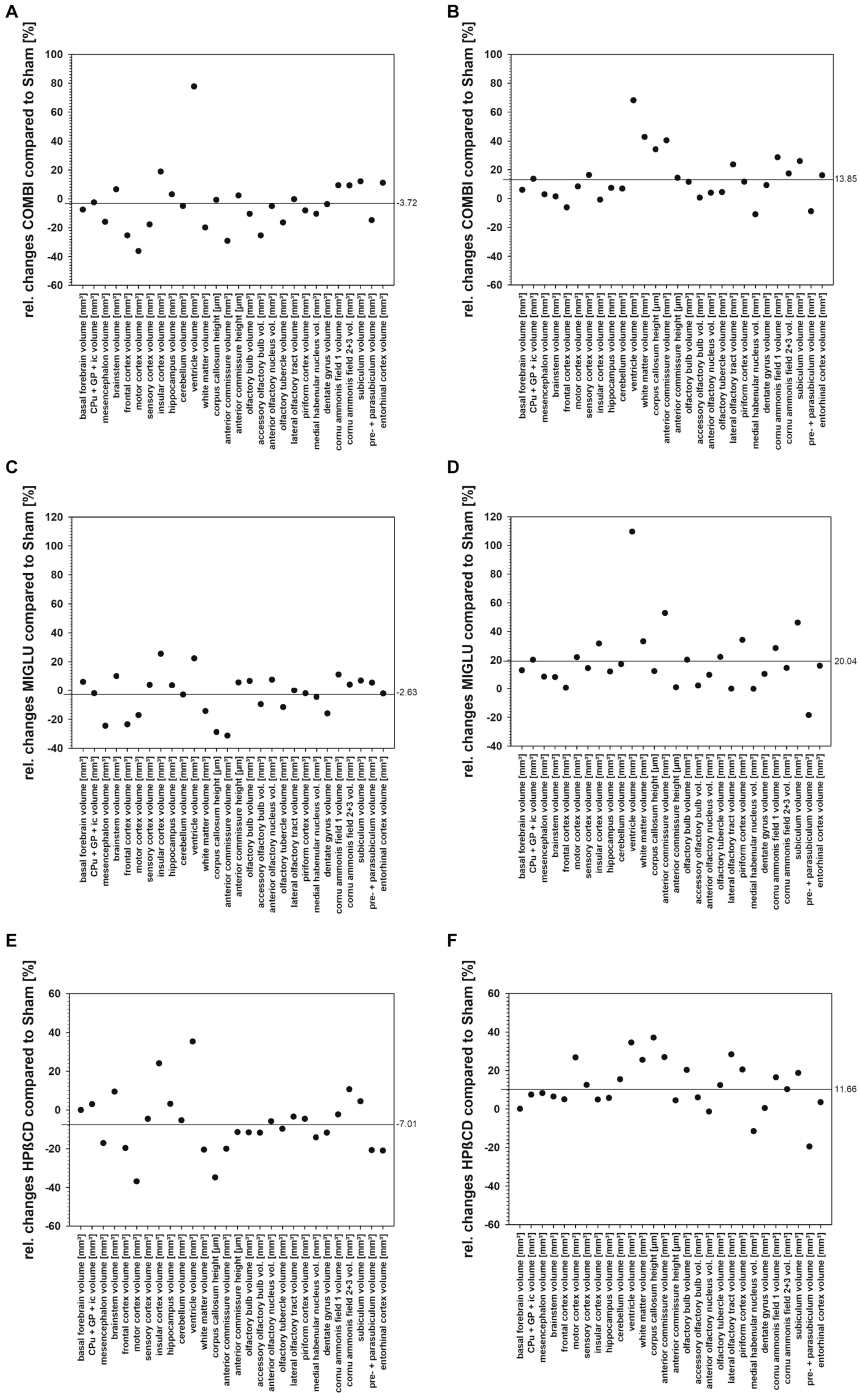
**(A–D)** Relative changes of fresh volume and heights of brain regions and areas of *Npc1*^+/+^ and *Npc1*^−/−^ mice of the COMBI, MIGLU, and HPßCD treatment groups compared with their respective Sham treatment group, calculated as **(A)** (*Npc1*^+/+^ COMBI minus *Npc1*^+/+^ Sham)/*Npc1*^+/+^ Sham, **(B)** (*Npc1*^−/−^ COMBI minus *Npc1*^−/−^ Sham)/*Npc1*^−/−^ Sham, **(C)** (*Npc1*^+/+^ MIGLU minus *Npc1*^+/+^ Sham)/*Npc1*^+/+^ Sham, **(D)** (*Npc1*^−/−^ MIGLU minus *Npc1*^−/−^ Sham)/*Npc1*^−/−^ Sham, **(E)** (*Npc1*^+/+^ HPßCD minus *Npc1*^+/+^ Sham)/*Npc1*^+/+^ Sham, **(F)** (*Npc1*^−/−^ HPßCD minus *Npc1*^−/−^ Sham)/*Npc1*^−/−^ Sham.

Relative changes of fresh volumes and heights of brain regions and areas of *Npc1*^+/+^ and *Npc1*^−/−^ mice of the COMBI, MIGLU, and HPßCD treatment groups compared to their respective Sham treatment group differed remarkably. Differences were noted between the same treatments administered to *Npc1*^+/+^ and *Npc1*^−/−^ mice. Whereas COMBI treatment in *Npc1*^+/+^ mice reduced these parameters by 3.72% (Figure 13A), this treatment in *Npc1*^−/−^ mice increased fresh volume and heights by 13.85% (Figure 13B). More differentiated results were found in MIGLU treatment groups. Whereas MIGLU treatment in *Npc1*^+/+^ mice only minimally reduced these parameters by 2.63% (Figure 13C), MIGLU treatment in *Npc1*^−/−^ mice significantly increased fresh volume and heights by 20.04% (Figure 13D). Compared with Sham-treated *Npc1^+/+^* mice, HPßCD treatment reduced these parameters by 7.01% (Figure 13E); however, HPßCD treatment in *Npc1*^−/−^ mice significantly increased the mean value by 11.66% (Figure 13F). In line with the present findings, a positive effect of single HPßCD injection at P7 *Npc1*^−/−^ mice was also seen by Nusca et al. (2014) at P28 as a partly rescue of normally occurring cerebellum size reduction. The same group reported a positive effect of HPßCD in *Npc1^+/+^* mice, injected at P4, P7, and thereafter weekly up to P49 and investigated at P75, a partly recue of myelinated fibers in the primary visual cortex and optic nerve compared with sham-treated *Npc1^+/+^* mice. Myelin rescue was accompanied by a normalization of cellularity and cytoarchitecture (Palladino et al., 2015). It is controversial whether HPBCD effectively crosses the blood–brain barrier when applied outside the CNS. Several studies in mice (e.g., Vecsernyés et al., 2014; Calias, 2017) or cats (Ward et al., 2010) reported on reduced HPßCD concentrations in the CNS due to its restricted passage through the blood–brain barrier. Effective CNS concentrations can be achieved either by intrathecal administration, or by high intravenously or intraperitoneally applied doses (>4.000 mg/kg), which in turn, can lead to serious side effects such as hearing loss (Davidson et al., 2009; Ward et al., 2010). In addition, a single dose of intraperitoneally administered HPßCD (4.000 mg/kg) has been reported to lead to enhanced development of oligodendrocytes in mice, the loss of which is responsible for demyelination in Npc1 (Kunkel et al., 2023). In humans, a phase 1–2 trial including a very limited number of NPC1 patients with intrathecally administered HPßCD showed a slowed disease progress (Ory et al., 2017). More recently, a phase 1-trial suggests that even intravenously applied HPßCD (1,500 mg/kg or 2,500 mg/kg HPβCD i.v., every 2 weeks) leads to a pharmacological effect in the CNS, even at low concentrations in the cerebrospinal fluid (Hastings et al., 2022). Taking all results together, MIGLU treatment had the smallest negative influence on fresh volume and heights of brain regions and areas of *Npc1*^+/+^, whereas MIGLU was the therapeutically most beneficial of all tested drugs on fresh volume and heights of brain regions and areas in *Npc1*^−/−^ mice. When all structures were examined, the HPßCD treatment had the most negative effect in *Npc1*^+/+^, whereas it was the therapeutically least beneficial of all tested drugs on fresh volume and heights of brain regions and areas in *Npc1*^−/−^ mice. The COMBI treatment displayed effects in between those of the MIGLU and HPßCD treatments. Thus, it can be speculated that the outcome of COMBI treatment is a combination of the more positive effects of MIGLU and the less positive effects of HPßCD treatment.

#### 3.4.4 The drug-induced increase in fresh volume in *Npc1*^−/−^ mice and functional benefits are partially related

A therapeutic approach using the substrate-reduction therapy (MIGLU) and/or the byproduct therapy (HPßCD) has been shown to ameliorate the disease course in *Npc1*^−/−^ mice (Davidson et al., 2009; Hovakimyan et al., 2013a) and humans (Lachmann et al., 2004; Patterson et al., 2007; Pineda et al., 2009; Brand et al., 2015; Santos-Lozano et al., 2015), explaining the benefit by the interference of the drugs with the lysosomal cholesterol traffic that is altered by the loss of function of the mutated Npc1 protein (Xie et al., 1999; Zervas et al., 2001; Aqul et al., 2011). However, literature data and own results reveal that the treatment-related benefits on different behaviors vary considerably (Supplementary Table S2).

#### 3.4.5 Drug effects on motor system components in *Npc1*^+/+^ and *Npc1*^−/−^ mice

*Npc1*^+/+^ mice: As far as has been studied, no effects of COMBI and MIGLU treatment of *Npc1^+/+^* mice on motor performance can be seen in the accelerod test, walking and swimming speeds in the elevated plus maze test, the open field test and the water maze test (Schlegel et al., 2016).

*Npc1*^*−*/−^ mice: As already mentioned by Hung et al. (2016) and Santiago-Mujica et al. (2019), the distance traveled on the rotarod by *Npc1*^−/−^ mice was significantly less than by *Npc1^+/+^ mice.* Concerning drug-induced increase of fresh volume, especially in female Npc1^−/−^ mice, COMBI, MIGLU, and HPßCD treatment positively influenced spontaneous locomotor abilities and coordination in mutant mice: the total distance and number of visits significantly increased, and accelerod test performance improved with respect to the maximum speed reached (Holzmann et al., 2021). These significant improvements in spontaneous locomotor abilities in COMBI-treated Npc1^−/−^ mice may, at least in part, be the functional consequence of the drug-induced absolute (Figure 5A) and relative (Figure 9B) increases in white matter volumes, although the respective cortices that form the origin of the callosal fibers did not change their volumes (Figures 3B, 13B). Interestingly, the volume of CPu + GP + ic in MIGLU-treated Npc1^−/−^ mice significantly exceeded the volume of Sham-treated Npc1^−/−^ mice (Figure 4B). In line with the drug-induced motor improvement was also less cerebellar neurodegeneration after COMBI therapy in Npc1^−/−^ mice (Maass et al., 2015).

#### 3.4.6 Drug effects on frontal cortices in *Npc1*^+/+^ and *Npc1*^−/−^ mice

*Npc1*^+/+^ mice: Total walking distance and relative center distance in the open field test are linked to explorative locomotor activity and anxiety-related behavior. Both COMBI- and MIGLU-treated wild type mice showed enhanced anxiety-related behavior in the elevated plus maze compared to Sham-treated wild type mice (Schlegel et al., 2016). The reduced fresh volumes in the frontal cortices of the respective Npc1^+/+^ mice could apparently be related to these drug-induced behavioral changes.

*Npc1^−/−^ mice:* In *Npc1^−/−^* mice, neither treatment resulted in significant changes in anxiety-related behavior, as evidenced by only non-significant differences in relative center times in the open field tests (Holzmann et al., 2021). This non-improvement of the various therapies in *Npc1^−/−^* mice are apparently consistent with the unaffected fresh volumes in the hippocampal region (Figure 3E) and the frontal cortices (Figures 3A, 13B,D,F).

#### 3.4.7 Drug effects on hippocampal areas in *Npc1*^+/+^ and *Npc1*^−/−^ mice

*Npc1*^+/+^ mice: The fresh volumes of hippocampal subareas were not changed by either drug treatment (Figures 3E, 7A–F). As seen in the water maze test, combination-treated mice performed just as well as Sham-treated wild-types, whereas mice treated with MIGLU displayed impaired spatial learning (Schlegel et al., 2016).

*Npc1*^*−*/−^ mice: Compared to the results in the water maze test of Sham-treated *Npc1*^+/+^ mice, the Sham-treated mutants exhibited impairment in remembering the location of the hidden platform. The spatial learning of the mutants, however, did not benefit from drug therapy (Hovakimyan et al., 2013a). Related to this result, the volume of the hippocampal regions was unaltered by COMBI, MIGLU and HPßCD therapy (Figure 3E), although some sub-regions showed increased volume in COMBI- and/or MIGLU-treated *Npc1*^*−*/−^ mice (Figures 7C–E).

#### 3.4.8 Drug effects in olfactory areas and olfaction in *Npc1*^+/+^ and *Npc1*^−/−^ mice

Measurements of olfactory-relevant structures in the brain have been carried out, as an impaired sense of smell can be a very early sign of neurodegeneration; this has been demonstrated for Parkinson’s and Alzheimer’s disease (Doty et al., 1987; Hawkes et al., 2009). However, olfactory impairment in NPC1 has not yet been reported in humans. The animal model described in this study shows morphological evidence for dysosmia as seen mainly by a reduced volume of olfactory-related structures in *Npc1*^*−*/−^ mice (see Table 1). In a behavioral buried pellet test, *Npc1* mutant mice exposed a significant olfactory deterioration, compared to wild type controls (Meyer et al., 2018).

When *Npc1*^−/−^ mice were treated with COMBI or HPßCD, we observed an improvement in olfactory performance in the behavioral test and at the same time morphologically a significant reduction in astrogliosis and microgliosis in the olfactory bulb. Also, the *BAX/Bcl2* ratio, an indicator of susceptibility to apoptosis in the olfactory bulb, could be significantly reduced upon treatment, most effectively with HPßCD (Meyer et al., 2018). Depletion of sulfatide lipid [ST (18:1/24:1)] (Figure 10), involved in myelin-synthesis, in the internal plexiform layer of the olfactory bulb and the beginning lateral olfactory tract as seen in MSLI may also contribute to *Npc1*-related olfactory impairment in mice (Meyer et al., 2017). Similar myelination effects of the lateral olfactory tract were reported in Kallmann syndrome in humans (Garcia-Gonzalez et al., 2013). We were able to show a significant reduction in the volumes of the olfactory areas in the *Npc1*^−/−^ animals compared with the measured values of healthy animals, with particular emphasis on the olfactory bulb and the aforementioned pathways.

Generally, dysosmia can also be a result of peripheral events, namely the reduction of olfactory sensory neurons that reach the olfactory bulb only at a severely limited number, as reported in previous reports of our group (Hovakimyan et al., 2013b; Meyer et al., 2018; Witt et al., 2018). However, the number of tyrosine hydroxylase- expressing interneurons in the olfactory bulb did not change in mutant mice compared to wild type animals (Meyer et al., 2018), and mRNA levels for olfactory marker protein and distinct olfactory receptors remained unchanged speaking mainly for a central reason for olfactory impairment. Therefore, we believe that olfactory dysfunction in *Npc1* mice is due to a central transduction problem of sensory inputs that could be represented by the highly reduced myelination levels in tertiary olfactory structures, e.g., the anterior commissure (Table 1).

Monotherapy with HPßCD had positive effects on body and brain weight as well as on the volume reduction of olfactory areas compared with the Sham therapy. The COMBI therapy was also able to compensate for the loss of body weight and the loss of volume in some olfactory areas. Volume reductions in Sham-treated *Npc1^−/−^* mice were most prominent in structures that relate to high myelinization (anterior commissure, lateral olfactory tract), but were also significant in the piriform cortex and olfactory bulb (Table 1; Figure 11A). However, similar volume changes were also recognizable in the habenula nuclei and the diameters of the commissural pathways anterior commissure and corpus callosum, so that it is not possible to speak of a specifically increased involvement of olfactory structures. In summary, we may conclude that the *Npc1* mutations exert a relevant, but not specific, influence on the volumes of olfactory areas. This leads to a reduction in volume, which can be best ameliorated, but not normalized, by the administration of HPßCD or MIGLU in monotherapy.

## 4 Conclusion and future research directions

The present results show the fresh volumes of brain regions of whole brains and selected areas of Sham-treated *Npc1^+/+^* and *Npc1*^−/−^ mice as well as data after COMBI, MIGLU and HPßCD treatments of both *Npc1^+/+^* and *Npc1*^−/−^ mice. As there are no causal treatment options, only symptomatic treatments are currently available. Combination therapy with miglustat, HPßCD and allopregnanolone has largely reduced the development of neurological symptoms (Davidson et al., 2009; Meyer et al., 2017, 2018) and also improved visceral symptoms such as hepato- and splenomegaly, but increased both lipolysis and cholesterol transport via abca1 and apoE (Ebner et al., 2018). Side effects of high-dose HPßCD, which lead to degeneration of outer hair cells (Ward et al., 2010; Crumling et al., 2012), must also be taken into account. Since drug-induced changes in the fresh volume of certain brain regions or areas in *Npc1^−/−^* mice could not be clearly linked to behavioral changes, further parameters such as the lipid and protein and receptor composition of the brain need to be investigated in the future. In particular, the densities of different lipids are altered differently before and after the non-functional Npc1 protein. In future studies, we will investigate different parts of the brain of *Npc1^+/+^* and *Npc1^−/−^* mice by mass spectrometric lipid imaging of representatives of, e.g., STs, gangliosides, glycerolipids, glycerophospholipids, phosphatidic acids and phosphatidylcholines.

## 5 Materials and methods

### 5.1 Animals

All animal procedures were approved by the local authorities (Landesamt für Landwirtschaft, Lebensmittelsicherheit und Fischerei des Landes Mecklenburg-Vorpommern; approval ID: 7221.3–1.1-030/12, 14 June 2012). All institutional guidelines for animal welfare and experimental conduct were followed, and all efforts were made to minimize suffering.

Heterozygous *Npc1*^*+*/−^ mice breeding pairs of Npc1 mice (BALB/cNctr-*Npc1*^m1N^/−J) were obtained from Jackson Laboratories (Bar Harbor, ME, United States) for generating homozygous *Npc1*^−/−^ mutants and *Npc1*^+/+^ control wild type mice. Mice were maintained under standard conditions with free access to food and water with a 12 h day/night cycle, a temperature of 22°C, and a relative humidity of about 60%. Genotypes were determined until postnatal day P7 by PCR analysis of tail DNA (Hovakimyan et al., 2013b; Witt et al., 2018). *Npc1*^−/−^ mutants and *Npc1*^+/+^ wild type controls of both sexes were used for different therapeutic treatment schedules.

Altogether, 22 wild type and 25 mutant mice of both sexes were involved in this study. The exact numbers of animals investigated in the various groups were: Sham *Npc1*^+/+^ 8; COMBI *Npc1*^+/+^ 6; MIGLU *Npc1*^+/+^ 1; HPßCD *Npc1*^+/+^ 7; Sham *Npc1*^−/−^ 9; COMBI *Npc1*^−/−^ 6; MIGLU *Npc1*^−/−^ 4; HPßCD *Npc1*^−/−^ 6. Brains of both genders were evaluated as there are no significant gender-specific differences in the brain weight (mean ± SD) of sham-treated mice *Npc1*^+/+^ mice (males 0.467 ± 0.015 vs. females 0.463 ± 0.009, *p* = 0.478) nor in the respective *Npc1*^−/−^ mice (males 0.387 ± 0.012 vs. females 0.394 ± 0.016 ±, *p* = 0.181) (data from Holzmann et al., 2021).

### 5.2 Treatment

**Figure 14:**
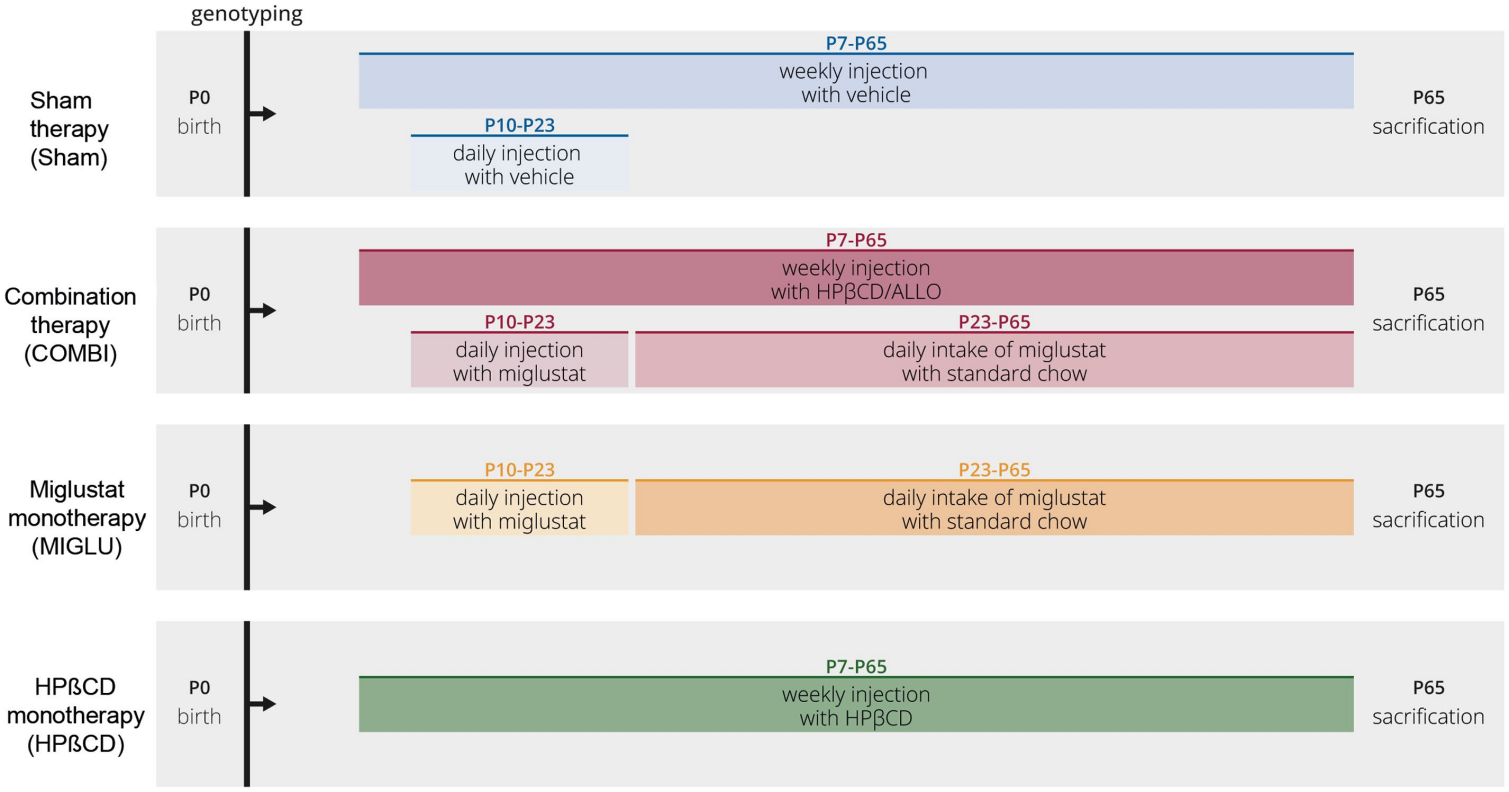
Timeline of drug administrations for all experimental groups. Mice of the Sham groups were injected with the respective amounts of 0.9% NaCl according to the treatment plan of the combination-treated group. At P7 and thenceforth, NPC1 mice were injected weekly with allopregnanolone (25 mg/kg; Sigma Aldrich, St. Louis, MO) dissolved in HPßCD (4,000 mg/kg, i. p.; Sigma Aldrich). At P10 and until P23, animals were injected daily with miglustat (300 mg/kg, i. p.; Zavesca; Actelion Pharmaceuticals, San Francisco, CA). From P23 onward, animals were fed with miglustat included in standard chow (1,200 mg/kg per day) until termination.

We used 4 treatment groups of *Npc1*^+/+^ and *NPC1^−/−^* mice (Figure 14). Combination therapy (COMBI group): Starting at postnatal day 7 (P7) and weekly thereafter, mice were injected with HPßCD/ALLO (25 mg/kg ALLO dissolved in 40% HPßCD, i.p.) (both from Sigma-Aldrich, Munich, Germany). Additionally, from P10 until P23 mice were injected daily with MIGLU (300 mg/kg, i. p.; Zavesca; Actelion Pharmaceuticals, San Francisco, CA), dissolved in saline. Starting at P23 and until termination of the experiment mice were fed standard chow including MIGLU with a daily dose of 1,200 mg/kg. HPßCD monotherapy (HPßCD group): HPßCD was injected starting at postnatal day 7 (P7) and weekly thereafter, in the same amount as included in COMBI (4,000 mg/kg, i. p.; Sigma Aldrich, Munich, Germany). MIGLU monotherapy (MIGLU group): comparable with COMBI, mice were injected daily with MIGLU (300 mg/kg, i. p.) at P10 until P23. From P23 onward, animals were fed standard chow including MIGLU with a daily dose of 1,200 mg/kg. Sham-treated mice (Sham group) were injected following the scheme of the COMBI mice, however, omitting the drugs in the saline. All mice were sacrificed at P65.

### 5.3 Staining procedures

After sacrifice, animals were perfused transcardially via the left ventricle with 15 mL of with 0.9% sodium chloride, followed by 50 mL of Bodian’s fixans, the solution consisting of 900 mL of 80% isopropanol, 50 mL of 37% formaldehyde and 50 mL of glacial acetic acid (Zilles, 1985; Mulisch and Welsch, 2015). Following dissection, brains were weighted and postfixed in the same fixans for 2 days, and subsequently embedded in paraffin wax using standard procedure (Zilles, 1985). Paraffin-embedded specimens, with a section thickness of 20 μm, were used for the Nissl staining (Zilles, 1985). Myelin staining according to Gallyas (1979) was performed on unfixed cryosections, with a section thickness of 20 μm.

### 5.4 Determination of regional fresh volumes

Fresh volumes of specific brain regions were calculated from the volumes determined from paraffin embedded brain slices. Although calculating volumes directly from sections of fixed brain could give reasonable results, we calculated fresh volumes of brain structures using individual brain-specific shrinkage correction. Shrinkage corrections lead to more accurate measured values. We found that working with the uncorrected “raw values” from fixed tissue led to slightly worse *p*-values in the post-hoc analyses. The delineations of cortical area and brain stem nuclei were based on the criteria given by Wree et al. (1981) and Franklin and Paxinos (2008). The delineations of the 13 brain differentiated regions are illustrated in Supplementary Figures S1–S22. For each structure, the outermost rostral and caudal borders were taken as landmarks. Approximately 10 equidistant sections of the structure of interest located between the landmarks were evaluated (Zilles et al., 1982; Beck et al., 1993). The outlines of the brain areas and layers were traced on paper with the aid of a drawing tube (magnification 15x or 64x), and the area of each structure was determined planimetrically using an image analysis system. For interindividual comparisons it is essential to have a parameter that is independent of the unavoidable shrinkage due to histological procedures. The fresh volume of a brain region was estimated using the Cavalieri method (Gundersen and Jensen, 1987), taking into account the shrinkage factor (for the method, see Wingert, 1969; Kretschmann and Wingert, 1971; Zilles, 1978; Wree et al., 1981). By definition, the shrinkage factor, i.e., the volume of the whole brain after sectioning divided by the fresh volume of the whole brain had to be determined for each individual brain separately.

Since other investigations (Kretschmann et al., 1975) and our own unpublished results have shown that the weight and the specific weight of fixed brains do not differ from the weight of fresh brains, the fresh volume (FV) of the whole brain can be easily calculated by weighing the fixed brains and multiplying the brain weight by its specific weight (1.033 g/mL). This value for freshly dissected brains was published by Johansson and Linder (1982) for the rat, by Wingert (1969) for the mouse, and by Zilles (1978) for tupaia, and for fixed human brain by Kretschmann et al. (1982). After sectioning the brain, the total volume of the sectioned brain (SV) has to be calculated from the thickness of the sections (ST), the number of planimetered sections (N), the number of sections between two planimetered sections (n) and the area A of the section i according to the following Equation (1):

(1) $$SV=\left(n+1\right)\;x\;ST\;x\overset{N}{\underset{i=1}{\sum }}\mathrm{Ai}$$

The shrinkage factor (SF) is SV divided by FV (SF = SV/FV). Basically, the determination of the fresh volume of a given brain structure follows the same mathematical procedure as given above. The fresh volume of a structure is Equation (2):

(2) $$FV=\left(n+1\right)\;x\;ST\;x\overset{N}{\underset{i=1}{\sum }}\frac{\mathrm{Ai}}{\mathrm{magnificatio}n^{2}\;x\;SF}$$

### 5.5 Data analysis

The results are presented as means ± SEM. In all cases, *p* ≤ 0.05 were considered significant. All data were subjected to three- or two-way ANOVA. The Holm-Sidak approach was used for *post hoc* comparisons. All statistical analyses were done using SigmaPlot 14 Software (Systat Software, Inc., San Jose, CA 95110, United States).

### 5.6 Mass spectrometric lipid imaging

Mass spectrometric lipid imaging of mouse brain specimens was performed according to Gonzalez de San Roman et al. (2018). Frozen and dried 10 μm sections were coated with matrix (20 mg/mL 2, 5-dihidroxybenzoic acid in 50% ethanol) and analyzed using high-mass resolution MALDI-MS in positive and negative ion mode at 100 or 30 μm lateral resolution.

According to O’Donnell et al. (2020) we have referred to all the points such as resolution, ionization mode, matrix and others for mass spectrometric imaging. First, raw data acquired with a MALDI-LTQ-Orbitrap XL instrument (Thermo) were converted into the public imzML format suitable for SCiLS lab 2018b (Bruker, Bremen, Germany) and normalized using the total ion count (TIC). The regions of interest (ROI) were selected based on the Nissl staining. A receiver-operator characteristic (ROC) analysis was performed for each ROI to determine significantly increased or decreased m/z values in *Npc1^−/−^* animals compared with wild types. The ROC analysis results were then evaluated to find m/z values that were consistently significantly increased or decreased in each ROI across all seven datasets. Distribution maps of these selected m/z values were generated using the SCiLS lab MVS software package with edge-preserving weak image denoising. Distribution fingerprint plots were created with Excel. Lipid species were assigned by a comparison of the measured molecular masses to the Lipid MAPS database,^1^ the Madison Metabolomics database (Metabolomics Society: Databases)^2^ and previous reports (Berry et al., 2011; Fernandez et al., 2016). For assignment, a maximum of 5 ppm deviation between measured and theoretical mass was selected as the tolerance window.

## Data availability statement

The raw data supporting the conclusions of this article will be made available by the authors, without undue reservation.

## Ethics statement

The animal study was approved by Landesamt für Landwirtschaft, Lebensmittelsicherheit und Fischerei des Landes Mecklenburg-Vorpommern; approval ID: 7221.3–1.1-030/12, 14 June 2012. The study was conducted in accordance with the local legislation and institutional requirements.

## Author contributions

VA: Conceptualization, Investigation, Methodology, Writing – original draft, Writing – review & editing. DH: Investigation, Writing – review & editing. KS: Investigation, Writing – review & editing. JS: Investigation, Writing – review & editing. OS: Formal analysis, Investigation, Methodology, Software, Writing – review & editing. CH: Formal analysis, Investigation, Methodology, Software, Visualization, Writing – original draft, Writing – review & editing. AR: Funding acquisition, Resources, Writing – review & editing. H-JB: Investigation, Methodology, Visualization, Writing – original draft, Writing – review & editing. EG: Investigation, Methodology, Resources, Software, Writing – review & editing. PH: Investigation, Methodology, Software, Writing – review & editing. KA: Funding acquisition, Investigation, Methodology, Resources, Writing – review & editing. JK: Visualization, Writing – original draft, Writing – review & editing. NH: Resources, Writing – review & editing. MW: Conceptualization, Investigation, Methodology, Supervision, Validation, Visualization, Writing – original draft, Writing – review & editing. AW: Conceptualization, Funding acquisition, Investigation, Methodology, Project administration, Resources, Supervision, Validation, Writing – original draft, Writing – review & editing.

## Footnotes

^1^http://www.lipidmaps.org/

^2^internationalmetabolomicssociety.org
