# Supplementary material for: Differently increased volumes of multiple brain areas in Npc1 mutant mice following various drug treatments
Source: Front Neuroanat. 2024 Jul 16;18:1430790. doi: 10.3389/fnana.2024.1430790 (PMC11286580; doi:10.3389/fnana.2024.1430790)
Supplement: Supplementary file 1 [file Data_Sheet_1.ZIP › Supplementary Table 2.docx]

**Supplementary Table 2.** Overview of results of behavioural tests of *Npc1* mice treated with various drugs.

| **Publication** | **Mice Strain, gender** | **Treatment** | **Behaviors** | **Results** |
| --- | --- | --- | --- | --- |
| (Võikar et al., 2002) | Heterozygous Balb/C mice carrying the mutation in the NPC1 protein, kindly provided by the Pentchev  laboratory at NIH. | A metabolic and neurological disorder reminiscent of human NPC disease has been described in Balb/C mice, and it was recently shown that the mutation in the NPC mice resides in the orthologous murine *Npc*1 gene. Here we have followed the growth rate and applied behavioural methods in order to establish the onset and development of the major symptoms in the NPC mouse model. | Mice were subjected to a battery of coordination tests at the age of 28, 35, 42, 49 and 56 days. The battery consisted of four consecutive tests (vertical screen, beam balancing, coat hanger, rota-rod) in one session and each mouse carried out two sessions on the same day at the respective age.  1. Vertical screen (VS).  2. Balancing on a round beam (BB).  3. Coat hanger (CH).  4. Rota-rod (RR).  5. Spontaneous locomotor activity. At the age of 42 days the locomotor activity of the mice was assessed. Videoanalyser (Columbus Instruments, OH USA) registered the distance travelled by the animals  in the open field arena (30×30×15 cm rectangular arena with transparent walls under reduced illumination, _100 lx×) every 3 min during 15 min.  6. Learning and memory in Morris water maze. | The battery of coordination tests (vertical screen, beam balancing, coat hanger and rotating rod) established motor impairment of the NPC mice already at the age of 28–42 days, well before the onset of visually detectable ataxia. Decreased exploratory activity and lack of habituation was revealed in the NPC mice by open field test. The diseased mice were unable to learn and remember the location of the hidden escape platform in spatial water maze task suggesting cognitive impairment. In several tests the male NPC mice were more affected than the females. The present study represents the first behavioral analysis of the NPC mice. The battery of behavioral tests employed here should be valuable in the assessment of effective approaches to treat NPC, for which no preventive or curative measures have so far been established.  *Rota*-*rod*  The ability to walk on the rotating rod decreased very rapidly in the diseased mice during the observation period and again, the NPC males were more affected [Fig. 6; genotype *F*(1,50)=188.8, *P<*0.01; sex *F*(1,50)=5.4, *P*_0.05; age *F*(4,200)=53.7, *P<*0.01; genotype×sex *F*(1,50)=6.2, *P<*0.05]. The difference between the WT and NPC mice was first observed at 35 days in the male and at 42 days in the female group. |
| (Bascuñan-Castillo et al., 2004) | *Npc1*^NIH^ mutant mice from the BALB/cJ background were maintained by brother-sister mating of heterozygous animals. | The *Npc1-/-* mice were weaned, genotyped and separated according to sex when they were approximately 21 days old. Treatment was initiated at this time. The mice were divided into four groups: control, tamoxifen treated, vitamin E treated, and tamoxifen plus vitamin E treated.  Mice receiving tamoxifen were injected intraperitoneally with the drug suspended in peanut oil on a weekly basis with a dosage delivering 0.023 µg/g/day (0.01 c.c./g. and assuming linear release). Mice receiving vitamin E were given 70 µl (25 IU) of D-alpha tocopherol through oral administration via a pipette on a weekly basis. | Each mouse was evaluated weekly on a Rota-Rod test instrument (Ugo Basile, NY, USA). The Rota-Rod was driven at a constant rate of 25 rpm. The mouse was given 3 trials on the Rota-Rod, and the maximum trial time was recorded. Mice that could not remain on the Rota-Rod for 10 seconds were considered to have failed. | Control mice showed no significant sex differences (58 days, 5661 days 95% CI). Among tamoxifen-treated mice, there was a slight delay before failure on the Rota-Rod (64 days, 5171 days 95% CI). Mice treated with vitamin E showed a 7-day improvement over controls, with a median failure age of 65 days (62–66 days, 95% CI). Mice treated with vitamin E were not significantly different from mice treated with tamoxifen. Mice given both treatments, showed an 11-day increase over controls with a median failure age of 69 days (5874 days, 95% CI;). In addition, these mice showed a significant increase in absolute Rota-Rod performance (time spent on rotating rod) in the early stages of disease progression. |
| (Griffin et al., 2004) | BALB/c NP-C mouse | Mice received one of three treatments: 0.5–2 μM ALLO in drinking water, with the concentration increasing by 0.5 μM every 2 weeks; a subcutaneous time-release pellet (250 mg over 90 d); or a single subcutaneous injection of 25 mg/kg of ALLO in 20% HPßCD (2-hydroxypropyl-β-cyclodextrin; Sigma), corresponding to 1.25 mg of ALLO per ml of 20% HPßCD. | Behavioral tests assessed locomotion, balance and coordination.  The open-field test graded locomotion and exploratory movements, incorporating an analysis of paw placement and position, limb movement, trunk position, stability and coordination of stepping, into the 21-point score.  Strength and motor coordination (10 points) were evaluated by the maximal angle of an inclined plane that the mouse could scale and the ‘tightrope test’, which assesses the mouse’s ability to stay on a 12-inch rope suspended between two poles (hang time, wrapping its tail, placing all four paws on the rope and traveling the length of the rope). | Adding allopregnanolone to the drinking water of weaned NP-C mice at P21–P23 increased the lifespan of NP-C mice from 67 to 80 d (*n* = 12 mice in each group; *P* < 0.05), and implanting 90-d release pellets containing 250 g of AALLO under the skin of NP-C mice at P21–P23 (*n* = 12 mice) had a similar effect (mean survival 82 d; *P* < 0.01 versus NP-C mice receiving placebo pellets . Locomotor function and motor coordination declined at 8 weeks in both untreated and allopregnanolone-treated NP-C mice, but the rate of decline was less in allopregnanolone-treated NP-C mice. There was no difference in survival, locomotor function or motor coordination between male and female mice receiving allopregnanolone treatment.  Earlier administration of ALLO gave better results. A single injection of allopregnanolone (25 mg per kg body weight) at P23, P17, P10 or P7 progressively increased survival and delayed loss  of locomotion, coordination and weight. Mice treated at P7 had a slight loss in motor coordination at 11 weeks and normal locomotion for 12 weeks. Mice treated at P10 also showed significant delays in the onset of tremors and ataxia as compared with untreated NP-C mice (*P* < 0.05), but these delays were not as great as those seen in mice treated at P7. There was no difference in survival, locomotor function or motor coordination between male and female mice receiving single injections of ALLO . |
| (Zhang et al., 2004) | A breeding pair of heterozygous npc1^m1N^ mice of the BALB/c^nih^ strain was obtained from the Jackson Laboratory (Bar Harbor, ME) and bred to generate homozygous mice for this study. | The inhibitors were infused intracerebroventricularly  for a 2-week period, initiated at a pathologically incipient stage.  Roscovitine, olomoucine, and iso-olomoucine, obtained from LC laboratories (Woburn, MA), were dissolved in 100% dimethyl sulfoxide (DMSO, Sigma, St. Louis, MI) and diluted with saline to the desired concentrations.Two-week infusions were performed with four different concentrations of roscovitine, 72, 144, 300, and 600 nmoles/day, and 4-week roscovitine infusion involved delivery of 200 nmoles/day. In initial studies, we found that roscovitine above 600 nmoles/day in wild-type mice caused tremor, malaise, and weight loss and had to be euthanized within 1 week. Therefore, doses higher than 600 nmoles/day were discontinued. Olomoucine and isoolomoucine were infused at 2.5 _moles/day. All controls received the vehicle solution containing the appropriate percentage of DMSO.  ALZET microosmotic pumps (Model 1002, DURECT, Cupertino, CA) were used to infuse cdk inhibitors as described previously. Two-week pumps filled with 100 µl inhibitor solution except for higher doses (600 nmoles/day roscovitine, and 2.5µmoles/day olomoucine and iso-olomoucine each), which were placed in a coiled tube connected with the pump, because the higher percentage of DMSO (75%) required to maintain solubility at these concentrations is not compatible with the osmotic pumps. Because the weight of npc mice does not meet the criteria for implantation of the larger 4-week pumps, all 4-week treatments were performed using 2-week pumps, with replacement of the empty pump with a fresh one at the 2-week midpoint. Following the infusion, treatment mice were euthanized and the brains were removed rapidly and divided mid-sagittally. One half was fixed and paraffin-embedded for immunohistochemistry, and the other half was frozen at-80°C for immunoblotting analysis. | Before infusion and at weekly intervals till the end of the treatment, mice were weighed and evaluated for limb motor activity using the coat hanger test.36 Before and every 4 days during the drug treatment, these mice were tested for motor activity and overall muscle strength. The animals were allowed to grab a metal coat hanger suspended suspended 20 cm above a flat surface, observed for 2 minutes, and the length of time the mouse remained on the hanger measured (hanging time). The ratio of the hanging time at the end of the treatment and before treatment served as a measure of the decline (control) or sustenance (cdk inhibitor). These analyses were performed blinded. | These combined results suggest that roscovitine inhibits the progression of motor dysfunction, and also the weight loss associated with NPC. |
| (Ahmad et al., 2005){Ahmad, 2005 #3036}{Ahmad, 2005 #3036}{Ahmad, 2005 #3036}{Ahmad, 2005 #3036}{Ahmad, 2005 #3036}{Ahmad, 2005 #3036}{Ahmad, 2005 #3036} | Npc1^NIH^ mutant (NPC1^–/–)^ mice on the BALB/cJ background  or a mixed BALB/cJ x FVB background with the mdr1a knockout.  mdr1a^–/–^ mice were the knockouts developed by Schinkelet al. (1994) and were obtained from the Jackson Laboratory (Bar Harbor, ME) and interbred with Npc1^–/–^ on the BALB/cJ background to produce double mutants. This combination ofmdr1a^–/–/^Npc1^–/–^has previously been shown to correct the sterility of Npc1^–/–^females (Erickson et al., 2002). | ALLO (Sigma, St. Louis, MO) was dissolved in a 20% solution of HPßCD in water at 1.25 mg/ml by brief sonication of the chilled solution and was injected at 25 mg/kg subcutaneously at day 7 and intraperitoneally at later times. A vehicle-alone control was not included, because extensive studies on HPßCD treatment of *NPC1^–/–^* mice showed no effect on neurological symptoms (Camargo et al., 2001). The *NPC1^–/–^* mice were tail tipped and genotyped at 21 days of age. *NPC1^–/–^* pups and their littermates had been injected on day 7 as described above. In the case of repeated injections, only the *NPC1^–/–^* mice were again injected on day 21 and at 2-week intervals thereafter until death. | Each mouse was evaluated weekly on a Rota-Rod testinstrument (Ugo-Basile, New York, NY). The Rota-Rod was driven at an accelerating rate, 11–25 rpm in 300 sec. The maxi-mal testing time was 300 sec. The mouse was given three trials on the Rota-Rod, and the maximal trial time was recorded. Mice that could not remain on the Rota-Rod for at least 10 sec were considered to have failed. | Rota-Rod performance was moderately enhanced until about 10 weeks in the day-7-treated mice but gradually decayed at older ages. The double-mutant Npc1^–/–^, mdr1a^–/–^s double-mutant mice single-injected mice were not significantly different from the Npc1^–/–^single-injected mice. |
| (Li et al., 2005) | Heterozygous NPC1 mice with a BALB/c background were mated. | This study defines the functional, biochemical, and molecular events that ensue as nerve cell death occurs.  In most studies, the animals were weaned at 3 weeks of age onto a basal rodent diet (No. 7001 Harlan Teklad, Madison WI) that contained 0.02% (wt/wt) cholesterol. In some studies, groups of mice were placed on the same diet that had been enriched with 1.0% cholesterol (wt/wt) from the time of weaning until the end of the experiments. | The accelerating rotarod apparatus (Rotamex 4/8,  Columbus Instruments, Columbus, OH) was used to measure  motor coordination. Each mouse was placed on the rotating rod at a starting speed of 4 rpm and this speed was gradually increased to 40 rpm by 10 minutes. Four trials were carried out each day with a rest period of 1 hour between the first 2 trials  and the last 2 trials. | To explore this issue, 4 groups of 16 mice each containing equal numbers of male and female animals were evaluated by quantifying neuromuscular coordination utilizing the rotarod apparatus. There was no significant effect of cholesterol feeding in the NPC1^+/+^ animals between 3 and 11 weeks of age. However, the NPC1^-/-^ animals began to show poorer coordination at 5 weeks of age, and the duration that they remained on the rotating rod rapidly declined thereafter reaching nearly 0 minutes at 11 weeks. Importantly, there was no effect of cholesterol feeding. |
| (Hallows et al., 2006) | Heterozygous BALB/cNctr-*Npc1mIN*/J (*npc*^_^*^/^*^_)^ mice were obtained from The Jackson Laboratory (Bar Harbor, ME) and maintained  in our colony. *npc*^_^*^/^*^_^ mice were crossed with *p35*_*/*_ mice having a  C57BL/6 background (Hallows et al., 2003) to obtain double heterozygous  (*npc*^_^*^/^*^_^*^,^ p35*^_^*^/^*^_^) mice in the mixed BALB/c/C57BL/6 background.  Double-heterozygous mice were mated to obtain *wt*, *p35*^_^*^/^*^_^*^,^ npc*^_^*^/^*^_^,  and *npc*^_^*^/^*^_^*^,^ p35*^_^*^/^*^_^ genotypes in the mixed background, and all comparisons  between these different genotypes were made using littermates.  It should be noted that the mixed background did not have an observable  effect on the basic phenotypic features of either *npc*^_^*^/^*^_^ or *p35*^_^*^/^*^_^ mice. | Our p35 null mice have been useful for examining the role of cdk5/p25 in other types of neurodegenerations. | Mice were weighed and motor ability was assessed twice weekly between 3 and 10 weeks of age. To assess motor ability, mice were allowed to grasp the bottom of a coat hanger suspended  18 cm above a flat surface and allowed to remain on the hanger for up to 2 min (hanging time). Their behavior on the hanger was also noted. Hanging time was multiplied by behavior to obtain a numeric value for motor ability. Values were averaged within genotypes at each time point (*n =* 13 *wt*, 14 *p35*^_^*^/^*^_^, 10 *npc*^_^*^/^*^_^, and 13 *npc*_*/*_*, p35*^_^*^/^*^_^). | To determine whether weight loss or motor defects were improved in *npc*^_^*^/^*^_^ mice lacking p35/p25, we weighed mice and  tested their motor ability twice weekly between the ages of 3 and 10 weeks of age. Both *wt* and *p35*^_^*^/^*^_^ mice continued to gain  weight over the course of testing, whereas *npc*^_^*^/^*^_^ and *npc*^_^*^/^*^_^*,*  *p35*^_^*^/^*^_^ mice lost weight beginning at ~6 weeks of age.  Motor ability held fairly constant in *wt* and *p35*_*/*_ mice. *npc*_*/*_  mice demonstrated a dramatic decline in motor ability beginning at~6 weeks of age, whereas *npc*^_^*^/^*^_^*, p35*^_^*^/^*^_^ mice demonstrated this decline at an earlier time of~5 weeks. Overall, these findings show no improvement in weight loss or motor ability in *npc*^_^*^/^*^_^ mice lacking p35/p25. |
| (Chen et al., 2007) | A breeding pair of BALB/c NPC1NIH mice were obtained from Jackson Laboratories (Bar Harbor, ME). These mice were bred to produce normal (NPC11/1), heterozygous (NPC11/2), and  homozygous affected (NPC12/2) mice. DNA was isolated  from the tail tips of each mouse and PCR was performed  at the NPC1 locus using primer pairs as previously described (Loftus et al., 1997). | Estradiol Treatment:  NPC12/2 mice received subcutaneous injections of 2 mg/kg or 20 mg/kg of 17b-estradiol in corn oil weekly  from P7; the concentrations of 17b-estradiol were 10 ng/ 10 lL or 100 ng/10 lL. | Rota-Rod Performance Test:  This test measures motor coordination and balance (Zausinger et al., 2000). The apparatus consisted of a rotating rod with a diameter of 3 cm and a no slippery surface. The apparatus was turned on at the minimum  speed, the mice were positioned on the rotating rod, and acceleration started immediately until 10 rpm was reached. Three testing sessions were performed at 5 min intervals. Results were expressed as the longest time  that a mouse remained on the treadmill. Mice were  tested every 5 days from P30. | Estradiol Treatment Improved Behavioral Deficiencies in NPC12/2 Mice:  The locomotor function and motor coordination of NPC12/2 mice, estimated by the rotarod test, were significantly improved by the treatment with 17b-estradiol. Tremors were first detected at 62.3 days in 17b-estradiol-treated mice, significantly delayed as compared with 51.2 days in untreated mice (P < 0.01, n 5 15). There was no difference in survival, locomotor function, or motor coordination between male and female mice receiving 17b-estradiol treatment (data not shown). Thus, 17b-estradiol treatment significantly improved behavioral deficiencies in NPC12/2 mice. |
| (Alvarez et al., 2008) | BALB/c mice carrying a heterozygous mutation in the *npc1*  gene were kindly donated by Dr. Peter Pentchev (U.S. National Institutes of Health, Bethesda, MD, USA) | Mice received daily intraperitoneal injections of imatinib mesylate (Gleevec; Novartis, Basel, Switzerland) (5 mg/kg in 0.9% NaCl) from postnatal day (p) 7 or 28. Control groups (wild-type and NPC) received daily intraperitoneal injections of 0.9% NaCl. | Locomotor coordination was evaluated weekly by two tests during imatinib treatment. In the hanging test, the mouse was placed to hang at the center of a horizontal bar (3 mm diameter; 35 mm long) with forepaws. The body position of the animal was observed for 30 s and scored as described in Voĩkar *et al.* (2002). In the beam test mice were placed at the end of a beam (100 cm long; 2.5 cm wide). Animals were trained to finish the task as quickly as possible. The number of falls during the test was counted. Footprint analysis was performed in imatinib-treated animals from p28 at 8 wk of age, as described previously (Karimi-Abdolrezaee et al. 2006). | Imatinib therapy improves locomotor function in NPC mice:  We evaluated coordination and locomotor function using two different tests at regular intervals throughout treatment. The imatinib-treated NPC mice exhibited a significant improvement in the hanging test, which evaluates coordination of the four paws and the tail. A delay in the onset of symptoms was observed in p28 and p7 imatinib-treated mice from 7 and 8 wk, respectively. In the beam test, which evaluates equilibrium and coordination, the imatinib-treated NPC mice showed fewer falls than untreated NPC mice. In this test, improvement was observed from 6 wk for both p7 and p28 imatinib-treated mice. We also evaluated the footprint pattern of treated animals from p28. In this test, imatinib-treated NPC mice showed a significant improvement compared with untreated NPC mice. Although we did not find a significant difference in locomotor function between NPC mice receiving imatinib treatment from p7 and p28, we observed, as was shown previously, an increase in survival of mice in the p7 imatinib-treated group. This increase in survival correlates with a tendency toward greater corporal weight. |
| (Borbon et al., 2012) | Npc1−/− mice were obtained through breeding heterozygous Npc1+/− mice which are maintained on the BALB/cJ background. At 15 days animals were genotyped by PCR as described by Loftus et al. (1997). | We tested a total of five diets consisting of three concentrations of curcumin and controls: 1)  Chromadex, 2% curcumin (Billerey-Larmonier et al., 2008) mixed in NIH 7013 chow (Harlan Teklad, Madison, WI); 2) Acros, 0.17% curcumin (Acros Organics, Fisher catalog #AC 1858–0100) mixed in 5P14 base chow (PMI Nutrition International, Brentwood, MO);  3) NIH 7013, chow alone; 4) Lipidated curcumin Verdure Sciences, 0.05%curcumin (at 20%  in a stearic acid/phosphatidyl choline mix; lot 024908G2711CLEG, Verdure Sciences,  Noblesville, IN) delivered at 0.25% in NIH 7013 chow and 5) Lipidated vehicle, (Verdure  lapidated control, containing stearic acid and phosphatidylcholine, Verdure Sciences, Noblesville, IN) at 0.25% in 5P14 chow. | In order to evaluate balance and motor coordination, mice were tested using two different tests, the balance beam and the coat hanger. | The results from the balance beam show that Npc1+/+, wild-type control mice and Npc1−/− mice on the 2% curcumin (Chromadex) diet performed quite similarly. This is important since mice might show improvements in motor behavior and/or memory and yet not have greatly increased survival. The time spent on the beam was not different (not shown). The Npc1−/− untreated and the Npc1−/− mice on the Acros diet were similar and had lower scores. On the other hand, the results from the coat hanger test show a decline in score (performance rating) over age for Npc1−/− mice untreated or treated with either diet, as opposed to a constant high score for Npc1+/+ control mice. The high dose (Chromadex) that improved balance beam performance, showed a slight, non-significant improvement by 9 weeks which again could be important for morbidity. |
| (Hovakimyan et al., 2013) | BALB/cNctr-Npc1m1N/-J | The COMBI-treated animals were injected with HPßCD/ALLO (25 mg/kg ALLO dissolved in 40% HPßCD, Sigma, Seelze, Germany) weekly, starting at postnatal day 7 (P7). These mice were injected with MIGLU dissolved in 0.9% NaCl solution, 300 mg/kg, between postnatal days P10 and P23. Starting at P23 the mice were fed powdered chow with daily addition of MIGLU (Actelion Pharmaceuticals, Allschwil, Switzerland). For the sham-treatment the HPßCD/ALLO and MIGLU injections were substituted. | Locomotor activity was assessed in open field, elevated plus maze and accelerod tests. For assessment of spatial learning and memory the Morris water maze test was conducted. | The sham-treated mutant mice exhibited motor impairments in all performed tests.  In the water maze the sham-treated mutants exhibited impairment in remembering the location of the hidden platform.  CYCLO/ALLO/miglustat treatment positively influenced motor dysfunction: total distance and number of visits significantly increased, and accelerod performance improved. The spatial learning, however, did not benefit from therapy.  Open field. Total distance travelled, total visits, and central/total ratio were measured and compared between groups. The data were designed to reflect the abilities of spontaneous locomotion (total distance, total visits) and anxiety (central/total ratio). When compared to the sham-treated control mice, the total distance and number of total visits were significantly lower in the sham-treated mutant mice (p<0.001) which was indicative of impaired motor activity. The combination-treated mutant mice group revealed an increase in both parameters. When compared to the sham-treated mutant mice, the combination-treated ones travelled longer distances (4615±1290 cm vs. 3195±1291 cm) in the whole open field and revealed a higher number of total visits (283±112 vs. 219±83, p=0.051).  Collectively, these data strongly suggest that combined CYCLO/ALLO/miglustat treatment resulted in significant improvements in spontaneous locomotor abilities in NPC1 mutant mice.  Accelerod test. The mice of all groups acquired the accelerod test quickly and reached a stable level of performance before testing (data not shown). No differences in the learning ability of mice of different groups could be observed. Therefore, the test performance is interpreted as a measure of motor coordination and balance (Karl et al., 2003). The maximum rotation speed achieved by each group, displayed in rounds per minute at postnatal days 35 and 60 in the accelerod test.  At postnatal day 35 the sham-treated controls and NPC1 mutants revealed no significant differences in maximum speed, both exhibiting 21 rounds per minute (p=0.912).  The combination-treated mutant mice, however, demonstrated an increase in maximum speed, reaching 34 rounds per minute, and this difference was statistically significant when compared to the shamtreated mutant group (p<0.001).  At time point P60 (Fig. 5B) the accelerod performance was significantly (p<0.001) disrupted in the sham-treated NPC1 mutant group (10±4 rounds per minute) when compared to the sham-treated control group (21±6 rounds per minute). The combination-treated mutant mice, however, demonstrated a significantly better performance when compared to the sham-treated ones, reaching 24±7 rounds per minute (p<0.001). |
| (Williams et al., 2014) | BALBc/NPC^nih^ | Ibuprofen (Sigma, 100 mg/kg/day) was supplemented as a dry admixture to powdered RM1 mouse chow (SDS, UK) (from 6 weeks of age, due to the toxicity seen with earlier dosing, Smith et al., 2009). MIGLU (600 mg/kg/day, Oxford GlycoSciences/Celltech, UK) and curcumin (Sigma, 150 mg/kg/day) were administered as dry admixtures  as above (from 3 weeks of age). The untreated mice were fed on powdered chow (n=34). Treatment groups were made up of approximately equal numbers of males and females and received ibuprofen (n= 14), curcumin (n = 11), MIGLU (n = 11), curcumin and ibuprofen (n = 5), curcumin and MIGLU (n = 11), or all three therapies (n = 9). | Weight and spontaneous activity of each mouse were recorded weekly until reaching the late humane end-point (loss of 1 g body weight within 24 h) as previously described (Smith et al., 2009). After 5–30 min room acclimatization, the mouse was placed in the ‘open field’ (measuring 45 × 25 × 12 cm). Rearing was recorded manually for 5 min (the number of times the mouse reared on its hind legs with or without support of the cage wall). | We also monitored the mice using rearing ability in an open field to measure motor function/coordination. At 8 weeks of age, rearing was not significantly improved by ibuprofen or curcumin monotherapies but was improved by MIGLU monotherapy and any combination that included MIGLU (untreated Npc1^−/−^, 2.28 events/5 min compared to MIGLU, 22.64; curcumin & MIGLU, 28.82; and triple combination, 41.00 events/5 min, all p<0.001). Rearing activity was significantly improved in both the curcumin and MIGLU dual therapy (p< 0.01) and the triple combination therapy (p< 0.001) compared to MIGLU alone. At 11 weeks of age, only the dual combination of MIGLU and curcumin and the triple combination therapy maintained rearing over the untreated Npc1^−/−^ group (p< 0.01 and p< 0.001, respectively). Strikingly, at this time point the triple combination group demonstrated significant maintenance of rearing activity when compared to the dual therapy (p <0.05). |
| (Caporali et al., 2016) | Npc1nmf164/nmf164 mice with BALB/cJ background (thereafter named Npc1nmf164 mice) obtained from heterozygous crosses. | Because sensorimotor reflexes and motor skills normally appear with a definite timing during the first 3 weeks after birth, they represent a useful tool to assess early postnatal neural development. We therefore evaluated the acquisition of several developmental milestones in the physical and sensorimotor development of Npc1nmf164 mice from PN3 until weaning (PN21). | From postnatal day (PN) 3 to PN21, pups were separated from their dams daily between 9:00 a.m. and 3:00 p.m. for a maximum of 15 min, and tested for physical, postural, locomotor and complex motor behavior development in a warmed environment (30–32 °C). Behavioral assessment evaluated the development of physical parameters (body weight, eye opening, fur appearance, incisor eruption), locomotion (pivoting, crawling, quadrupedal locomotion), swimming performance (direction and limb use), reflex appearance (surface righting reflex, negative geotaxis, cliff avoidance) and complex motor behaviors (ascending a ladder, crossing a narrow bridge, suspension  on a wire). | The Balance beam test (similar to crossing a narrow bridge) measures fine motor coordination and balance. When placed on an elevated round beam, Npc1nmf164 mice crossed significantly fewer beam sections than wt mice did and significantly fewer sections as days went by (main effect of genotype: F1,18 = 34.92, p = 0.00001; main effect of age: F2,36 = 5.08, p = 0.01; interaction between genotype and age: F2,36 = 4.09, p= 0.03). Moreover Npc1nmf164 mice did not differ from wt until PN90 in terms of retention time (main effect of genotype: F1,18 = 54.28, p < 0.00001; main effect of age: F2,36= 6.48, p = 0.004; interaction between genotype and age: F2,36 = 6.01, p = 0.006). |
| (Hung et al., 2016) | BALB/cJ Npc1^nih^ (Npc1^+/–^ ) | In the present study, we examined this possibility in heterozygotes of a natural loss-of-function mutant NPC1 mouse model.  The mice were monitored daily, and from P24, weighed every 2–3 days, then daily on weekdays from P36 until the end of the experiment at P71. | Mice were subjected to motor coordination and balance tests at the age of 5, 7, and 9 weeks. The tests were performed separately on consecutively days at the respective age.  Tests: Rotarod Test, Open-field Test and DigiGait Analysis. | To assess the effect of Npc1 haploinsufficiency on motor coordination and balance, we tested Npc1^+/+^, Npc1^+/–^, and Npc1^–/–^ mice on an accelerating Rotarod at 5, 7, and 9 weeks of age. The data were analyzed as distance travelled on the accelerating Rotarod, which took into account the rotation speed and latency to fall.  There was no significant difference in the distance travelled on the Rotarod by mice of all genotypes between training and testing days (p =0.66). There was a significant interaction of genotype with age (p <0.0001), with the main significant effect between Npc1^+/+^ and Npc1^–/–^ mice, but not Npc1^+/+^ and Np1^+/–^ mice, over age. As expected, the distance travelled by Npc1^–/–^ mice was significantly less than Npc1^+/+^ mice (cube root transformed, b = –2.9; p < 0.0001), and markedly decreased progressively with age. The distance travelled by Npc1^+/–^ mice on the Rotarod did not differ significantly from that of Npc1^+/+^ mice (cube root transformed, b = 0.1; p =0.7). However, examining the change in distance travelled at 7 and 9 weeks of age compared with 5 weeks of age, Npc1^+/+^ mice showed improved motor coordination and balance with maturity (to 9 weeks), while Npc1^+/–^ mice failed to mature in these domains. Male Npc1^+/–^ and Npc1^–/–^ mice were significantly less able to maintain their balance and coordination on the Rotarod compared with female Npc1^+/–^ and Npc1^–/–^ mice, but there was no sex difference in the Rotarod performance of Npc1^+/+^ mice.  To appraise whether Npc1 haploinsufficiency affects spontaneous locomotor activity and novel environment exploratory behavior, we subjected Npc1^+/+^, Npc1^+/–^, and Npc1^–/–^ mice to an open-field test at 5, 7, and 9 weeks of age and analyzed their horizontal and vertical movements for 15 min. Horizontal movement indices in the total arena analyzed were total ambulatory distance travelled, total ambulatory count, and average velocity. Compared with Npc1^+/+^ mice, the total ambulatory distance travelled in the entire arena by the Npc1^+/–^ mice was not different, whereas that of Npc1^–/–^ mice was significantly decreased by 737.6 ± 112.6 cm. There was a significant interaction of genotype with age (p= 0.01), with the main significant effect detected between Npc1^+/+^ and Np1^–/–^ mice over age. By 9 weeks of age, the total ambulatory distance travelled by Npc1^+/+^ mice increased significantly by 183.2 ± 93.5 cm compared with 5 weeks of age (p=0.05), whereas there was no significant change detected in Npc1^+/–^ and Np1^–/–^ mice. The average velocity of Npc1^+/–^ mice movement was significantly slower than Npc1^+/+^ mice, and intermediate between Npc1^+/+^ and Npc1^–/–^ mice.  Vertical movements such as rearing and jumping are normal exploratory behavior of rodents in novel environments.  The incidence rate ratio (IRR) of jump counts showed that the expected jump counts in the total arena of both Npc1^+/–^ and Npc1^–/–^ mice were significantly less than Npc1^+/+^ mice. Npc1^+/–^ and Npc1^–/–^ mice jumped with only about 30%and about 10 %, respectively, of the frequency of Npc1+/+ mice. Overall, male mice jumped with only about 40%of the frequency of female mice. Further comparison of male mice with female mice within each genotype group showed that Npc1^+/+^ and Npc1^+/–^ male mice jumped with only about 10 % and 40 %, respectively, of the frequency of their female counterparts. There was no significant difference in jump count IRR between male and female Npc1^–/–^ mice, which could be attributed to maximal impairment in both sexes. Consistent with decreased jump count IRR, vertical count IRR also revealed significantly reduced vertical rearing activity in Npc1^+/–^ and Npc1^–/–^ mice. The IRRs for vertical rearing counts of Npc1^+/–^ and Npc1^–/–^ mice were only about 40%and 10 %, respectively, that of Npc1^+/+^ mice.  There was a significant interaction of genotype with age for vertical count IRR (p =0.03), with the main significant interaction between Npc1^+/+^ and Npc1^+/–^ mice over age.  Compared with their vertical rearing counts at 5 weeks of age, the Npc1^+/+^ mice had significantly increased IRRs, by about 170% and 190 %, at 7 and 9 weeks of age, respectively, but for the Npc1^+/–^ mice there was no developmental increase in vertical rearing performance at 7 weeks of age, and their performance took until 9 weeks of age to exhibit a significant improvement (about 180 %). As expected, there was no significant improvement in the vertical rearing count over age for Npc1^–/–^ mice, owing to their inherent motor impairment.  In addition to allowing us to examine unforced and voluntary locomotion, the open-field test is classically used to assess anxiety-like behavior in rodents. Mice typically spend a greater amount of time exploring the periphery of an open field arena than the unprotected center area; with the avoidance of the center area interpreted as anxiety. We found that Npc1^–/–^ mice exhibited significantly decreased ambulatory count in the total arena and center ambulatory count IRRs compared with Npc1^+/+^ mice, consistent with motor impairment.  The ambulatory count in the total arena of the Npc1^+/–^ mice did not differ significantly from that of the Npc1^+/+^ mice. However, the ambulatory count IRR of Npc1^+/–^ mice in the center of the arena was only about 60 % that of Npc1^+/+^ mice, indicative of increased anxiety. Male mice entered the center of the arena at about 60 % of the incidence rate of female mice. |
| (Nicoli et al., 2016) | Npc1 mutant (BALB/cNctr-Npc1m1N/J, Npc1^-/-^), control (Npc1^+/+^) and Npc1 heterozygous mice (Npc1^+/-^), were generated from heterozygote breeding. Genotyping was performed as described by Loftus et al. 1997. | Npc1 mutant (BALB/cNctr-Npc1m1N/J, Npc1^-/-^) mice upon weaning at 3 weeks of age began receiving 4000mg/kg HPBCD weekly delivered by IP injection.  Bile Acid Supplementation. Npc1-/- mice, Npc1+/- mice, and Npc1+/+ mice (n = 6 per group) were fed either normal chow (RM1 maintenance diet; SDS, London, UK) or normal chow supplemented with ursodeoxycholic acid (0.5%, w/w, Sigma-Aldrich) mixed with powdered diet. Treatment started at weaning (3 weeks of age) and mice were sacrificed at 6 and 9 weeks of age. | Motor function and coordination were assessed by observational counting of the total rearing events over a period of 5 minutes (either without support or against the cage wall) every week in the open-field. | Rearing (a measure of motor coordination) increased in the Npc1^-/-^ mice treated with UDCA compared to the untreated Npc1^-/-^ mice. While a high degree of variability was seen for all groups, UDCA-treated Npc1-/- mice showed marked improvements, which was significant at 5 weeks of age (p value = 0.0055) and from 7 to 12 weeks (p value = 0.050, p value = 0.002, p value = 0.013, p value = 0.001, p value = 0.0007 and p value = 0.002 respectively). No differences were detected between the Npc1+/+ treated or untreated mice. Npc1^-/-^ mice on UDCA also retained motor function longer than untreated Npc1^-/-^ mice as demonstrated by their ability to rear at 11 weeks, an age at which all untreated Npc1^-/-^ mice are incapable of rearing (p value = 0.0007) |
| (Rabl et al., 2016) | BALB/cNctr-Npc1^m1N^/-J  NPC1−/−mice have a spontaneous mutation in the Niemann-Pick type C1 gene (NPC1m1N). Animals homozygous for the mutation show decreased sphingomyelinase and glucocerebrosi-dase activity and are thus a commonly used model of Niemann-Pick disease (Loftus et al., 1997). Homozygous mice were bred by pairing one heterozygous male with one heterozygous female and off-spring was tested compared to ntg littermates. Only male animals were longitudinally tested at the age of 6 and 8 weeks. | To test rodents for orofacial motor impairments in a stress-free environment, authors established the pasta gnawing test by measuring the biting noise of mice that eat a piece of spaghetti. Two parameters were evaluated, the biting speed and the biting peaks per biting episode. To evaluate the power of this test compared to commonly used limb motor and muscle strength tests, three mouse models of Parkinson’s disease, amyotrophic lateral sclerosis and Niemann-Pick disease were tested in the pasta gnawing test, RotaRod and wire suspension test. | The pasta gnawing test, RotaRod and wire suspension test. | Our results show that the pasta gnawing test reliably displays orofacial motor deficits. Comparison with existing methods: The test is especially useful as additional motor test in early onset disease models, since it shows first deficits later than the RotaRod or wire suspension test. The test depends on a voluntary eating behavior of the animal with only a short-time food deprivation and should thus be stress-free. Conclusions: The pasta gnawing test represents a valuable tool to analyze orofacial motor deficits indifferent early onset disease models.  NPC1^−/−^mice showed first motor impairments in the pasta gnawing test at the age of 8 weeks compared to wildtype animals. Significant differences could be observed in the number of biting peaks per episode but not in the biting speed. Similar results were obtained using the Rota Rod test. The wire suspension time of NPC1^−/−^was not affected. The onset of motor deficits in NPC1^−/−^mice is thus comparable with already published results (Voikar et al., 2002; Zhang et al., 2004). |
| (Schlegel et al., 2016) | 81 male wild-type mice  BALB/c-npc1nihNPC1 | Starting at postnatal day 7 (P7) and thenceforth, mice of the COMBI-group were injected weekly with HPßCD/ALLO (25 mg/kg ALLO dissolved in 40% HPßCD in Ringer’s solution, 4000 mg/kg, i.p., all from Sigma-Aldrich,Munich, Germany). Additionally, these mice were daily injected with MIGLU, dissolved in 0.9% NaCl solution, 300 mg/kg i.p. (N-butyldeoxynojirimycin, Zavesca; Actelion Pharmaceuticals, San Francisco, CA, USA) from P10 to P23. From P23 onwards until termination of experiments mice were fed standard chow with embedded MIGLU resulting in daily intake of 1200 mg/kg MIGLU. The MIGLU-group was treated like the COMBI-group, but without administration of HPßCD/ALLO, instead mice got vehicle. Mice of the sham-group were injected like those of the COMBI-group at the various time points with the respective volumes of 0.9% NaCl or without volume and were fed with chaw without drugs. | The battery of behavioral tests consisted of accelerod, Morris water maze, elevated plus maze, open field and hot-plate tests. | Miglustat-treated wild-type mice displayed impaired spatial learning compared to sham- and combination-treated mice. Both combination- and miglustat-treated mice showed enhanced anxiety in the elevated plus maze compared to sham-treated mice.  For evaluating motor coordination and balance, the accelerod test was performed. Animals of all groups learned the task during both training trials, indicated by decreasing numbers of down falls during the course of the training (P35: F14, 546 = 2.944, p < 0.001; P60: F14, 546 = 2.223, p = 0.006). Sham-treated mice started worse than miglustat- and combination-treated mice. All pairwise multiple comparison procedures (Holm–Sidak method) revealed significant differences (p < 0.001) for training trials 1 and 2 at p35 and for training trial 1 at P60 (Figure 2A,B). However, during further training we did not detect any significant differences in motor performance between the three treatment groups. During probe trials with accelerating speed of the treadmill we also detected no statistically significant difference between the treatment groups with regard to reached speed at down fall at P35 and P60 (P35: F2, 79 = 2.012, p = 0.141; P60: F2, 79 = 2.684, p = 0.074) (Figure 2C,D). In conclusion, neither miglustat nor combination treatment caused alteration of motor coordination and balance in comparison with sham-treated mice.  The Open Field test was conducted to assess explorative locomotor activity and anxiety. Like in all other tests, there was no difference in walking speed (Figure 5A) between all three groups (H2 = 5.605, p = 0.061). By analyzing the ratio of center distance to total distance (Figure 5B) we observed no significant differences between the three treatment groups (F2, 66 = 2.99, p = 0.057). In addition, we observed no significant differences between all three groups by analysis of the ratio of center to total visits (Figure 5C). One way ANOVA revealed the existence of significant differences (F2, 66 = 3.614, p = 0.032), but pairwise multiple comparison procedures (Holm–Sidak method) revealed no significant differences between particular groups. |
| (Kulkarni et al., 2018) | Npc1^nmf164^ mice with BALB/cJ | To determine the efficacy of ORX-301 prodrug Npc1^nmf164^ and age matched wt mice were subjected weekly to a subcutaneous injection of a 16% w/v ORX-301 solution in PBS (800mg/kg body weight). Control group mice received plain PBS (sham, group). Npc1^nmf164^ and wt mice were randomly assigned to the various experimental groups, which had a comparable number of males and females, ruling out possible bias related to the sex. In the late intervention study, Npc1^nmf164^ and wt mouse littermates were treated weekly with either ORX-301 or PBS starting from the 7th week of age. | The motor co-ordination phenotype was assessed by the balance beam test every two weeks as previously described (Caporali, P. et al. (2016). Briefly, the mouse was placed perpendicularly at the center of a horizontal round beam (covered with paper tape, outer diameter 2 cm, length 1 m, divided into 10 sections and placed 50 cm above a padded surface) and the number of beam sections crossed in a 180 s time interval was recorded. Although the test relies on muscle strength and limb tone, it is well validated for the assessment of fine motor coordination and balance. In addition, as was previously demonstrated, the balance beam test is very sensitive to detect subtle motor impairment in *Npc1^nmf164^* mice before the appearance of overt ataxic signs (Caporali, P. et al. (2016). | Motor behavior studies demonstrated that both the ORX-301 and HPβCD treatments appeared to improve the performance of *Npc1^nmf164^* mice in the balance beam test. This improvement in motor behavior is consistently observed in ORX-301-treated mice up to the end of the behavioral assessment, while the improvement associated with HPβCD-treatment is only temporarily observed up to 9-weeks of age. |
| (Santiago-Mujica et al., 2019) | [BALB/cNctr-Npc1<m1N>/J (Jackson Laboratories # 003092)] | Treatment: not done.  Here we characterized *NPC1^–/–^* mice for their hepatic and neuronal phenotype to confirm the stability of the phenotype, provide a characterization of disease progression and pinpoint the age of robust phenotype onset. Animals of 4e10 weeks of age were analysed for general health, motor deficits as well as hepatic and neuronal alterations with a special focus on cerebellar pathology. | To assess motor coordination, the Rota Rod and Beam Walk test were performed. | NPC1-/- mice showed a progressive decrease in the latency to fall off of the rod. When compared to WT littermates, significant differences were observed at 8-10 weeks of age.  The Beam Walk test was performed only with 7 week old animals since older animals coordination between NPC1^_/_^ mice and WT littermates could be observed at each of the five beams used (data not shown). NPC1^_/_^ mice presented a significantly increased number of slips compared to WT littermates. The highest amount of slips was observed on the square beam with 10 mm diameter, but even on the 28 mm round beam animals performed significantly worse than WT littermates. |

**References**

Ahmad, I., Lope-Piedrafita, S., Bi, X., Hicks, C., Yao, Y., Yu, C., et al. (2005). Allopregnanolone treatment, both as a single injection or repetitively, delays demyelination and enhances survival of Niemann-Pick C mice. *J Neurosci Res* 82(6)**,** 811-821. doi: 10.1002/jnr.20685.

Alvarez, A.R., Klein, A., Castro, J., Cancino, G.I., Amigo, J., Mosqueira, M., et al. (2008). Imatinib therapy blocks cerebellar apoptosis and improves neurological symptoms in a mouse model of Niemann-Pick type C disease. *The FASEB journal : official publication of the Federation of American Societies for Experimental Biology* 22**,** 3617-3627. doi: 10.1096/fj.07-102715.

Bascuñan-Castillo, E.C., Erickson, R.P., Howison, C.M., Hunter, R.J., Heidenreich, R.H., Hicks, C., et al. (2004). Tamoxifen and vitamin E treatments delay symptoms in the mouse model of Niemann-Pick C. *Journal of Applied Genetics* 45**,** 461-467.

Borbon, I.A., Hillman, Z., Duran, E., Kiela, P.R., Frautschy, S.A., and Erickson, R.P. (2012). Lack of efficacy of curcumin on neurodegeneration in the mouse model of Niemann-Pick C1. *Pharmacology Biochemistry and Behavior* 101**,** 125-131. doi: 10.1016/j.pbb.2011.12.009.

Caporali, P., Bruno, F., Palladino, G., Dragotto, J., Petrosini, L., Mangia, F., et al. (2016). Developmental delay in motor skill acquisition in Niemann-Pick C1 mice reveals abnormal cerebellar morphogenesis. *Acta neuropathologica communications* 4**,** 94. doi: 10.1186/s40478-016-0370-z.

Chen, G., Li, H.M., Chen, Y.R., Gu, X.S., and Duan, S. (2007). Decreased estradiol release from astrocytes contributes to the neurodegeneration in a mouse model of Niemann-Pick disease type C. *GLIA* 55**,** 1509-1518. doi: 10.1002/glia.20563.

Griffin, L.D., Gong, W., Verot, L., and Mellon, S.H. (2004). Niemann-Pick type C disease involves disrupted neurosteroidogenesis and responds to allopregnanolone. *Nat Med* 10(7)**,** 704-711. doi: 10.1038/nm1073.

Hallows, J.L., Iosif, R.E., Biasell, R.D., and Vincent, I. (2006). p35/p25 is not essential for tau and cytoskeletal pathology or neuronal loss in Niemann-Pick type C disease. *Journal of Neuroscience* 26**,** 2738-2744. doi: 10.1523/JNEUROSCI.4834-05.2006.

Hovakimyan, M., Maass, F., Petersen, J., Holzmann, C., Witt, M., Lukas, J., et al. (2013). Combined therapy with cyclodextrin/allopregnanolone and miglustat improves motor but not cognitive functions in Niemann-Pick Type C1 mice. *Neuroscience* 252**,** 201-211. doi: 10.1016/j.neuroscience.2013.08.001.

Hung, Y.H., Walterfang, M., Churilov, L., Bray, L., Jacobson, L.H., Barnham, K.J., et al. (2016). Neurological Dysfunction in Early Maturity of a Model for Niemann-Pick C1 Carrier Status. *Neurotherapeutics* 13(3)**,** 614-622. doi: 10.1007/s13311-016-0427-5.

Kulkarni, A., Caporali, P., Dolas, A., Johny, S., Goyal, S., Dragotto, J., et al. (2018). Linear Cyclodextrin Polymer Prodrugs as Novel Therapeutics for Niemann-Pick Type C1 Disorder. *Scientific Reports* 8. doi: 10.1038/s41598-018-27926-9.

Li, H., Repa, J.J., Valasek, M.A., Beltroy, E.P., Turley, S.D., German, D.C., and Dietschy, J.M. (2005). Molecular, anatomical, and biochemical events associated with neurodegeneration in mice with Niemann-Pick type C disease. *J Neuropathol Exp Neurol* 64(4)**,** 323-333. doi: 10.1093/jnen/64.4.323.

Nicoli, E.R., Eisa, N.A., Cluzeau, C.V.M., Wassif, C.A., Gray, J., Burkert, K.R., et al. (2016). Defective cytochrome p450-catalysed drug metabolism in Niemann-Pick type C disease. *PLoS ONE* 11. doi: 10.1371/journal.pone.0152007.

Rabl, R., Horvath, A., Breitschaedel, C., Flunkert, S., Roemer, H., and Hutter-Paier, B. (2016). Quantitative evaluation of orofacial motor function in mice: The pasta gnawing test, a voluntary and stress-free behavior test. *Journal of Neuroscience Methods* 274**,** 125-130. doi: 10.1016/j.jneumeth.2016.10.006.

Santiago-Mujica, E., Flunkert, S., Rabl, R., Neddens, J., Loeffler, T., and Hutter-Paier, B. (2019). Hepatic and neuronal phenotype of NPC1 −/− mice. *Heliyon* 5(3)**,** e01293. doi: 10.1016/j.heliyon.2019.e01293.

Schlegel, V., Thieme, M., Holzmann, C., Witt, M., Grittner, U., Rolfs, A., and Wree, A. (2016). Pharmacologic Treatment Assigned for Niemann Pick Type C1 Disease Partly Changes Behavioral Traits in Wild-Type Mice. *Int J Mol Sci* 17(11). doi: 10.3390/ijms17111866.

Võikar, V., Rauvala, H., and Ikonen, E. (2002). Cognitive deficit and development of motor impairment in a mouse model of Niemann-Pick type C disease. *Behav Brain Res* 132(1)**,** 1-10. doi: 10.1016/S0166-4328(01)00380-1.

Williams, I.M., Wallom, K.L., Smith, D.A., Al Eisa, N., Smith, C., and Platt, F.M. (2014). Improved neuroprotection using miglustat, curcumin and ibuprofen as a triple combination therapy in Niemann-Pick disease type C1 mice. *Neurobiol Dis* 67**,** 9-17. doi: 10.1016/j.nbd.2014.03.001.

Zhang, M., Li, J., Chakrabarty, P., Bu, B., and Vincent, I. (2004). Cyclin-dependent kinase inhibitors attenuate protein hyperphosphorylation, cytoskeletal lesion formation, and motor defects in Niemann-Pick Type C mice. *Am J Pathol* 165(3)**,** 843-853. doi: 10.1016/S0002-9440(10)63347-0.
