# Supplementary material for: Differently increased volumes of multiple brain areas in Npc1 mutant mice following various drug treatments
Source: Front Neuroanat. 2024 Jul 16;18:1430790. doi: 10.3389/fnana.2024.1430790 (PMC11286580; doi:10.3389/fnana.2024.1430790)
Supplement: Supplementary file 1 [file Data_Sheet_1.ZIP › Supplementary Table 1.docx]

**Supplementary Table 1**. Overview of results of quantification of brain parameters of *Npc1* mice treated with various drugs.

| **Publication and NPC1 modell** | **Brain region** | **Morphological changes/Pathology** | **Treatment** | **Treatment effect** |
| --- | --- | --- | --- | --- |
| **(Baudry et al., 2003)**  Breeding pairs of BALB/cNctr–npc1N mice heterozygous  for NPC ( + npc/_npc) were obtained from Jackson  Laboratories (Bar Harbour, ME, USA) | Cerebellum  Ventral and Lateral  Thalamus  Dorsal Hippocampus | The present study was directed at evaluating the postnatal development  of pathological changes in microglia and astrocytes,  and in particular, to compare it with that of neuronal  degeneration in the brains of NPC1^_/_^ mice and their  corresponding wild type. | No treatment, In the present study, the postnatal development of pathological manifestations of inflammation in several brain regions of NPC1^_/_^  mice were analysed. | Mice lacking the NPC1 gene exhibit several pathological features of NPC patients and have been widely used to provide insights into the mechanisms of the disease. Brain sections from NPC1^-/-^and wild-type (NPC1^+/+^) mice were immunostained with the MAC1 antibody, which recognizes  microglia, with antibodies against glial fibrillary acidic protein (GFAP), which recognize astrocytes, and with antibodies against the cytokine  interleukin-1h (IL-1h). Numbers of MAC1 ir cells were markedly increased in several brain regions of NPC1^_/_^ mice as early as 2 weeks of age. This effect was particularly evident in globus pallidus, ventral lateral thalamus, medial geniculate nucleus, and  cerebellum. MAC1-immunopositive cells had enlarged cell bodies and shorter processes, suggesting they were in an active state. By 4 weeks,  most brain structures exhibited enhanced microglial activation in NPC1^-/-^ mice, and this was maintained at 12 weeks. At 2 weeks, reactive  astrocytes were only observed in the ventral lateral thalamus while they were present throughout the brain of NPC1^-/-^ mice at 4 weeks of  age. The astroglial reaction coincided with up-regulation of the cytokine, interleukin-1h, in most, but not all brain regions. In particular, no interleukin-1h up-regulation was observed in regions devoid of neuronal degeneration. These results suggest that microglial activation precedes and might be causally related to neuronal degeneration, while astrocyte activation might be a consequence of neuronal degeneration.  Quantitative analysis of microglial activation in 2- and 4-week-old wild-type and NPC1^-/-^ mouse brains:  Area (%), 2 weeks-old:  Cerebellum: NPC1^-/-^ 5%, Wildtyp: 1%  Ventral, Lateral Thalamus NPC1^-/-^: 6%, Wildtyp: 0,5%  Dorsal Hippocampus: NPC1^-/-^: 4%, Wildtyp: 1%  Area (%), 4 weeks-old:  Cerebellum: NPC1^-/-^: 3%, Wildtyp: 1%  Ventral, Lateral Thalamus: NPC1^-/-^: 6%, Wildtyp: 0,5%  Dorsal Hippocampus: NPC1^-/-^: 6%, Wildtyp: 1% |
| **(Beltroy et al., 2005)**  BALB/c-npc1^nih^  NPC1^–/–^  mouse model npc1^nih^ | Whole brain volume | Initial experiments were undertaken to quantify the effect of the *npc1* mutation on the growth of the whole animal, and on the absolute and relative weights of the liver and brain, the two organs primarily affected by this disease. | All animals were fed *ad*  *libitum* a low-cholesterol rodent diet (no. 7001; Harlan Teklad, Madison, WI) after weaning at 19 days of age. This diet had a cholesterol content of 0.016% (wt/wt) and a total lipid content of 5% (wt/wt).  The current studies were  undertaken to delineate in detail the biochemical, histological, and molecular abnormalities that occur in the  liver of the NPC mouse maintained on low levels of dietary lipid intake. | The absolute weight of the  brain was less in mutant mice. However, when corrected for the lower body weights seen in these  *npc1*^_/_^ animals, relative brain weight was the same in the mutant and control mice.  Brain weight (from 0 days und 75 days age)  NPC^+/+^: 0.1g – 0.4g  NPC-/-: 0.1g – 0.3g  As a percentage of whole body weight  (from 0 days und 75 days age)  NPC^+/+^: 5.5% - 2%  NPC^-/-^: 5.5% – 2.5% |
| **(Byun et al., 2013)**  BALB/c mice adult wild-type control (NPC^+/+^), hetereozygous  (NPC^+/_^) and homozygous (NPC^_/_^) animals of both sexes were used.  BALB/c *npc^nih^* | Hippocampus and cerebellum | The difference in expression levels of the calcium binding proteins, c-fox, and COX-1,2 in the hippocampus, the most susceptible region in the brain, of young and adult NPC+/+, NPC^+/_^, and NPC^_/_^ mice using immunohisto-chemistry was investigated. | Thus, they used immunohistochemistry to assess the expression levels of calcium binding proteins (calbindin D28K, parvalbumin, and calretinin), c-Fos and  cyclooxygenase-1,2 (COX-1,2) in the hippocampal formation and cerebellum of 4 and 8 week old NPC+/+, NPC^+/_^, and NPC^_/_^ mice. | The number of neurons were similar in all three types.  The stratum pyramidale of the CA1 and CA3  areas was stained dominantly. The stratum lucidum and granular layer of the DG were stained more than that of the other layers, and no differences were observed in the neuronal populations among the NPC+/+, NPC+/_, and NPC^_/_^ mice . Cerebellum also showed similar results among NPC^+/+^, NPC^+/_^ and NPC^_/_^ mice.  Number of neuronal cells in hippocampus of NPC^+/+^; NPC^+/_^; NPC^_/_^ (appr. 3000/mm^2^)  Distribution of neurons in the hippocampal regions (CA1, CA3, and DG) of the NPC^+/+^; NPC^+/_^; NPC^_/_^ mice  CA1: appr. 2800; 2900; 2700/mm^2^ (cell number/mm^2^)  CA3: 2000; 1800; 2000 cell number/mm^2^  DG: approx. 3000 cell number/mm^2^ |
| **(Byun et al., 2011)**  BALB/c mice adult wild-type control (NPC^+/+^), hetereozygous  (NPC^+/_^) and homozygous (NPC^_/_^) animals of both sexes were used.  BALB/c *npc^nih^* | CA2&3 area of the  hippocampus | 12 week-old NPC^_/_^ mouse showed significant decrease in cell number in the CA2&3 area of the hippocampus compared to NPC^+/+^ or ^+/_^ mice of the same age  after cresyl violet staining. | To understand neuronal pathways connecting to the hippocampus, retrograde transneuronal labeling method with Bartha strain of pseudorabies virus (PRV) was employed in 40 NPC^+/+^, NPC^+/_^ and NPC^_/_^ mice. | The number of PRV positive cell was significantly decreased in  several regions including the entorhinal and piriform cortex in the NPC^_/_^ mouse. More severely, lateral  septal dorsal nucleus, dorsal entorhinal cortex and medial geniculate body showed no positive labeling  in the NPC^_/_^ mouse. However, the hippocampus, medial septal and supramammilary nuclei showed  increased immunoreactivity in the NPC^_/_^ mouse. |
| **(Cabeza et al., 2012)**  BALB/c mice carrying a heterozygous mutation in the  Npc1 gene | **Medial septum (MS)**  Septal cholinergic neurons were defined  using anatomical landmarks in accordance with the mouse brain atlas. The anterior commissure and anterior and lateral ventricles defined the ventral border of the. The meeting of the body of the corpus callosum at the midline marked the anterior boundary of the MS, and the midline crossing of the anterior commissure and the appearance of the fornix  marked the posterior boundary. | Neurotrophin receptors are active in signaling endosomes, which are organelles that propagate neurotrophin signaling along neuronal processes. The aim of this work was to assess whether the endosomal and lysosomal alterations observed in NPC disease disrupt neurotrophin signaling. As models, we used NPC1-deficient mice to evaluate the central cholinergic septo-hippocampal pathway and its response to nerve growth factor (NGF) after axotomy. | No treatment  Cholinergic neurons from the MS of WT and NPC1-/- mice were visualized by immunohistochemistry  against choline-acetyl transferase (ChAT) and p75. | Although the number of cholinergic neurons in the MS remained unchanged, the morphology of MS cholinergic neurons was clearly distorted. Neurons appeared to be rounder and hypertrophic, with reduced densities of proximal and distal fibers. Cholinergic fibers in the MS were labeled with an antibody against p75 because this antibody better defines the cholinergic fibers. In addition to cholinergic fiber  abnormalities, we also observed a five-fold increase in axonal spheroid staining for neurofilaments in the MS.  Axonal spheroids are focal axonal swellings filled with organelles and cytoskeletal proteins and are one of the first neuropathological hallmarks of axonal disease. These morphological  abnormalities correlate with the fact that NPC1-/- cholinergic cells of the MS were loaded with cholesterol, confirming that MS cholinergic  neurons are affected by NPC1 deficiency.  Characterization of NPC1-/- septal cholinergic neurons: Brain sections at the level of the medial septum (MS) from 8- week-old WT and NPC1-/- mice were stained for ChAT and visualized with secondary antibodies conjugated to HRP. WT cholinergic neurons are smaller and less rounded than NPC1-/- cholinergic neurons, and there is a reduction in the number of  cholinergic fibers surrounding the labeled NPC1-/- cholinergic cells.  The number of ChAT-labeled cholinergic neurons was quantified in the septal area of WT and NPC1-/- brain sections. There are no differences in the number of septal  cholinergic neurons. The morphology of septal cholinergic neurons was evaluated in ChAT-stained brain sections from WT and NPC1-/- mice. NPC1-/- cells have an increased area and exhibit a higher ratio between the minor and major axes of cholinergic neurons.  Cholinergic neuron in the MS number:  WT: about 190 (4 sections)  NPC1^-/-^: about 190 |
| **(Chandler et al., 2017)**  Heterozygous Npc1þ/- mice (BALB/cNctr-Npc1m1N/J strain) were bred to obtain control (Npc1þ/þ) and mutant  (Npc1^-/-^) littermates. | Cerebellum | Loss of cerebellar Purkinje neurons | To test the potential efficacy of gene therapy for NPC1, we constructed adeno-associated  virus serotype 9 (AAV9) vectors to deliver the NPC1 gene under the transcriptional control of the neuronal-specific (CamKII) or a ubiquitous (EF1a) promoter. The Npc1^-/-^ mice that received a single dose of AAV9-CamKII-NPC1 as neonates  (2.6x10^11^GC) or at weaning (1.3x10^12^GC), and the mice that received a single dose of AAV9-EF1a-NPC1 at weaning  (1.2x10^12^GC). | Following immunohistochemical evaluation of Purkinje cell density at 9 weeks of age in the experimental group, a significant delay was noted in the typical anterior-to-posterior loss of these neurons upon AAV9-CamKII-NPC1 treatment. In the Npc1þ/þ control mice, Purkinje cell numbers remained at normal levels (31.01, 26.94 and 29.51 cells/mm of Purkinje cell loss in lobules VI, VII  and IX respectively), but the large-scale neuron loss was observed  in Npc1^-/-^ control mice ( 2.43, 3.52 and 9.46 cells/mm of pcl in lobules VI, VII and IX respectively). While Purkinje cell  loss had initiated in the anterior lobules I-V in AAV9-CamKIINPC1 treated Npc1^-/-^ mice), significantly more neurons remained when compared to the Npc1-/- control mice), with 9.37 cells/mm of the Purkinje cell layer remaining in lobule VI, 9.71 cells/mm of Purkinje cells remaining in lobule VII), and 17.22 cells/mm of Purkinje cells remaining in lobule IX. These data are  suggestive of a secondary AAV9-CamKII-NPC1 gene therapy mediated delay of Purkinje cell death and motor function.  decline. |
| **(German et al., 2001)**  BALB/c-npc1^nih^  NPC1^–/–^  mouse model npc1^nih^ | Prefrontal cortex  Thalamus  Cerebellum  Corpus callosum  Basal Forebrain  Striatum | The BALB/c mouse model of Niemann-Pick type C (NPC) disease exhibits neuropathological  similarities to the human condition. There is an age-related cerebral atrophy, demyelination of the  corpus callosum, and degeneration of cerebellar Purkinje cells in the NPC mouse. | The purpose of the present study was to determine whether neurodegeneration occurs in the NPC mouse, in brain regions other than the cerebellum and whether the degeneration is related to the presence of neurofibrillary tangles. Using light microscopic methods with immunohistochemistry, electron microscopy, and cell counting methods, 11-week-old NPC^+/+^ and NPC^−/−^ animals were examined.  To quantify the number of neurons and glial cell bodies in the NPC mouse, we estimated the total number of cells in four brain regions by using stereological counting  methods. Neurons were counted in the prefrontal cortex and thalamus, as representations of cortical and subcortical brain regions, respectively. Purkinje cells were counted in the cerebellum because this region has been found to degenerate in an age-dependent manner in the NPC mouse (Tanaka et al., 1988). Glial cells were counted in the corpus callosum, because this structure contains only the cell bodies of glial cells. | In the 11-week-old NPC^−/−^ mouse, which represents the maximum life span of this mouse type, the greatest magnitude of neuronal loss (96%) occurs within the cerebellum in Purkinje cells. In these same mice, however, there is only 20–28% loss of neurons in the prefrontal cortex and entire thalamus. Furthermore, there is a 63% loss of glial cells in the corpus callosum. However, striatal and basal forebrain cholinergic neuron numbers appear normal.  NPC^+/+^ (cell number):  Prefrontal cortex 546,750 ± 20,314  Thalamus 267,907 ± 5,921  Cerebellum (Purkinje cells were counted) 71,959 ± 2,176  Corpus callosum (glial cell somata were counted) 225,600 ± 22,650  NPC^-/-^ (cell number):  Prefrontal cortex 391,875 ± 18,497  Thalamus 214,447 ± 11,108  Cerebellum (Purkinje cells were counted) 2,697 ± 312  Corpus callosum (glial cell somata were counted) 83,178 ± 3,183  NPC^+/+^ Volume (mm^3^)  Prefrontal cortex 5.65 ± 0.18  Thalamus 3.02 ± 0.03  Cerebellum 11.44 ± 0.22  Corpus callosum 0.60 ± 0.01  NPC^-/-^ Volume (mm^3^)  Prefrontal cortex 4.56 ± 0.20  Thalamus 2.69 ± 0.06  Cerebellum 8.15 ± 0.31  Corpus callosum 0.33 ± 0.01  Cholinergic Cell  NPC^+/+^  Basal Forebrain 70.7 ± 8.2 (cell number); Striatum 77.5 ± 9.2 (cell number). 15.11 ± 0.36 (cell size)  NPC^-/-^  Basal Forebrain 77.7 ± 2.2 (cell number); Striatum 69.0 ± 13 (cell number), 16.51 ± 0.24 (cell size) |
| **(Griffin et al., 2004)**  A strain of BALB/c mice, designated NCTR-BALB/c, carrying a new genetic disorder characterized by excessive tissue deposition of cholesterol and phospholipid. | Cerebellum (Purkinje cells) | Cerebellar degeneration, Purkinje cells degeneration | Mice received one of three treatments: 0.5–2 μM allopregnanolone in drinking water, with the concentration increasing by 0.5 μM every 2 weeks; a subcutaneous time-release pellet (250 mg over 90 d); or a single subcutaneous injection of 25 mg/kg of allopregnanolone in 20%  β-cyclodextrin (2-hydroxypropyl-β-cyclodextrin; Sigma), corresponding to  1.25 mg of allopregnanolone per ml of 20% β-cyclodextrin. | Untreated NP-C mice had fewer  Purkinje and granule cell neurons than wild-type mice, but allopregnanolone treatment improved Purkinje and granule cell survival. Purkinje loss in NP-C mice was greatest in lobes 4–8,  and allopregnanolone treatment had the greatest effect on Purkinje  survival in lobes 4–10. Granule cell neurons were also lost in untreated NP-C mice, but these neurons were spared in allopregnanolone-treated mice.  Purkinje cells (% of wild-type)  Lobe 1:  WT – 100%  NPC – untreated - 80%  P7 (NPC-treated with allopregnalone at P7) – 90%;  Lobe 2:  WT – 100%  NPC – untreated - 60%  P7 (NPC-treated with allopregnalone at P7) – 70%;;  Lobe 8:  WT – 100%  NPC – untreated - 40%  P7 (NPC-treated with allopregnalone at P7) – 90%;  Lobe 9&10:  WT – 100%  NPC – untreated - 60%  P7 (NPC-treated with allopregnalone at P7) – 100%; |
| **(Ko et al., 2005)**  BALB/c npc1nih, FVB.Cg-Tg(GFPU)5Nagy/J, and C57BL/6J  mice were obtained from Jackson Laboratory (Bar Harbor, Maine,  United States). | Cerebellum  (Purkinje cells; PC loss in the cerebellar vermis progressed in an anterior (lobule I) to posterior (lobule X) sweep) | Cells lacking Npc1, which is a transmembrane protein related to the Hedgehog receptor Patched, or Npc2, which is a secreted cholesterol-binding protein, have aberrant organelle trafficking and accumulate large quantities of cholesterol and other lipids. Though the Npc proteins are produced by all cells, cerebellar Purkinje  neurons are especially sensitive to loss of Npc function. | No treatment. | PC loss in the cerebellar vermis progressed in an anterior (lobule I) to posterior (lobule X) sweep. There was a normal number and density of PCs in cerebellar sections from 30-d-old npc1^_/_^ mice. At 50 d, most PCs in lobules I–III were no longer present. By 70 d, only lobule X PCs remained intact.  Quantification of progressive anterior-to-posterior PC loss. PC densities from 30-d npc1þ/þ and 30-, 50-, and 70-d npc1^_/_^ mice were quantified from  five sections from two mice each. |
| **(Li et al., 2005)**  BALB/c *npc^nih^*  Heterozygous NPC1 mice with a BALB/c background  were mated.  These crosses generated control  (NPC1+/+) and homozygous null (NPC12/2) animals of both  sexes. | Purkinje cells were counted in a region outlined to include only the Purkinje cell layer. Glial cells were counted  within the rostral half of the corpus callosum, since the only somata in this structure were those of glial cells.  Stereologic estimation of cell numbers and regional brain volumes in four different areas of the CNS in NPC1^-/-^ mice at 3, 7, and 11 weeks of age. Absolute numbers of neurons were quantified in the prefrontal cortex and thalamus while only the Purkinje cells were counted in the cerebellum. Only glial cells were quantified in the corpus callosum (B). The volumes of these same 4 regions were also estimated.  Relative brain weight | Purkinje cell loss in the cerebellum begins at 3 to 4 weeks of age and is nearly complete by 11 weeks.  Nevertheless, neurodegeneration proceeds  utilizing apoptosis with activation of glial cells, increased apoE and apoD synthesis, and increased cholesterol turnover across the CNS. | To first evaluate the interaction of gender and cholesterol intake, 4 groups of NPC^-/-^ and NPC1^+/+^ mice were weaned onto diets containing either 0.02% or 1.0% cholesterol and  observed for the duration of their lives.  Another important issue was whether cholesterol  feeding might affect some aspect of the neurodegeneration  taking place in the CNS. | Neural and glial cell numbers were quantified in several regions of the brain. Notably, in both the prefrontal cortex and thalamus, there was relatively little loss of nerve cells. In contrast, in the cerebellum, 70% of the Purkinje cells had disappeared by 7 weeks of age and these cells were nearly gone by 11 weeks.  The volumes of these 3 regions of the brain, were reduced relatively little and these small reductions were due, in part, to the fact that both brain and whole body weights were lower in the NPC1^-/-^animals.  In NPC1^-/-^ mice Prefrontal cortex (absolute numbers of neurons):  Relative cell number (3, 7 and 11 weeks of age) (% Control, NPC1^+/+^ - 100%): 95%, 70%, 70%; volume of the region: 95%, 80%, 80%.  Thalamus (absolute numbers of neurons): 95%, 90%, 70%; volume of the region: 80%, 70%, 90%.  Corpus callosum (glial cells): 50%, 40%, 40%; volume of the region: 75%, 60%, 60%.  Cerebellum (Purkinje cells): 90%, 20%, 0%; volume of the region: 90%, 80%, 70%.  Relative brain weight (3, 7 and 11 weeks of age), expressed as a percentage of whole body weight:  NPC1^+/+^ mice: 6%, 3%, 2%.  NPC1^-/-^ mice: 6%, 3%, 2%. |
| **(Liu et al., 2009)**  BALB/c-npc1^nih^  Effect of combination therapies on microglial recruitment in the NPC1−/− cerebellum.  mouse model npc1^nih^ | Effect of CYCLO on pyramidal and Purkinje cell number in the cerebellum. | In the cerebellum, there was marked loss of Purkinje cells, particularly in the anterior-superior cerebellar vermis, and this loss was accompanied by a proportional degree of Bergmann gliosis.  Storage material identical to that seen in the other perikarya was present in the cytoplasm of the remaining Purkinje cells. | Mice were administered a single s.c. injection during the late dark phase at the scruff  of the neck at 7 days of age of a 20% (wt/vol in saline) solution of CYCLO (4,000  mg/kg bw) with either 5.6° of substitution (product 332607; Aldrich) or 4.5° of substitution (product H107; Sigma). In some experiments, Allo (P 8887; Sigma) was added to the CYCLO solutions at a concentration of 1.5 mg/ml (25 mg/kg bw) (15, 16). Matching mice injected with saline only served as controls. | Although the single dose of CYCLO administered at 7 days of age  did not alter the number of pyramidal cells present in the cerebral  cortex of these 49-day-old animals (relative pyramidal cell number in % was 100% in NPC1^−/−^ and NPC1^+/+^ group), the number of surviving Purkinje cells in the cerebellum was more than doubled: relative Purkinje cell number in % was 40% in NPC1^−/−^ and 100% in NPC1^+/+^. This beneficial effect was also reflected in the substantial increase in calbindin immunoreactivity and the reduction in GFAP immunoreactivity seen in the cerebellum, compared with untreated animals. |
| **(Lopez et al., 2012)**  Mice homozygous for the Ccl3-targeted mutation  (JAX Mice Strain Name: B6.129P2-Ccl3tm1Unc/J) were obtained  from The Jackson Laboratory. These C57BL/6 Ccl32/2 mice were crossed to congenic FVB Npc12/2 mice (originally JAX  Mice Strain Name: BALB/cNctr-Npc1m1N/J). | Cerebellum; lobule III (Purkinje cells)  Cerebellar lobule III  was chosen for analysis because of its consistent morphology across mice of various ages and its early loss of Purkinje neurons compared with more posterior lobules. | Chronic systemic inflammation is thought to be a major contributor to metabolic and neurodegenerative diseases.  Since inflammatory components are shared among different disorders, targeting inflammation is an  attractive option for mitigating disease. To test the significance of inflammation in the lipid storage disorder  (LSD) Niemann-Pick C (NPC), we deleted the macrophage inflammatory gene Mip1a/Ccl3 from NPC diseased mice. | Authors tested whether suppression of the production of Purkinje neuron NPC1-YFP (a functional NPC1-YFP protein in cerebellar Purkinje neurons) in P; N; Npc12/2 mice with Dox  can elicit neurodegeneration. | In male FVB Npc12/2 mice, Purkinje neuron loss starts to become apparent  in lobule III by age P50. By P65, Npc12/2 mice tend to be devoid of Purkinje neurons in this cerebellar region. In sharp contrast, P; N; Npc12/2 mice at age P88 have considerably more neurons surviving in the lobule III region of the cerebellum  than Npc12/2 mice older. When Dox is administered continuously to P; N; Npc12/2 mice starting at P77, neuron loss commences. At P88, P; N; Npc12/2  mice that had been treated with Dox starting at P77 showed Purkinje  neuron loss that resembles the degree of Purkinje neuron loss that occurs in Npc12/2 mice between P52 and P65. Thus, removing NPC1 function from  neurons of adult P; N; Npc12/2 mice triggers degeneration of neurons.  Representative quantitative analysis of  the number of remaining neurons in lobule III at a given age and per trial. Two mice were used per trial and trials I, II, III and IV were performed in duplicate. The number of Purkinje neurons for wild-type (WT) mice (was calculated by averaging together Npc1 ^+/-^ mice of approximate ages: P50, P60 and P70. For Npc^+/-^ mice of the following ages: P52, P65, P72 and P82), three or more mice were sampled for each age.  Purkinje cells/mm:  WT: 40  Npc^+/-^ mice P72: 5  Npc^+/-^ mice P82: 0  Npc^+/-^ mice P52: 21  Npc^+/-^ mice P65: 8  I: 30  II: 10  III: 20  IV: 8 |
| **(Luan et al., 2008)**  BALB/c-npc1^nih^  NPC1^–/–^  mouse model npc1^nih^ | Sections were taken from (1) upper midbrain level to examine the substantia nigra (SN), the superior colliculus, the oculomotor nucleus, the medial geniculate nucleus, and the rostral interstitial nucleus of the  medial longitudinal fasciculus, (2) lower midbrain level for  examination of the inferior colliculus, (3) midbrain-pons  junctional level to examine the pedunculopontine tegmental  nucleus (PPTG), (4) upper pontine level to examine the locus ceruleus (LC), and (5) four different levels of the upper medulla oblongata to examine the cochlear nucleus (CN), the hypoglossal nucleus (12N), the nucleus ambiguus (NA), and the nucleus of the solitary tract (NTS). | Although some characteristic symptoms of NPC result from brainstem dysfunction, little information is available about  which brainstem structures are affected. In this study, the brainstems of mutant BALB/c NPC1^−/−^ mice with a retroposon insertion in the  NPC1 gene were examined for neuropathological changes. | No treatment. In the present study, we aimed to elucidate subregional differences in the neuropathological changes that occur within brainstem structures in the NPC1^−/−^ mouse. Immunohistochemistry  revealed the specific involvement of certain brainstem structures in this mouse, which can be  related to the pathogenesis of characteristic symptoms of  NPC. | Weight and size of NPC1^−/−^ mouse brains:  Weight (g)  NPC1^−/−^: 0.39±0.02  NPC1^+/+^: 0.49±0.01  Length (mm)  NPC1^−/−^: 12.6±0.3  NPC1^+/+^: 14.7±0.2  Width (mm)  NPC1^−/−^: 10.2±0.1  NPC1^+/+^: 10.9±0.2  Height (mm)  NPC1^−/−^: 6.0±0.1  NPC1^+/+^: 6.8±0.1  Length of cerebrum (mm)  NPC1^−/−^: 8.3±0.1  NPC1^+/+^: 9.2±0.1  Length of medulla oblongatab (μm)  NPC1^−/−^: 1252.5±48.6  NPC1^+/+^: 990.0±44.9  In this study, the brainstems of mutant BALB/c NPC1^−/−^ mice with a retroposon insertion in the NPC1 gene were examined for neuropathological changes. In the midbrain, the integrated optic density (IOD) and cell count density of  tyrosine-hydroxylase (TH) immunostained neurons were decreased in the substantia nigra. In the pons, TH immunoreactivity in the locus ceruleus (LC) neurons was decreased, while the IOD and the neuronal density of choline acetyltransferase (ChAT)-immunostained neurons in  the pedunculopontine tegmental nucleus were preserved. The ChAT immunoreactivity of the hypoglossal nucleus (12N) neurons was not  decreased, but Klüver–Barrera staining showed that neuronal density in the nucleus of the solitary tract (NTS) was decreased. Klüver–Barrera and neuronal nuclei (NeuN) staining showed a decrease in neuronal density in the ventral cochlear nucleus, but not in the dorsal cochlear nucleus. Gliosis was widely identified by GFAP staining in various brainstem structures, including the superior and inferior colliculi, the rostral interstitial nucleus of the medial longitudinal fasciculus, the oculomotor complex, the medial geniculate nucleus, the nucleus  ambiguus, and the 12N. However, GFAP expression was not augmented in the LC, the cochlear nucleus, or the NTS. These neuropathological findings suggest a basis for the neurological syndromes observed in NPC, such as rigidity, oculomotor symptoms,  cataplexy and sleep disturbance, dysphagia, and perceptive deafness.  Densities of neurons were counted in the nucleus of the solitary tract (NTS) and the ventral cochlear nucleus (VCN) using Klüver–Barrera staining, and in the dorsal cochlear nucleus (DCN) using NeuN immunostaining:    Cell number (/2.5x10^-2^mm^2^):  NTSr:  NPC1^−/−^: 220  NPC1^+/+^: 240  NTSc:  NPC1^−/−^: 210  NPC1^+/+^: 220  DCN:  NPC1^−/−^: 390  NPC1^+/+^: 430  VCN:  NPC1^−/−^: 140  NPC1^+/+^: 150  Astrogliosis observed in GFAP-stained sections of each brainstem structure in NPC1^−/−^ mice (n=10, for each group). The ratio of the mean of the IOD (integrated optical density) in  NPC1−/− mice to that in control animals is shown. CN, cochlear nucleus; IC, inferior colliculus; MGN, medial geniculate nucleus; NA, nucleus ambiguus; NTSr, rostral NTS; NTSc, caudal NTS; ON, oculomotor nucleus; riMLF, rostral interstitial nucleus of the medial longitudinal fasciculus; SNc, SN pars compacta; SNr, SN pars reticulata; SC, superior colliculus.  SNc: 5  SNr: 1  riMLF: 4  SC: 7  ON: 5  LC: 1  PPTG: 8  12N: 5  NA: 4  NTSr: 1  NTSc: 1  CN: 1  IC: 60  MGN: 40  Density of neurons and integrated optical density (IOD) on immunostaining in each structure of the brainstem. Neuronal density and IOD were measured in each structure, immunostained with TH (SN and LC) or ChAT (PPTG and hypoglossal nucleus (12N)):  SN TH  IOD  NPC1^−/−^: 13000  NPC1^+/+^: 18000  SN TH  Cell number (/2.5x10^-2^mm^2^):  NPC1^−/−^: 40  NPC1^+/+^: 49  LC TH  IOD  NPC1^−/−^: 5000  NPC1^+/+^: 10000  LC TH  Cell number (/2.5x10^-2^mm^2^):  NPC1^−/−^: 50  NPC1^+/+^: 60  PPTG CHAT  IOD  NPC1^−/−^: 1000  NPC1^+/+^: 2000  PPTG CHAT  Cell number (/2.5x10^-2^mm^2^):  NPC1^−/−^: 9  NPC1^+/+^: 9  12N CHAT  IOD  NPC1^−/−^: 2500  NPC1^+/+^: 3000  12N CHAT  Cell number (/2.5x10^-2^mm^2^):  NPC1^−/−^: 23  NPC1^+/+^: 25 |
| **(Maass et al., 2015)**  BALB/c-npc1^nih^  NPC1^–/–^  mouse model npc1^nih^ | Whole-Brain weight  Volume of the Cerebellar Cortex  Volume of the cerebellar granule cell layer  Lobe VIII of  both hemispheres  Deep  cerebellar nuclei  Number of interneurons in the molecular layer | In NPC1^+/+sham^ brain weight was 0.4746±0.006 g;  In both of the other groups significantly decreased:  NPC1^–/–sham^  0.3896±0.005 g; NPC1^–/–SRT/BPT^ 0.3876±0.004 g compared with NPC1^+/+sham^  There was a reduction in volume of the molecular  layer within the NPC1^–/–sham^ group compared with the  NPC1^+/+sham^ group (32%);  The volume of the cerebellar granule cell layer in  NPC1^–/–sham^ mice was reduced by 17% compared with  the control NPC1^+/+sham^ group;  The volume of the  cerebellar white matter was reduced in the NPC1^–/–sham^  group (20%);  Severe reduction  of Purkinje neurons in NPC1^–/–sham^ mice compared with NPC1^+/+sham^ mice (83%);  A reduction in volume of the Med within NPC1^–/–sham^ compared with NPC1^+/+sham^ (29%)  The number of neurons of Med was significantly reduced (14%);  The volume of Int was decreased in comparison with NPC1^+/+sham^ (29%);  The number of neurons of the Int revealed no reduction in the NPC1^–/–sham^ group;  The number of neurons  was reduced in the Lat in the NPC1^–/–sham^ group (13%);  The Lat volume in NPC1^–/–sham^ mice was reduced compared with NPC1^+/+sham^ mice (38%);    Interneurons were dramatically reduced in NPC1^–/–sham^ animals compared with NPC1^+/+sham^ animals (44%)  Golgi cell number was  significantly reduced NPC1^–/–sham^ animals (16%); | At P7 and thenceforth, NPC1^–/–^ mice were injected weekly with allopregnanolone (25 mg/kg; Sigma Aldrich, St.  Louis, MO) dissolved in b-cyclodextrin (4,000 mg/kg, i.p.;  Sigma Aldrich). At P10 and until P23, animals were injected daily with miglustat (300 mg/kg, i.p.; Zavesca; Actelion Pharmaceuticals,  San Francisco, CA). From P23 onward, animals were fed with miglustat as powdered chow (1,200 mg/kg per day) until termination. Animals that received allopregnanolone/  cyclodextrin/miglustat are referred to as NPC1^–/–SRT/BPT^ mice. The NPC1^–/–sham^ and the NPC11/1sham group were sham-injected with 0.9% NaCl, i.p., at the various time points and were fed powdered chow without drugs. | Volume of the molecular layer with significant difference between  NPC1^–/–sham^ and NPC1^–/–SRT/BPT^ (22%);  NPC1^–/–SRT/BPT^ animals reached a normal wildtype level of volume comparable to that of the NPC1^+/+ sham^  A positive therapy effect with a significant difference between  NPC1^–/–sham^ and NPC1^–/–SRT/BPT^ (22%);  NPC1^–/–SRT/BPT^ animals reached a normal wildtype level of volume, comparable to that of the NPC1^+/+ sham^  The improvement of the cerebellar granule cell layer volume with therapy was not significant  There was no significant therapy  effect on the volume of the cerebellar white matter  The number of the Purkinje neurons increased significantly (a pronounced  positive effect; fourfold)) on Purkinje neurons in the NPC1^–/–SRT/BPT^ group compared with the NPC1^–/–sham^ group;  However, NPC1^–/–SRT/BPT^ animals did not fully reach the normal NPC1^+/+sham^ baseline value of the Purkinje neurons, exhibiting a 29% reduction  No significant therapy effect on the volume of Med and the remaining significant difference in NPC1^–/–SRT/BPT^ and NPC1^+/+sham^ groups was 25%;  The therapy effect on the neurons number was not significant;  There was a positive therapy effect (17%) and Int volume of NPC1^–/–SRT/BPT^ animals reached a normal level;  Although the number of neurons  of the Int revealed no reduction in the NPC1^–/–sham^ group the therapy yielded a cell number increase;  The number of neurons in the Lat was not influenced by combined therapy;  There was a positive therapy effect (18%); and the remaining significant difference within the NPC1^–/–SRT/BPT^ and NPC1^+/+sham^ groups was 24%;  There was a significant  positive therapy effect (30%), and the cell  number in the molecular layer reached normal NPC1^+/+sham^ baseline values;  No significant therapy effect could be  observed |
| **(Ohara et al., 2004)**  A colony of Balb/c npc^nih^ mutant mice has been maintained. | The central processes of dorsal root ganglion (DRG) neurons at the level of the medullary dorsal column nuclei  and the spinal dorsal horn with special attention to the ultrastructural changes of presynaptic axon terminals.  Total number of myelinated axons and total fascicular area of the dorsal roots and ventral roots in NPC and Control were estimated. | A Balb/c-npc1 mutant strain is a genetically authentic murine model of NP-C, and homozygous mice show progressive weight loss and tremor or ataxia until death at 12–14 weeks of age. Neuropathologically, this model is known to faithfully  reproduce the cardinal histologic features of NP-C including neuronal storage, appearance of swollen axons (spheroids), and neuronal loss,  although the cellular mechanisms of neural degeneration are largely unknown. | No treatment, the appearance of axonal spheroids in the dorsal column nuclei and the loss of axons in the spinal nerve roots were assessed quantitatively. | In this study, we employed electron microscopy and morphometric techniques to define structural alterations of the central processes of DRG neurons of Balb/c npcnih, an  authentic murine model of human NP-C with identical genetic and chemical defects.  The gracile nuclei develop numerous axonal spheroids after only 3 weeks. At 6 and 9 weeks, dystrophic axons, which were separated from simple axonal spheroids by the ultrastructural presence of distinctive tubulo-vesicular elements, progressively increased in size and number. These neuropathological findings are identical to those of gracile axonal dystrophy (GAD) of the normal aging mouse. Presynaptic elements were exclusively involved in spheroid formation. The cuneate nuclei and the spinal dorsal horn revealed fewer axonal spheroids and only rare dystrophic changes.  The density of axonal spheroids in NPC mice:  3 weeks; Diameter (μm):  Gracile: ≥5; 5.0 ± 1.4 (8.8); ≥10; 0; ≥15; 0  Cuneate: 0; 0; 0  6 weeks; Diameter (μm):  Gracile: ≥5; 60.2 ± 19.9; ≥10; 13.5±8.1; ≥15; 1.5±1.3;  Cuneate: ≥5; 12.5 ± 3.1; ≥10; 1.5±1.3; ≥15; 0;  9 weeks; Diameter (μm):  Gracile: ≥5; 107.2 ± 13.8; ≥10; 38.0±7.5; ≥15; 10.4±2.9;  Cuneate: ≥5; 30.8 ± 8.4; ≥10; 4.2±1.3; ≥15; 0.4±0.5;  This was associated with a significant drop in the number of L4–5 dorsal root axons in NP-C mouse at 9 weeks of age compared with controls. |
| **(Praggastis et al., 2015)**  A Murine Niemann-Pick C1 I1061T Knock-In Model.  The murine *Npc1* genomic locus was inserted  into a mouse 129 bacterial artificial chromosome  (BAC) construct (BAC identification  number bMQ-398C12). The I1061T mutation,  ATA to ACA at amino acid 1061 in exon 21, was introduced via galactokinase (galK)-based  Recombineering. | Cerebellum (Purkinje cells number) in Anterior Zone (Lobules I-V); Central Zone: (Lobules VI-VII; Posterior Zone: (Lobule VIII); Nodular Zone: (Lobules IX-X); Lobule X only | The progressive loss of motor coordination in NPC1 disease is a well established consequence of the stereotypical Purkinje  cell loss in the cerebellum.  To determine whether the neurological phenotype we observed in the  *Npc1I1061T* mice was associated with Purkinje cell degeneration, we performed calbindin  D immunohistochemistry to visualize these neurons in  parasagittal cerebellar sections from P28, P63, and P105 mice. | No treatment, generation a new Niemann-Pick murine model | In contrast to WT littermates, cerebella from *Npc1I1061T* mice showed progressive, age-dependent loss of calbindin D staining,  predominantly in the anterior lobules, indicating loss of Purkinje cells. The progressive Purkinje cell degeneration was also reflected in the age-dependent reduction in calbindin D mRNA expression. In the cerebella of P63 and P105 mice, there was a significant loss of Purkinje cells in anterior (P63, 58% reduction; P105, 91% reduction, central (P105, 80% reduction), posterior (P105, 65% reduction), and nodular (P105, 41% reduction) zones but no appreciable Purkinje cell loss in lobule X, which is generally preserved even in end-stage disease Loss of Purkinje cell bodies was accompanied by axonal spheroid formation, dystrophic dendritic abnormalities, and microglial and astrocytic changes in affected cerebellar areas. Although the distribution of Purkinje cell loss in the *Npc1I1061T* mice was similar to that observed in the *Npc1*^_/_^ model, the degree of Purkinje cell loss at P63 was less severe than in similarly aged *Npc1*^_/_^ mice, findings consistent with delayed onset of impaired motor coordination in the knock-in compared with the knock-out model. |
| **(Ramirez et al., 2010)**  BALB/c-npc1^nih^  NPC1^–/–^  mouse model npc1^nih^ | Whole brain weight  Cerebellum (Purkinje cells number) | Niemann-Pick type C1 disease arises from a mutation inactivating NPC1 protein that normally moves unesterified cholesterol from the late endosomal/lysosomal complex of cells to the  cytosolic compartment for processing. As a result, cholesterol accumulates in every tissue of the  body causing liver, lung and central nervous system disease.  Cerebellar neurodegeneration | Animals were fed ad libitum a cereal-based, low-cholesterol (0.02% cholesterol, 4% total  fat, w/w) diet (no. 7001; Harland Teklad, Madison, WI) upon weaning. Groups of mice were administered a subcutaneous injection of a 20% (w/v, in saline) solution of 2-hydroxypropyl-β-cyclodextrin (Sigma; product H107), (4000 mg/kg body weight), during  the late dark phase (09:00 hours) at the scruff of the neck. Matching mice were also injected with saline alone to serve as controls. | Effect of weekly CYCLO administration on organ weights and various parameters of cholesterol metabolism in the *npc1*−*/*− mouse. Both *npc1+/+* and *npc1*−*/*− mice were treated subcutaneously, weekly with either saline or CYCLO (4,000 mg/kg), and then studied at 49 days of age.  Relative organ weight (% body weight wt) - brain weight:  *npc1+/+ - 2*  *npc1+/+ + saline – 2*  *npc1-/- - 2*  *npc1-/- +* CYCLO *– 2*  At 49 days of age, only about 25% of Purkinje cells remained in the untreated *npc1*−*/*− mice, and many of these appeared to be pyknotic and dying. CYCLO treatment slowed this rate of Purkinje cell loss (following CYCLO administration, nearly twice as many of these cells survived), still cell numbers were significantly reduced*.* Importantly, in animals that survived to 160 days of age, no Purkinje cells could be identified.  The numbers of Purkinje cells in the cerebellum were counted, and are expressed relative to the number  found in the *npc1+/+* mice treated with saline:  *npc1+/+ - 1.00*  *npc1+/+ + saline – 1.00*  *npc1-/- - 0.25*  *npc1-/- +* CYCLO *– 0.50* |
| **(Repa et al., 2007)**  BALB/c-npc1^nih^  NPC1^–/–^  mouse model npc1^nih^ | Whole-Brain weight  Cerebellum weight | Because disordered cholesterol metabolism  is a major part of this syndrome, the NPC mouse is an ideal model in which to explore the relationship between sterol movement  across the blood– brain barrier (BBB) and the progressive neurodegeneration characteristic of this disease. The present  studies, therefore, were undertaken to determine whether activation  of the target genes of LXR leads to a change in cholesterol balance across the brain and, additionally, whether this change affects the degree of nerve cell death.  Treatment with LXR agonist T1317 (transcription factors like the liver X receptor  (LXR)) | Mice were fed *ad libitum* a low-cholesterol mouse diet (no. 7001; Harlan Teklad, Madison, WI) containing 0.02% cholesterol, or this same diet supplemented with T0901317 (0.025% w/w; Cayman Chemical, Ann Arbor, MI). This compound  is hereafter abbreviated T1317. In one experiment, the basal diet was supplemented with 1% cholesterol. | Whole-Brain weight (mg)  NPC 1^+/+^( 42 or 56 d of age):  Without T1317 (430, 450 mg); with T1317 (430, 450 mg);  NPC 1^-/-^( 42 or 56 d of age):  Without T1317 (400, 400 mg); with T1317 (370, 330 mg);  Cerebellum weight (mg)  NPC 1^+/+^( 42 or 56 d of age):  Without T1317 (59, 59 mg); with T1317 (59, 58 mg);  NPC 1^-/-^( 42 or 56 d of age):  Without T1317 (51, 50 mg); with T1317 (49, 41 mg); |
| **(Schlegel et al., 2016)**  BALB/c-npc1^nih^  NPC1^–/–^  mouse model npc1^nih^ | Whole-Brain weight | Reduced Brain Weights after Miglustat- and Combination Treatment | Starting at postnatal day 7 (P7) and thenceforth, mice of the combi-group were injected weekly with 2-hydroxypropyl-cyclodextrin/allopregnanolone (25 mg/kg allopregnanolone dissolved in 40% 2-hydroxypropyl-cyclodextrin in Ringer’s solution, 4000 mg/kg, i.p., all from Sigma-Aldrich,  Munich, Germany). Additionally, these mice were daily injected with miglustat, dissolved in 0.9% NaCl  solution, 300 mg/kg i.p. (N-butyldeoxynojirimycin, Zavesca; Actelion Pharmaceuticals, San Francisco, CA, USA) from P10 to P23. From P23 onwards until termination of experiments mice were fed standard chow with embedded miglustat resulting in daily intake of 1200 mg/kg miglustat. The miglu-group  was treated like the combi-group, but without administration of cyclodextrin/allopregnanolone,  instead mice got vehicle. Mice of the sham-group were injected like those of the combi-group at the  various time points with the respective volumes of 0.9% NaCl or without volume and were fed with chaw without drugs. | The brains of combination- (mean: 0.408 g, SD: 0.0234 g) and miglustat-treated mice (mean: 0.426 g, SD: 0.0156 g) were significantly lighter compared to sham-treated mice (mean 0.449 g, SD: 0.0278 g). |
| **(Smith et al., 2009)**  BALBc/NPC^nih^ mice  were bred as heterozygotes to generate Npc1^−/−^ mice and control genotypes. | Effects of NSAIDS and miglustat on Purkinje cell survival in cerebellum | Purkinje cell loss in cerebellum  To test whether the treatments had any impact on brain inflammation. The animals were sacrificed at 7.4 weeks of age and the cerebella analysed for CD68 expression as a measure of  microglial activation. | Aspirin (Sigma, 200 g/kg/day) and ibuprofen (Sigma, 100 mg/  kg/day) were supplemented as dry admix to powdered RM1 mouse chow (SDS, UK) (starting at 6 weeks of age). Miglustat (600 mg/kg/  day, Oxford lycoSciences/Celltech, UK) was administered as dry  admix as above (starting at 3 weeks of age). The antioxidant L-ascorbic acid (vitamin C, 2.5% in powdered chow) was administered from 6 weeks of age. The untreated mice were fed on powdered chow. | The number of calbindin-labeled Purkinje cells in the crus1 zone was counted to assess the impact of drug treatment on Purkinje cell survival:  crux1 PCs /section  untreated NPC^−/−^ controls – 15  Ibuprofen – 15  Miglustat – 30  Miglustat + Ibuprofen - 30  The results indicate that ibuprofen did not have a protective effect on Purkinje cell survival, while miglustat treated animals had significantly more Purkinje cells than the untreated NPC^−/−^ controls.  All the treated Npc1^−/−^ animals presented with significantly less CD68+ cells in all areas of the cerebellum  compared to untreated Npc1^−/−^ animals.  A significant reduction in the number of CD68+ cells in ibuprofen treated mice was observed when compared to untreated Npc1^−/−^ mice, in the deep cerebellar nuclei (DCN; ibuprofen 390.6 cells/mm2; NPC^−/−^ 504.20 cells/mm2), the white matter and internal granular layer (WM and IGL; ibuprofen 237.72 cells/mm2; NPC^−/−^  328.06 cells/mm2) and the molecular layer (ML; ibuprofen 200.59 cells/mm2; NPC^−/−^ 314.58 cells/mm2). However, miglustat treatment reduced the levels of CD68+ cells by a significant margin, not only relative to untreated Npc1^−/−^ mice but also when  compared to the ibuprofen treated animals (DCN; ibuprofen 390.95 cells/mm2 vs miglustat 310.95 cells/mm2, pb0.05. WM  and IGL; ibuprofen 237.72 cells/mm2 vs miglustat 155.34 cells/ mm2, pb0.05. ML; ibuprofen 200.59 cells/mm2 vs miglustat  98.94 cells/mm2). There was no significant difference in  the number of CD68+ cells in WM and IGL or ML of miglustat treated mice compared to miglustat/ibuprofen co-treated mice, but a difference was observed in the DCN of these treatment groups (miglustat 310.95 cells/mm2 vs miglustat/ibuprofen  216.43 cells/mm2, p b0.01, compare panels G and J), thus indicating a modest but significant cooperative effect of miglustat and ibuprofen on NPC1 brain inflammation. |
| **(Tanaka et al., 1988)**  The sphingomyelinosis (spm) mouse was discovered as a new mutant in the C57BL/KsJ inbred strain in 1975 (Research Lab., Nippon Shinyaku Co., Ltd) | Cerebellar cortex  Cerebellum (vermis, hemisphepheres)  Atrophy of the Purkinje cells  Whole brain weight | Neuropathological investigation of sphingomyelinosis (spm) mice has shown them to be an authentic model of Niemann-Pick disease in humans.  The affected mice developed hepatosplenomegaly and abnormalities in motor coordination with tremor of the body and extremities, prominently manifested with progressive cerebellar sings.  Loss of Purkinje cells, which probably accounts for the clinical sings of cerebellar impairment, was statistically evaluated in the vermis and hemispheres. | The mutant strain of spm mice, in which clinicopatholoical features are very similar to those of human Niemann-Pick disease type C, may be an excellent model for elucidating the pathogenesis of lysosomal storage disorders and, to assess therapeutic trials. | In the mutant mice aged six through ten k there was a rapid decrease in number of Purkinje cells in both vermis and hemispheres.  At age 12 wk the brains of the mutant mice were obviously smaller in size, a mean weight being about two thirds that of controls, and the degree of brain atrophy was more remarkable in the cerebellum than in the cerebrum.  In the cerebellar cortex a diffuse and marked loss of Purkinje cells was noted in the mutants. The loss of the Purkinje cells seemed to be more conspicuous in the vermis than in the hemispheres. In some areas of the vermis, a severe loss of Purkinje cells and a swelling of Golgi cells were encounter. Occasional swollen axons were also seen in the granular layer. Until the age of five wk there were no significant differences in the number of Purkinje cells compared to the control. A sharp decline in the number of Purkinje cells started at the age of six wk before signs of cerebellar disease had appeared; this cellular loss reached its maximum extent at ten wk. After the ten wk, during the terminal period of the mutant mouse’s life span, only a few Purkinje cells survived.  Number of Purkinje cells:  Spingomyelinosis mice (A):  Vermis: 4 wk – 51.3 ± 6.0, 12 wk – 1.0 ± 1.7  Hemisphere: 4 wk – 46.9 ± 5.5, 12 wk – 1.2 ±1.9  Controls (C):  Vermis: 4 wk – 51.5 ± 6.5, 12 wk – 38.5 ± 2.4  Hemisphere: 4 wk – 46.0 ± 5.2, 12 wk – 37.1 ±5.4  Ratio (A/C):  4 wk: vermis – 99.6%; hemisphere – 101.9%  12 wk: vermis – 2.6%; hemisphere – 3,2% |
| **(Williams et al., 2014)**  BALBc/NPC^nih^ mice were bred as heterozygotes to generate Npc1−/−  mice and control genotypes. | Purkinje cell survival/neuroprotection in the NPC1−/− cerebellum.  Purkinje cell survival was quantified for each therapy, recording the number of surviving lobule III Purkinje cells/mm of Purkinje  cell layer.  Effect of combination therapies on microglial recruitment in the NPC1−/− cerebellum. | The untreated  Npc1^−/−^ mice displayed the characteristic progressive loss of  Purkinje cells with large-scale loss from lobules I–IV by 7.5 weeks. | Ibuprofen (Sigma, 100 mg/kg/day) was supplemented as a dry admixture to powdered RM1 mouse chow (SDS, UK) (from 6 weeks of age). Miglustat (600 mg/kg/day, Oxford GlycoSciences/Celltech, UK) and curcumin (Sigma, 150 mg/kg/day) were administered as dry admixtures as above (from 3 weeks of age). The untreated mice were fed on powdered chow (n=34). Treatment groups were made up of approximately equal numbers of males and females and received ibuprofen (n= 14), curcumin (n = 11), miglustat (n = 11), curcumin and ibuprofen (n = 5), curcumin and miglustat (n = 11), or all three therapies  (n = 9). | There was little survival of Purkinje cells in this region in untreated Npc1−/− cerebellum, with the few remaining cells (2.54 Purkinje cells/mm pcl) exhibiting dendritic degeneration. A similar level of neurodegeneration was evident in the ibuprofen-treated cerebellum, where a small but non-significant increase in Purkinje cells was observed (4.53 Purkinje cells/mm pcl). Curcumin treated mice demonstrated a degree of Purkinje cell protection relative to untreated controls (7.94 Purkinje cells/mm pcl), as did the curcumin  & ibuprofen dual therapy treated mice (7.10 Purkinje cells/mm pcl).  Miglustatmonotherapy exhibited a much stronger neuroprotective effect than the curcumin treatment, and while patches of Purkinje cell loss were still evident, the level of Purkinje cell survival was much  higher (15.87 Purkinje cells/mm pcl). The same was true for the curcumin & miglustat dual treatment (16.00 Purkinje cells/mm pcl), and although the dual treatment did have slightly more Purkinje cell survival (and objectively slightly better dendritic condition), it was not significantly better than miglustat alone. Remarkably, the triple combination therapy resulted in a high level of neuroprotection, with only sporadic patches of Purkinje cell loss evident across lobule III (21.20 Purkinje cells/mm pcl) This was a slight but significant improvement over the other miglustat-containing therapie, indicating that the triple combination therapy was the most effective neuroprotective treatment tested.  In ibuprofen-treated mice, microglial recruitment to the molecular layer was reduced from 1027.98 microglia/mm2 observed in untreated Npc1^−/−^ mice to 492.89 microglia/mm2, indicating a strong anti-inflammatory effect, while curcumin monotherapy exhibited no anti-inflammatory properties. Combining ibuprofen with curcumin did reduce microgliosis when compared to curcumin alone  (765.84 microglia/mm2). Miglustat monotherapy (579.84 microglia/mm2) and miglustat & curcumin dual therapy (497.67 microglia/mm2) both significantly reduced microgliosis compared to ibuprofen & curcumin therapy.  The greatest reduction in microgliosis was in the triple combination group, where only 337.38 microglia/mm2 of lobule II molecular  Layer were observed, significantly less than the curcumin & miglustat. |
| **(Xie et al., 1999)**  BALB/c-npc1^nih^  NPC1^–/–^  mouse model npc1^nih^ | Whole brain weight | As in the human, these animals also show marked demyelination of the brain, rapid loss of cerebellar Purkinje cells, and polymorphous cytoplasmic bodies in various neurons. | After weaning, the animals were maintained on a pelleted, basal rodent diet (No. 7001, Harlan Teklad, Madison, WI) that had a cholesterol content of 0.016% (wt/wt) and a total lipid content of 5% (wt/wt). In one experiment, animals were fed for 1 week a meal form of this basal diet, to which was added 0.4% (wt/wt) cholesterol. | These measurements were carried out in 7-week-old mice:  Animal and organ weights, cholesterol pool sizes, and rates of dietary cholesterol absorption in the NPC+/+/LDLR+/+ and NPC-/-/LDLR+/+ mice used in these studies  Weight of brain (g):  NPC+/+ - 0.44 ± 0.01  NPC-/- - 0.38 ± 0.03*  An asterisk (*) identifies those values in the NPC-/-/LDLR+/+ mice that were significantly different from those in the control NPC+/+/LDLR+/+ animals. |
| **(Yamada et al., 2001)**  A colony of the C57BL/KsJ-npc1spm mice was maintained  by interbreeding of heterozygous mice or by that of  heterozygous males (1 /spm) with normal females who received transplantation of ovaries from homozygous  females (spm/spm). | Purkinje cells in ventral posterial lateral (VPL) and medial (VPM) nuclei of thalamus.  The number of neurons in the ventral lateral thalamic nuclei, cerebral cortex, hippocampus CA3 regions and  gigantocellular reticular nucleus of the brain stem (data not shown). | Niemann±Pick disease type C (NP-C) disease is a progressive and fatal neurological disorder characterized by accumulation of cholesterol and glycosphingolipids in peripheral tissues and that of glycosphingolipids in the brain. A C57BL/KsJ-npc1spm mutant strain is a genetically authentic model of NP-C. This study investigated neuronal cell loss and lipid accumulation in the npc1spm mouse brain. | No treatment, this study investigated neuronal cell loss and lipid accumulation in the npc1spm mouse brain. | Nissl-staining revealed abundant swollen neurons in the neocortex, piriform cortex, hippocampus and basal ganglia at 3±4 wk of age. In addition to loss of the Purkinje cells, we found a conspicuous cell loss in the ventral posterial lateral (VPL) and medial (VPM) nuclei of thalamus, which became apparent after 4±5 wk.  Purkinje cells began to disappear at 5±6 wk-old and were almost gone by 8±9 wk whereas degeneration  of thalamic neurons started by 4±5 wk-old and progressed gradually up to 12±13 wk. Age-dependent progression of neuronal cell loss in the cerebellar Purkinje layer, VPL nucleus, VPM nucleus and ventral lateral nucleus of the thalamus was shown.  They also evaluated  the number of neurons in the ventral lateral thalamic nuclei, cerebral cortex, hippocampus CA3 regions and  gigantocellular reticular nucleus of the brain stem (data not  shown) in normal and homozygous mice did not detect any  significant neuronal loss in these areas. |
| **(Zervas et al., 2001)**  The BALBc/NPC^nih^  The BALBc/NPC^nih^ mice used to establish a breeding colony were obtained from Dr. Peter Pentchev at the NIH (Bethesda, Maryland, USA), while cats with NPC were from a colony of animals maintained at Colorado State University. | Cerebellum | To evaluate the effectiveness of *N*B-DNJ therapy on cerebellar pathology, we used antibodies to Parvalbumin and Calbindin, respec tively, to evaluate axonal spheroid formation and Purkinje cell loss. | We provided *N*B-DNJ (*N*-butyldeoxynojirimycin (*N*B-DNJ), an inhibitor of glucosylceramide synthase mixed in ground chow to mice (1200 mg/kg/day, begin ning at 3.5 weeks of age) and by direct oral administration twice daily to cats. We initiated treatment of the first NPC cat at 21 weeks of age and administered DNJ for 54 days over an 84 day period, during which time  the dose was tapered from 150 mg/kg/day to 50 mg/kg/day. | In wild-type mice and cats, Parvalbumin-immunoreactivity  was detected in Purkinje cells and in the molecular layer, while Calbindin staining was observed in Purkinje cells and their dendrites. In feline  NPC disease, we previously found that axonal spheroids initially appeared within the neuropil of deep cerebellar  nuclei and adjacent white matter and later spread proximally toward Purkinje cell somata prior to the death of these neuron. Purkinje cell axonal spheroids and death correlate with worsening motor system dysfunction  in both the cat and mouse models. Consistent with these earlier studies, untreated 9- to 10-week-old  NPC mice in the present study displayed an extensive number of Parvalbumin-positive axonal spheroids. In contrast, *N*B-DNJ-treated NPC mice at this same age showed diminished spheroid formation in the granule  cell layer and preserved Purkinje cell architecture to varying degrees throughout the extent of cerebellar folia. Calbindin staining of untreated NPC mice revealed a nearly complete absence of Purkinje cells in many areas of the cerebellum, while NPC mice treated with *N*B-DNJ exhibited persistence of many of these neurons. |

**References:**

Baudry, M., Yao, Y., Simmons, D., Liu, J., and Bi, X. (2003). Postnatal development of inflammation in a murine model of Niemann-Pick type C disease: immunohistochemical observations of microglia and astroglia. *Exp Neurol* 184(2)**,** 887-903. doi: 10.1016/S0014-4886(03)00345-5.

Beltroy, E.P., Richardson, J.A., Horton, J.D., Turley, S.D., and Dietschy, J.M. (2005). Cholesterol accumulation and liver cell death in mice with Niemann-Pick type C disease. *Hepatology* 42(4)**,** 886-893. doi: 10.1002/hep.20868.

Byun, K., Kim, D., Bayarsaikhan, E., Oh, J., Kim, J., Kwak, G., et al. (2013). Changes of calcium binding proteins, c-Fos and COX in hippocampal formation and cerebellum of Niemann-Pick, type C mouse. *J Chem Neuroanat* 52**,** 1-8. doi: 10.1016/j.jchemneu.2013.04.006.

Byun, K., Kim, J.M., Kim, N., Kang, J.A., Won, M.H., Jeong, G.B., et al. (2011). Alteration of the CNS pathway to the hippocampus in a mouse model of Niemann-Pick, type C disease. *J Chem Neuroanat* 42(1)**,** 39-44. doi: 10.1016/j.jchemneu.2011.04.003.

Cabeza, C., Figueroa, A., Lazo, O.M., Galleguillos, C., Pissani, C., Klein, A., et al. (2012). Cholinergic abnormalities, endosomal alterations and up-regulation of nerve growth factor signaling in Niemann-Pick type C disease. *Mol Neurodegener* 7**,** 11. doi: 10.1186/1750-1326-7-11.

Chandler, R.J., Williams, I.M., Gibson, A.L., Davidson, C.D., Incao, A.A., Hubbard, B.T., et al. (2017). Systemic AAV9 gene therapy improves the lifespan of mice with Niemann-Pick disease, type C1. *Hum Mol Genet* 26(1)**,** 52-64. doi: 10.1093/hmg/ddw367.

German, D.C., Quintero, E.M., Liang, C.L., Ng, B., Punia, S., Xie, C., and Dietschy, J.M. (2001). Selective neurodegeneration, without neurofibrillary tangles, in a mouse model of Niemann-Pick C disease. *J Comp Neurol* 433(3)**,** 415-425. doi: 10.1002/cne.1149.

Griffin, L.D., Gong, W., Verot, L., and Mellon, S.H. (2004). Niemann-Pick type C disease involves disrupted neurosteroidogenesis and responds to allopregnanolone. *Nat Med* 10(7)**,** 704-711. doi: 10.1038/nm1073.

Ko, D.C., Milenkovic, L., Beier, S.M., Manuel, H., Buchanan, J., and Scott, M.P. (2005). Cell-autonomous death of cerebellar purkinje neurons with autophagy in Niemann-Pick type C disease. *PLoS Genet* 1(1)**,** 81-95. doi: 10.1371/journal.pgen.0010007.

Li, H., Repa, J.J., Valasek, M.A., Beltroy, E.P., Turley, S.D., German, D.C., and Dietschy, J.M. (2005). Molecular, anatomical, and biochemical events associated with neurodegeneration in mice with Niemann-Pick type C disease. *J Neuropathol Exp Neurol* 64(4)**,** 323-333. doi: 10.1093/jnen/64.4.323.

Liu, B., Turley, S.D., Burns, D.K., Miller, A.M., Repa, J.J., and Dietschy, J.M. (2009). Reversal of defective lysosomal transport in NPC disease ameliorates liver dysfunction and neurodegeneration in the npc1-/- mouse. *Proc Natl Acad Sci U S A* 106(7)**,** 2377-2382. doi: 10.1073/pnas.0810895106.

Lopez, M.E., Klein, A.D., Hong, J., Dimbil, U.J., and Scott, M.P. (2012). Neuronal and epithelial cell rescue resolves chronic systemic inflammation in the lipid storage disorder Niemann-Pick C. *Hum Mol Genet* 21(13)**,** 2946-2960. doi: 10.1093/hmg/dds126.

Luan, Z., Saito, Y., Miyata, H., Ohama, E., Ninomiya, H., and Ohno, K. (2008). Brainstem neuropathology in a mouse model of Niemann-Pick disease type C. *J Neurol Sci* 268(1-2)**,** 108-116. doi: 10.1016/j.jns.2007.11.018.

Maass, F., Petersen, J., Hovakimyan, M., Schmitt, O., Witt, M., Hawlitschka, A., et al. (2015). Reduced cerebellar neurodegeneration after combined therapy with cyclodextrin/allopregnanolone and miglustat in NPC1: a mouse model of Niemann-Pick type C1 disease. *J Neurosci Res* 93(3)**,** 433-442. doi: 10.1002/jnr.23509.

Ohara, S., Ukita, Y., Ninomiya, H., and Ohno, K. (2004). Degeneration of cholecystokinin-immunoreactive afferents to the VPL thalamus in a mouse model of Niemann-Pick disease type C. *Brain Res* 1022(1-2)**,** 244-246. doi: 10.1016/j.brainres.2004.06.037.

Praggastis, M., Tortelli, B., Zhang, J., Fujiwara, H., Sidhu, R., Chacko, A., et al. (2015). A murine Niemann-Pick C1 I1061T knock-in model recapitulates the pathological features of the most prevalent human disease allele. *J Neurosci* 35(21)**,** 8091-8106. doi: 10.1523/JNEUROSCI.4173-14.2015.

Ramirez, C.M., Liu, B., Taylor, A.M., Repa, J.J., Burns, D.K., Weinberg, A.G., et al. (2010). Weekly cyclodextrin administration normalizes cholesterol metabolism in nearly every organ of the Niemann-Pick type C1 mouse and markedly prolongs life. *Pediatr Res* 68(4)**,** 309-315. doi: 10.1203/PDR.0b013e3181ee4dd2.

Repa, J.J., Li, H., Frank-Cannon, T.C., Valasek, M.A., Turley, S.D., Tansey, M.G., and Dietschy, J.M. (2007). Liver X receptor activation enhances cholesterol loss from the brain, decreases neuroinflammation, and increases survival of the NPC1 mouse. *J Neurosci* 27(52)**,** 14470-14480. doi: 10.1523/JNEUROSCI.4823-07.2007.

Schlegel, V., Thieme, M., Holzmann, C., Witt, M., Grittner, U., Rolfs, A., and Wree, A. (2016). Pharmacologic Treatment Assigned for Niemann Pick Type C1 Disease Partly Changes Behavioral Traits in Wild-Type Mice. *Int J Mol Sci* 17(11). doi: 10.3390/ijms17111866.

Smith, D., Wallom, K.L., Williams, I.M., Jeyakumar, M., and Platt, F.M. (2009). Beneficial effects of anti-inflammatory therapy in a mouse model of Niemann-Pick disease type C1. *Neurobiol Dis* 36(2)**,** 242-251. doi: 10.1016/j.nbd.2009.07.010.

Tanaka, J., Nakamura, H., and Miyawaki, S. (1988). Cerebellar involvement in murine sphingomyelinosis: a new model of Niemann-Pick disease. *J Neuropathol Exp Neurol* 47(3)**,** 291-300. doi: 10.1097/00005072-198805000-00008.

Williams, I.M., Wallom, K.L., Smith, D.A., Al Eisa, N., Smith, C., and Platt, F.M. (2014). Improved neuroprotection using miglustat, curcumin and ibuprofen as a triple combination therapy in Niemann-Pick disease type C1 mice. *Neurobiol Dis* 67**,** 9-17. doi: 10.1016/j.nbd.2014.03.001.

Xie, C., Turley, S.D., and Dietschy, J.M. (1999). Cholesterol accumulation in tissues of the Niemann-pick type C mouse is determined by the rate of lipoprotein-cholesterol uptake through the coated-pit pathway in each organ. *Proc Natl Acad Sci U S A* 96(21)**,** 11992-11997. doi: 10.1073/pnas.96.21.11992.

Yamada, A., Saji, M., Ukita, Y., Shinoda, Y., Taniguchi, M., Higaki, K., et al. (2001). Progressive neuronal loss in the ventral posterior lateral and medial nuclei of thalamus in Niemann-Pick disease type C mouse brain. *Brain Dev* 23(5)**,** 288-297. doi: 10.1016/s0387-7604(01)00209-1.

Zervas, M., Dobrenis, K., and Walkley, S.U. (2001). Neurons in Niemann-Pick disease type C accumulate gangliosides as well as unesterified cholesterol and undergo dendritic and axonal alterations. *J Neuropathol Exp Neurol* 60(1)**,** 49-64. doi: 10.1093/jnen/60.1.49.
