# Supplementary figures and images for: Differently increased volumes of multiple brain areas in Npc1 mutant mice following various drug treatments
Source: Front Neuroanat. 2024 Jul 16;18:1430790. doi: 10.3389/fnana.2024.1430790 (PMC11286580; doi:10.3389/fnana.2024.1430790)

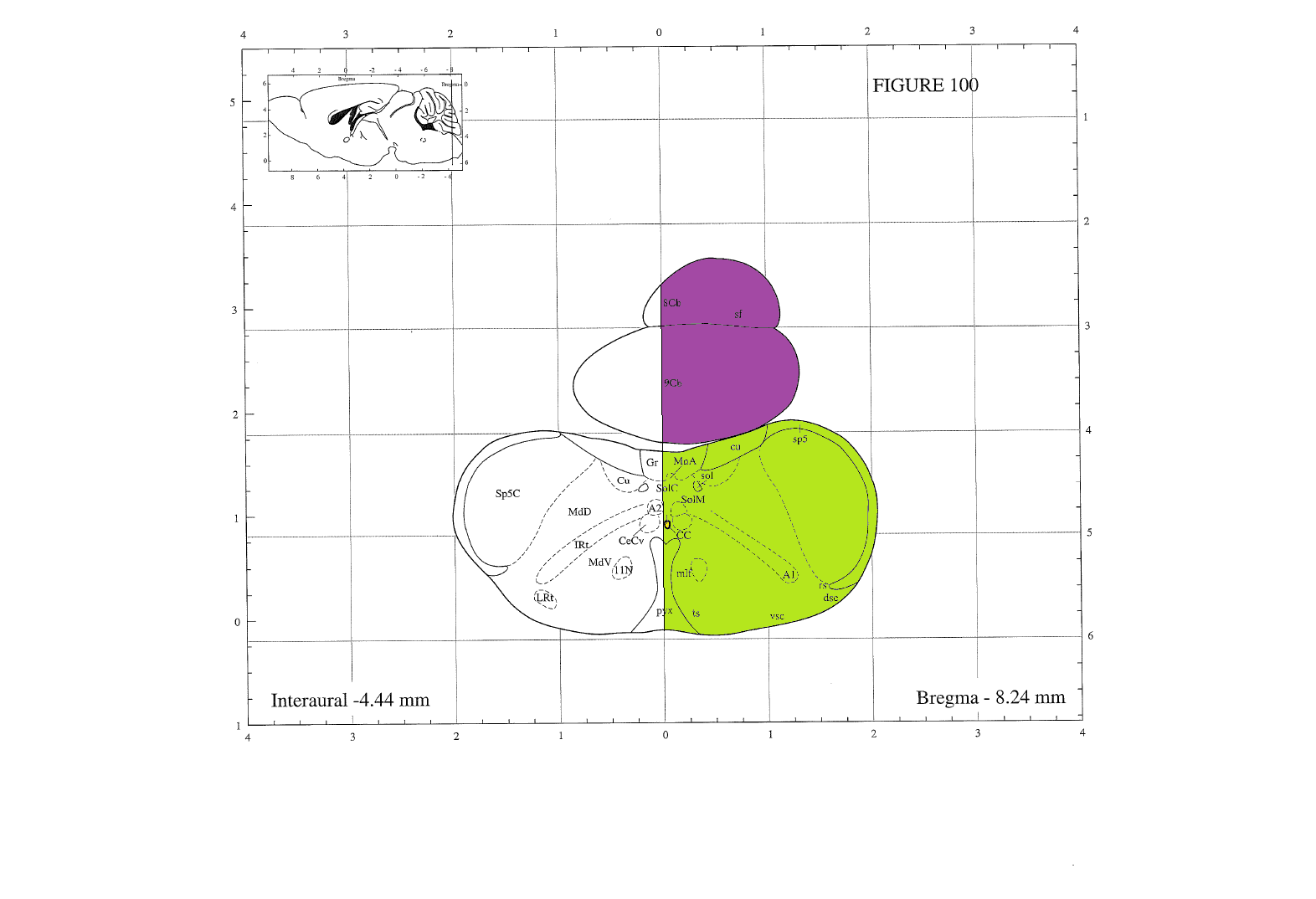

Supplement: Supplementary file 1 [file Data_Sheet_1.ZIP › Fig. S22.png]

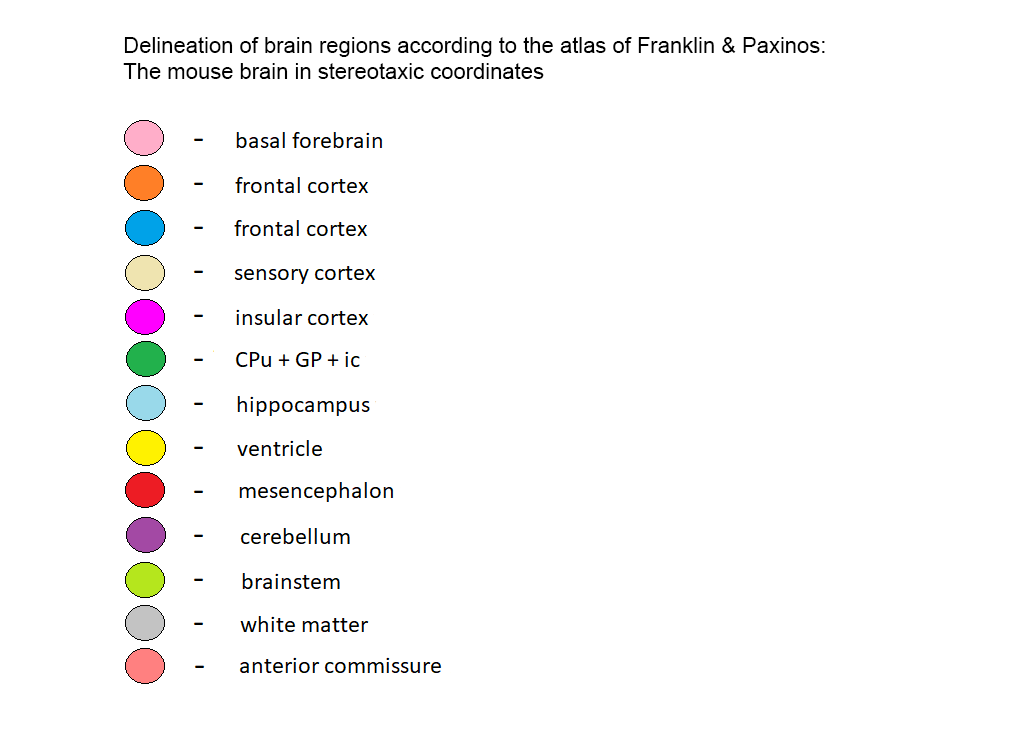

Supplement: Supplementary file 1 [file Data_Sheet_1.ZIP › Fig. S01.png]

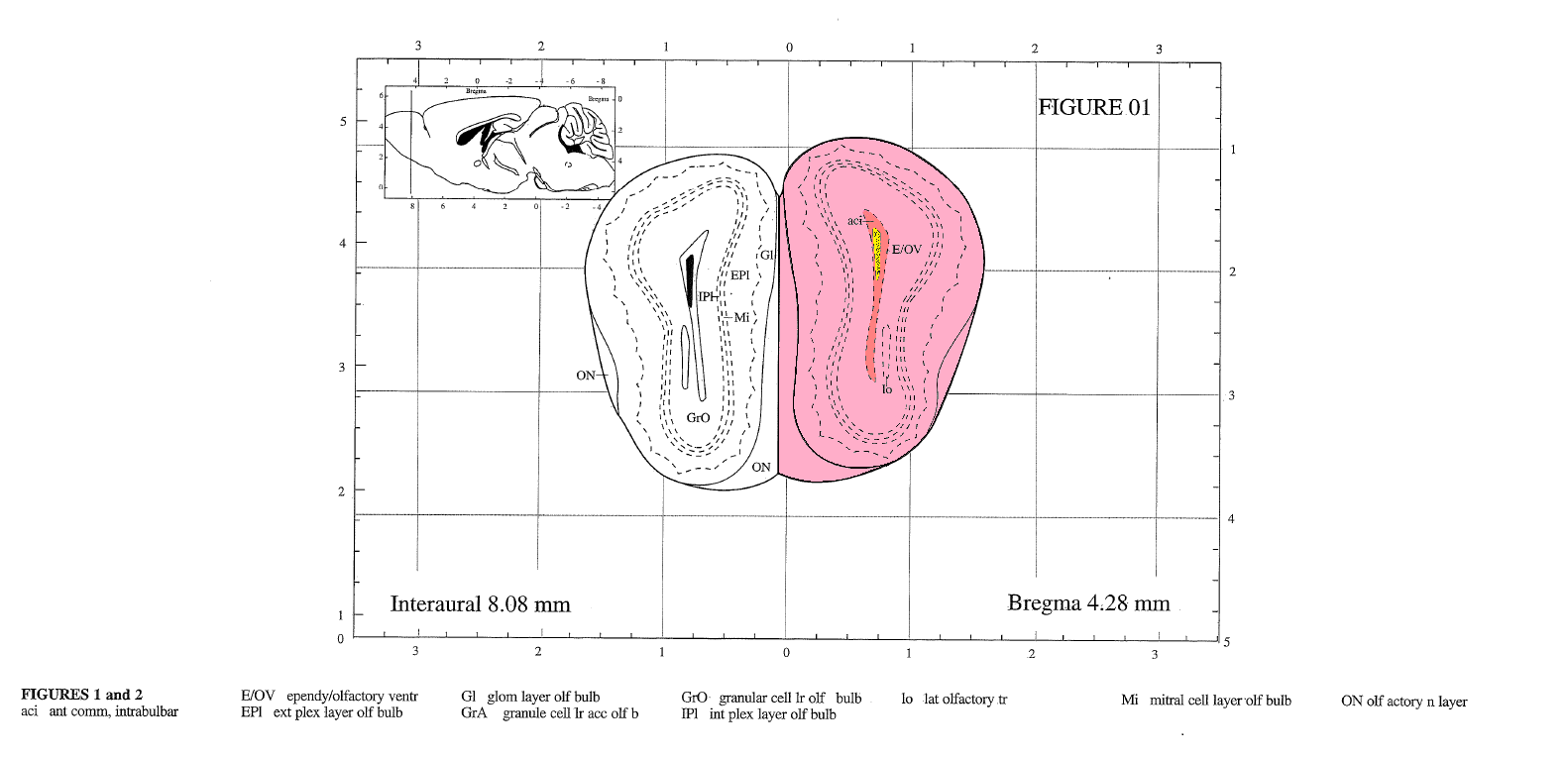

Supplement: Supplementary file 1 [file Data_Sheet_1.ZIP › Fig. S02.png]

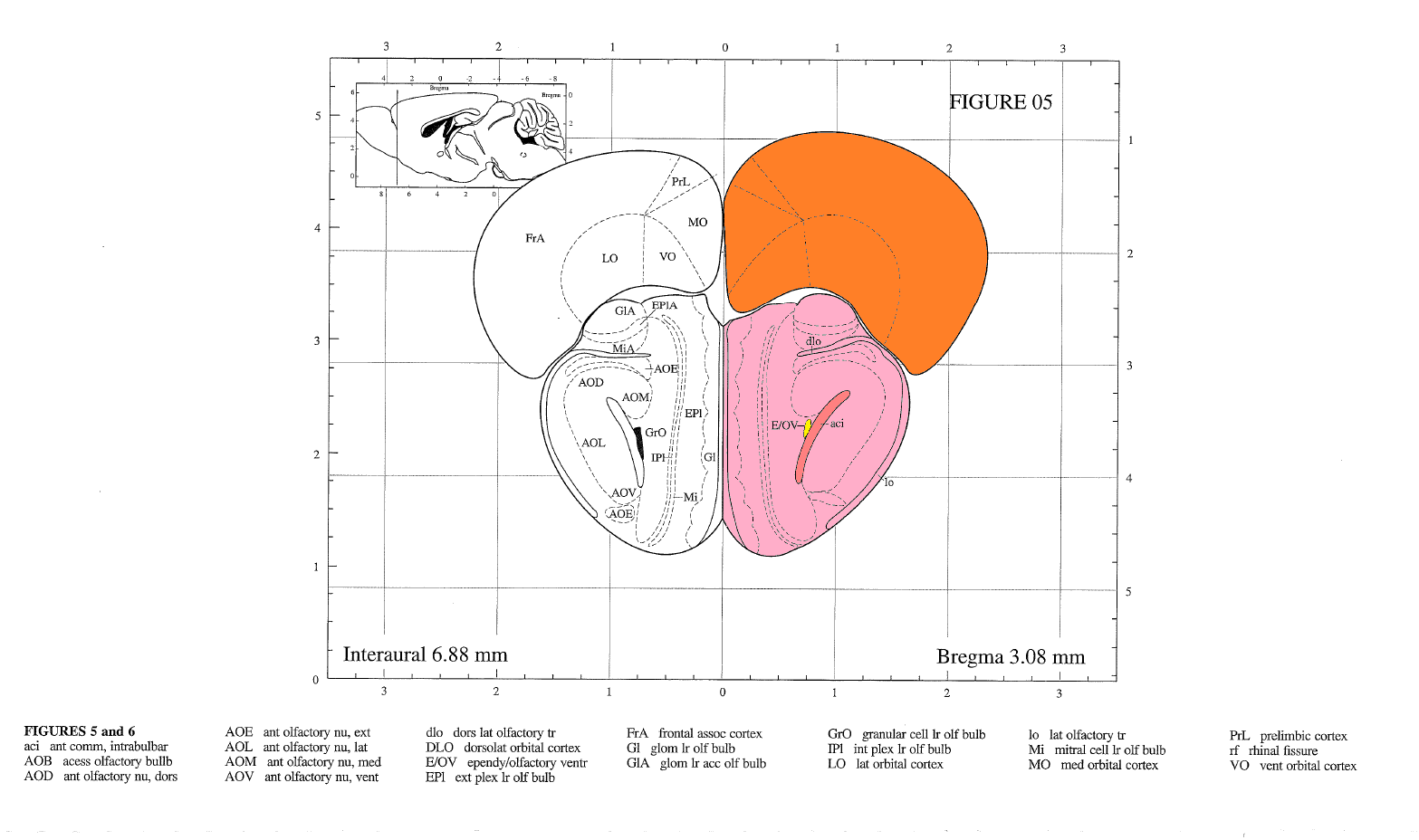

Supplement: Supplementary file 1 [file Data_Sheet_1.ZIP › Fig. S03.png]

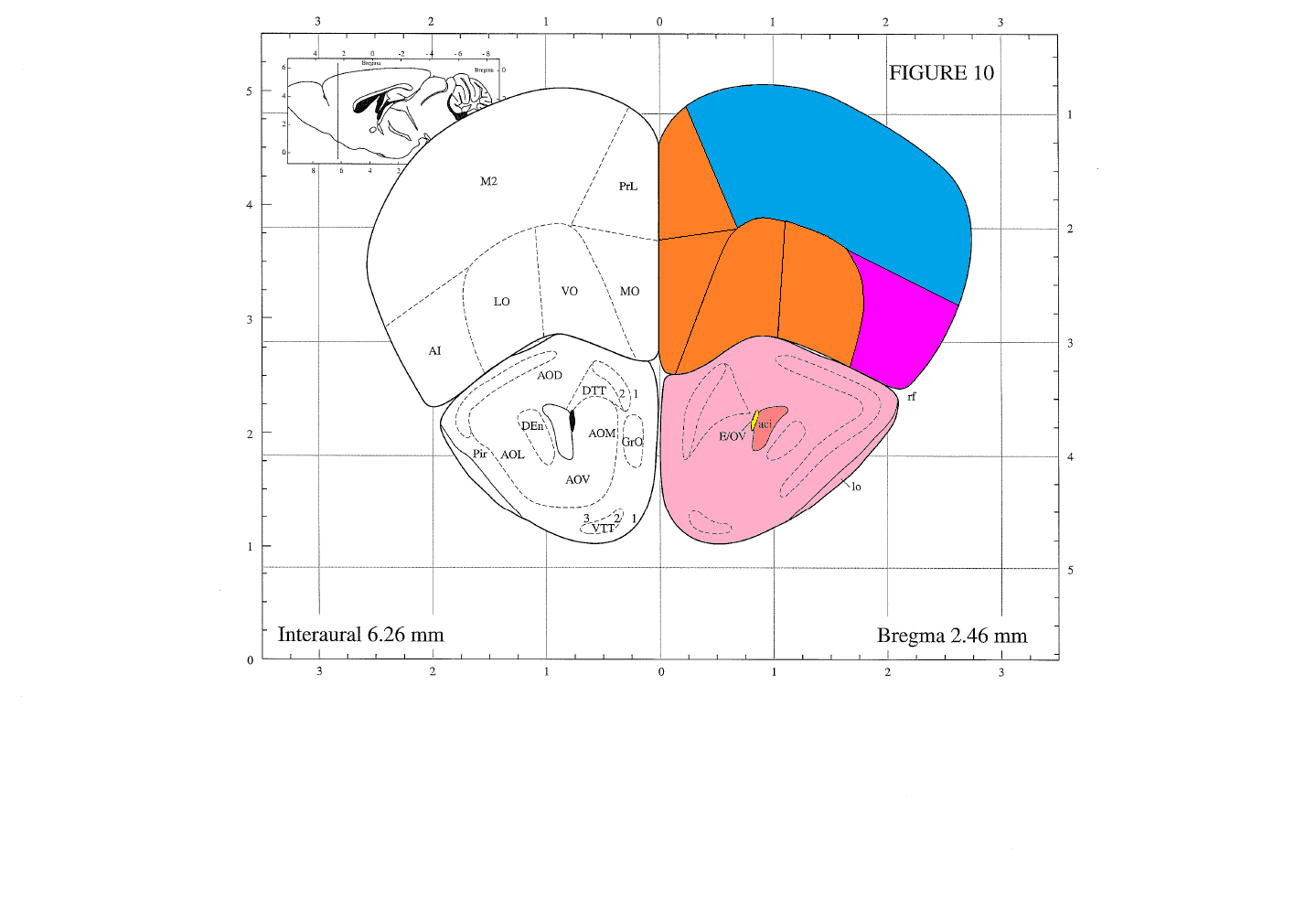

Supplement: Supplementary file 1 [file Data_Sheet_1.ZIP › Fig. S04.png]

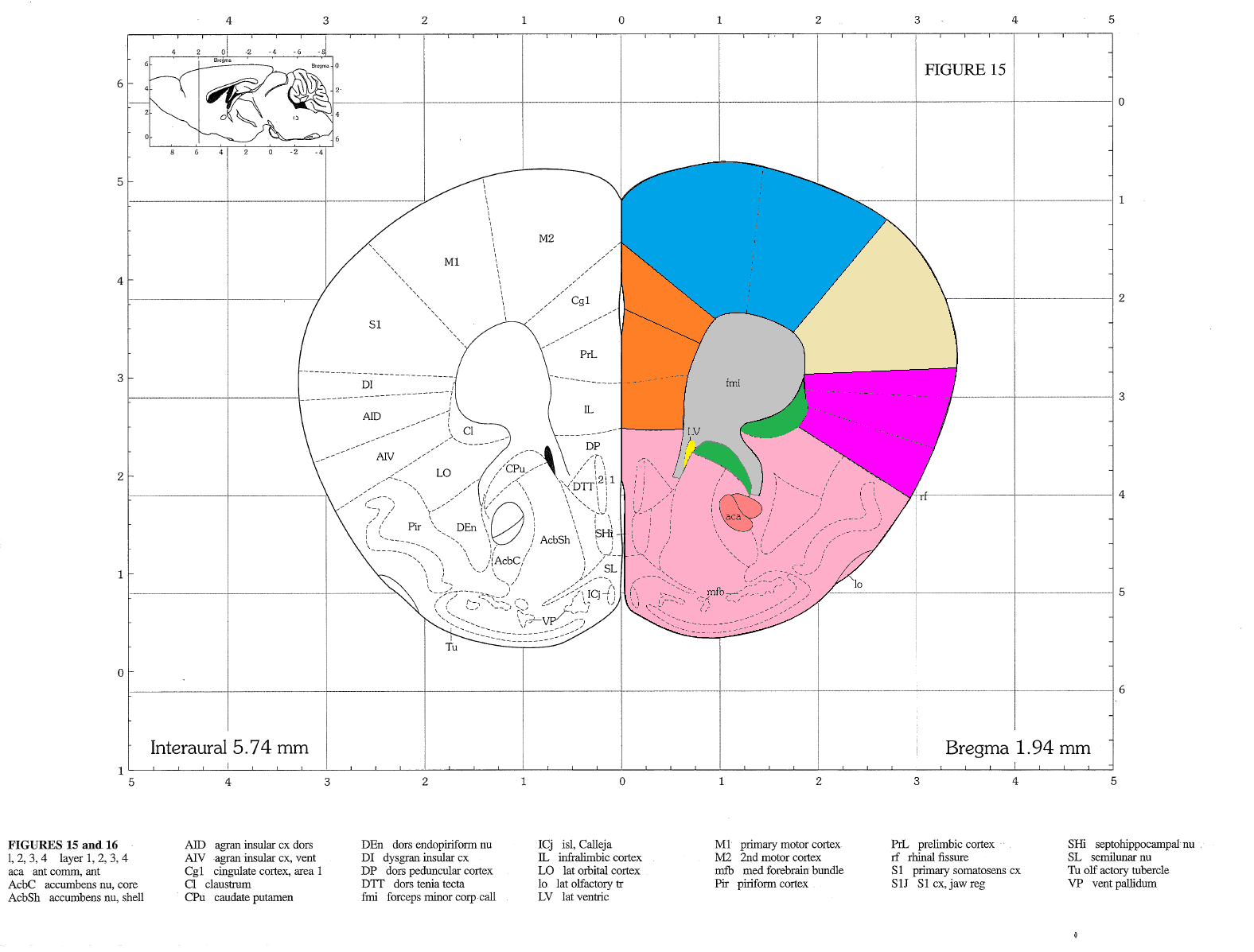

Supplement: Supplementary file 1 [file Data_Sheet_1.ZIP › Fig. S05.png]

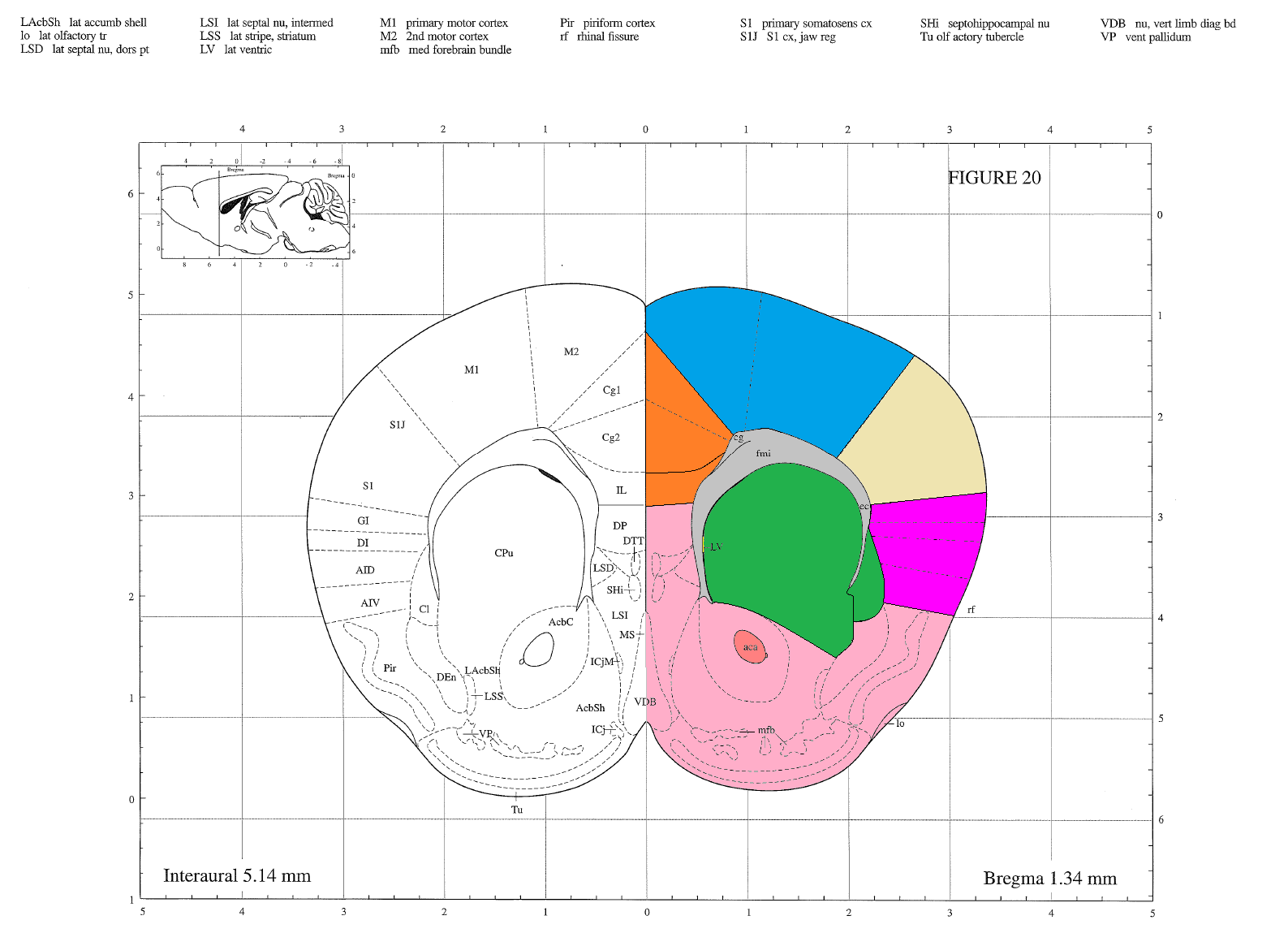

Supplement: Supplementary file 1 [file Data_Sheet_1.ZIP › Fig. S06.png]

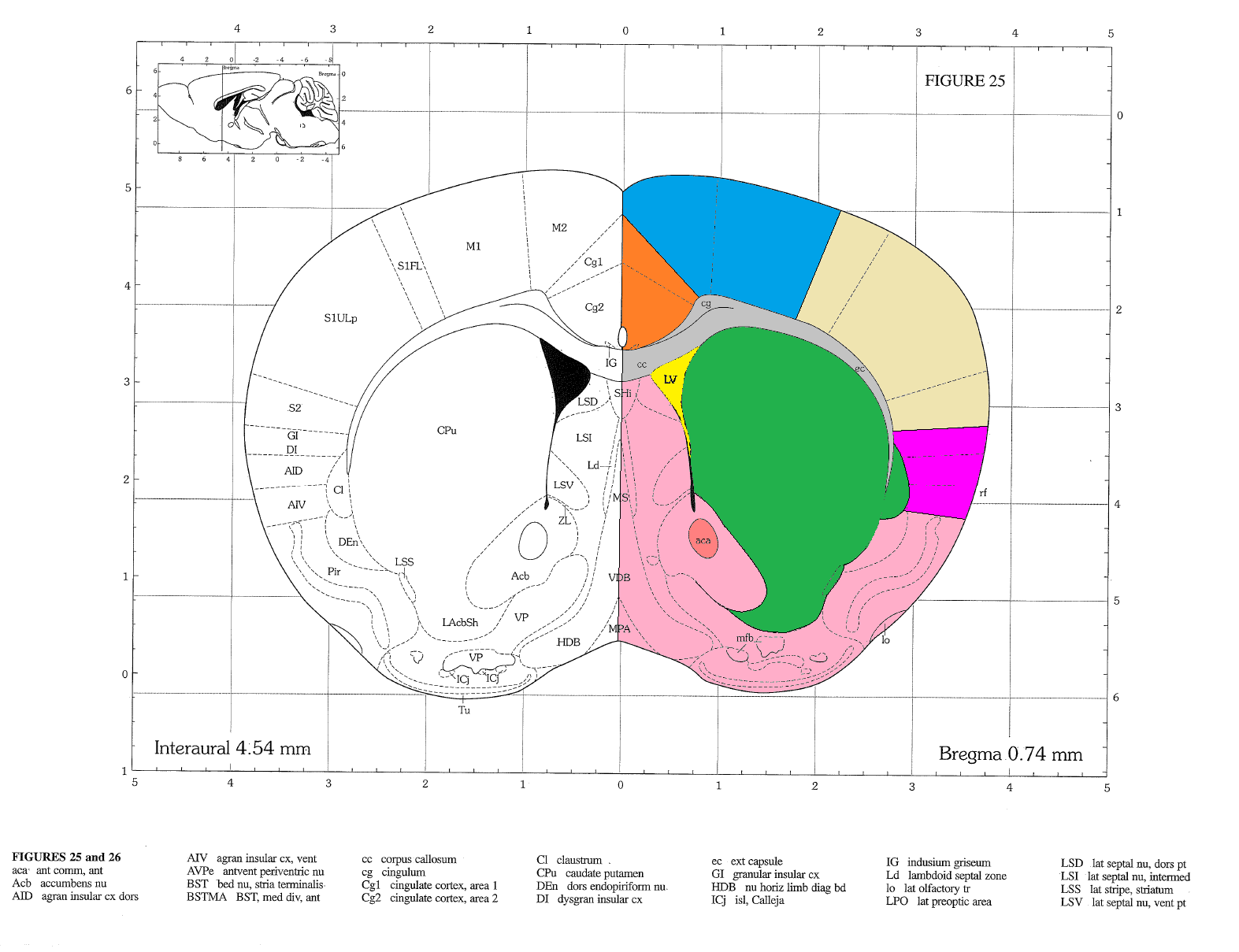

Supplement: Supplementary file 1 [file Data_Sheet_1.ZIP › Fig. S07.png]

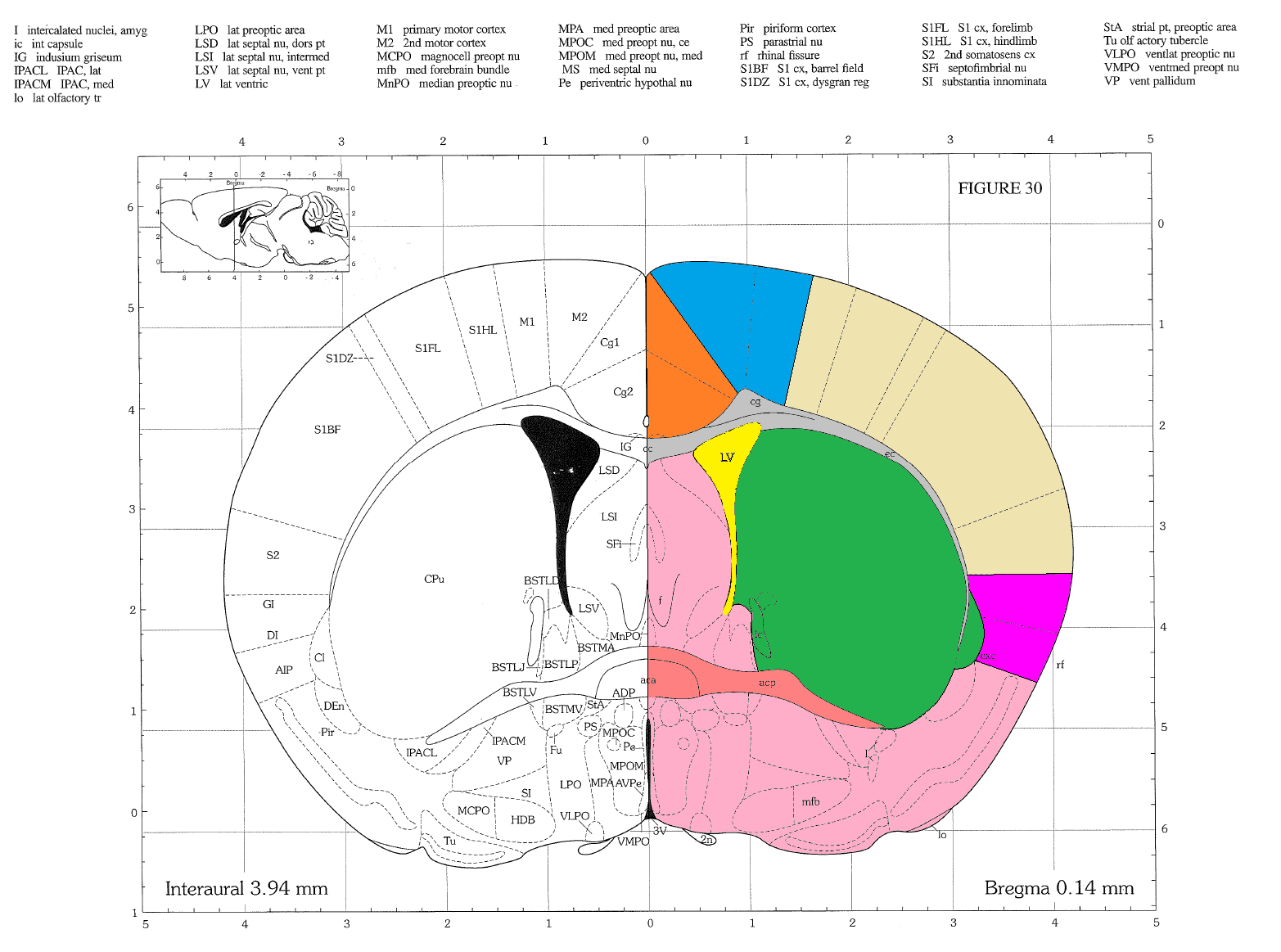

Supplement: Supplementary file 1 [file Data_Sheet_1.ZIP › Fig. S08.png]

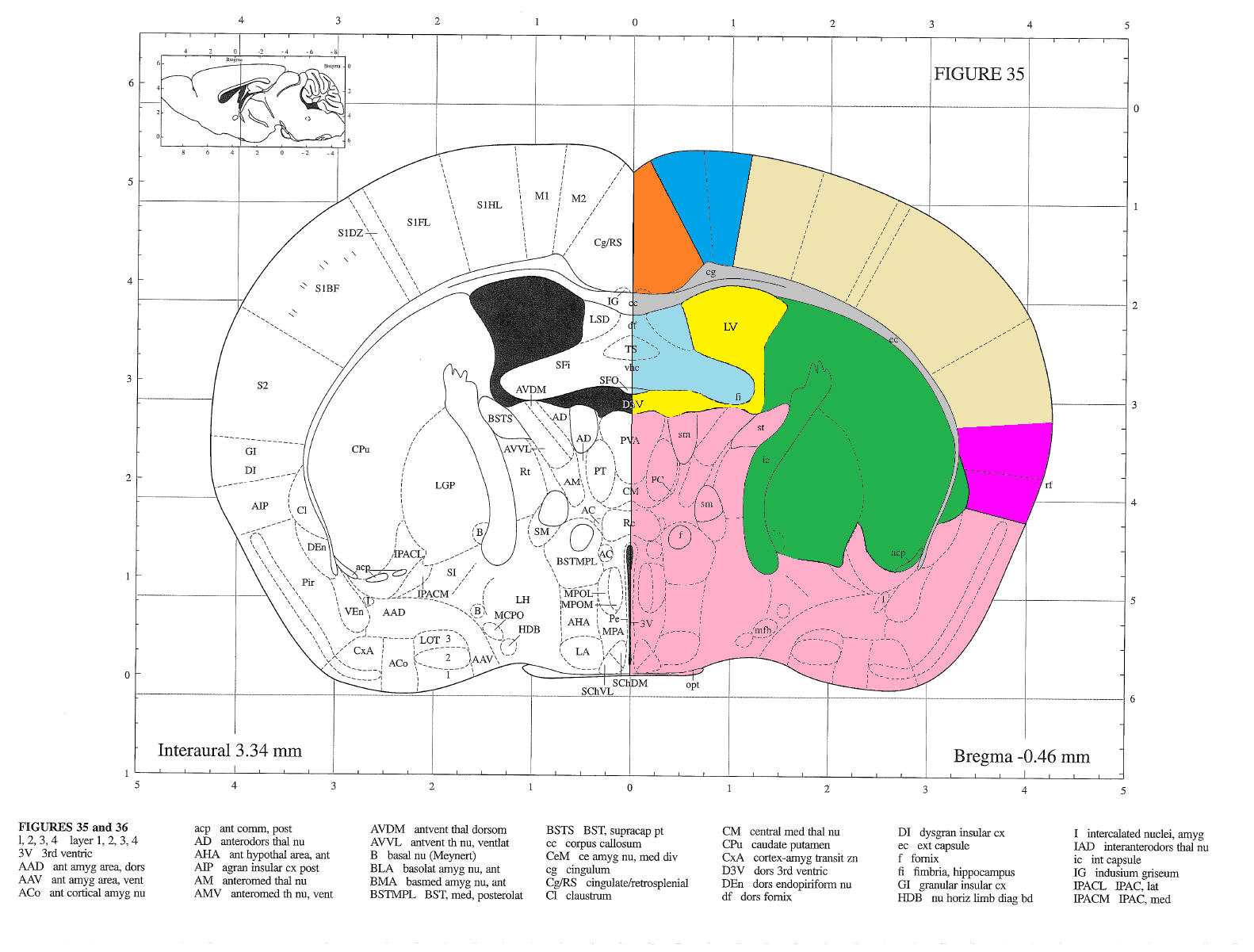

Supplement: Supplementary file 1 [file Data_Sheet_1.ZIP › Fig. S09.png]

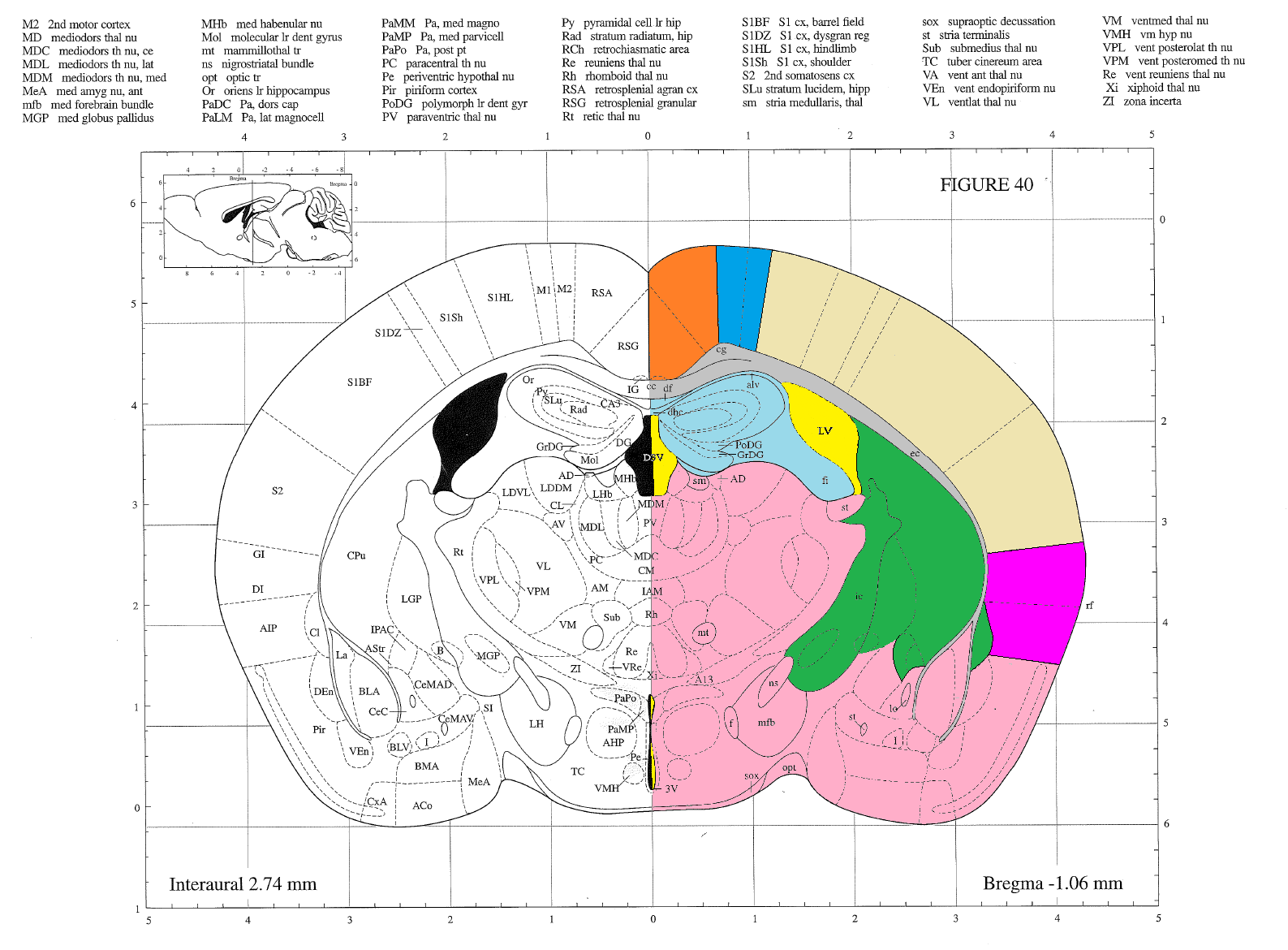

Supplement: Supplementary file 1 [file Data_Sheet_1.ZIP › Fig. S10.png]

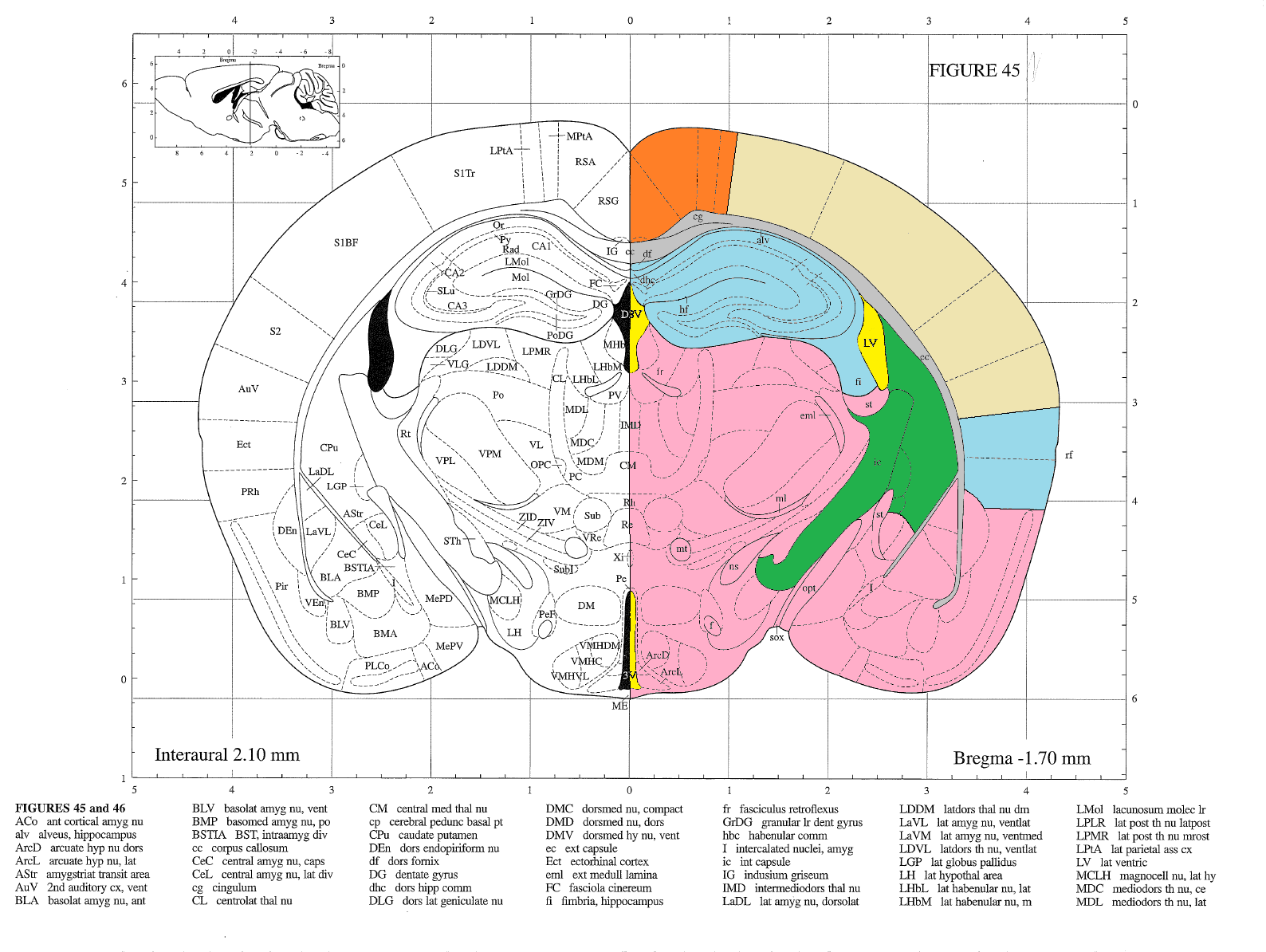

Supplement: Supplementary file 1 [file Data_Sheet_1.ZIP › Fig. S11.PNG]

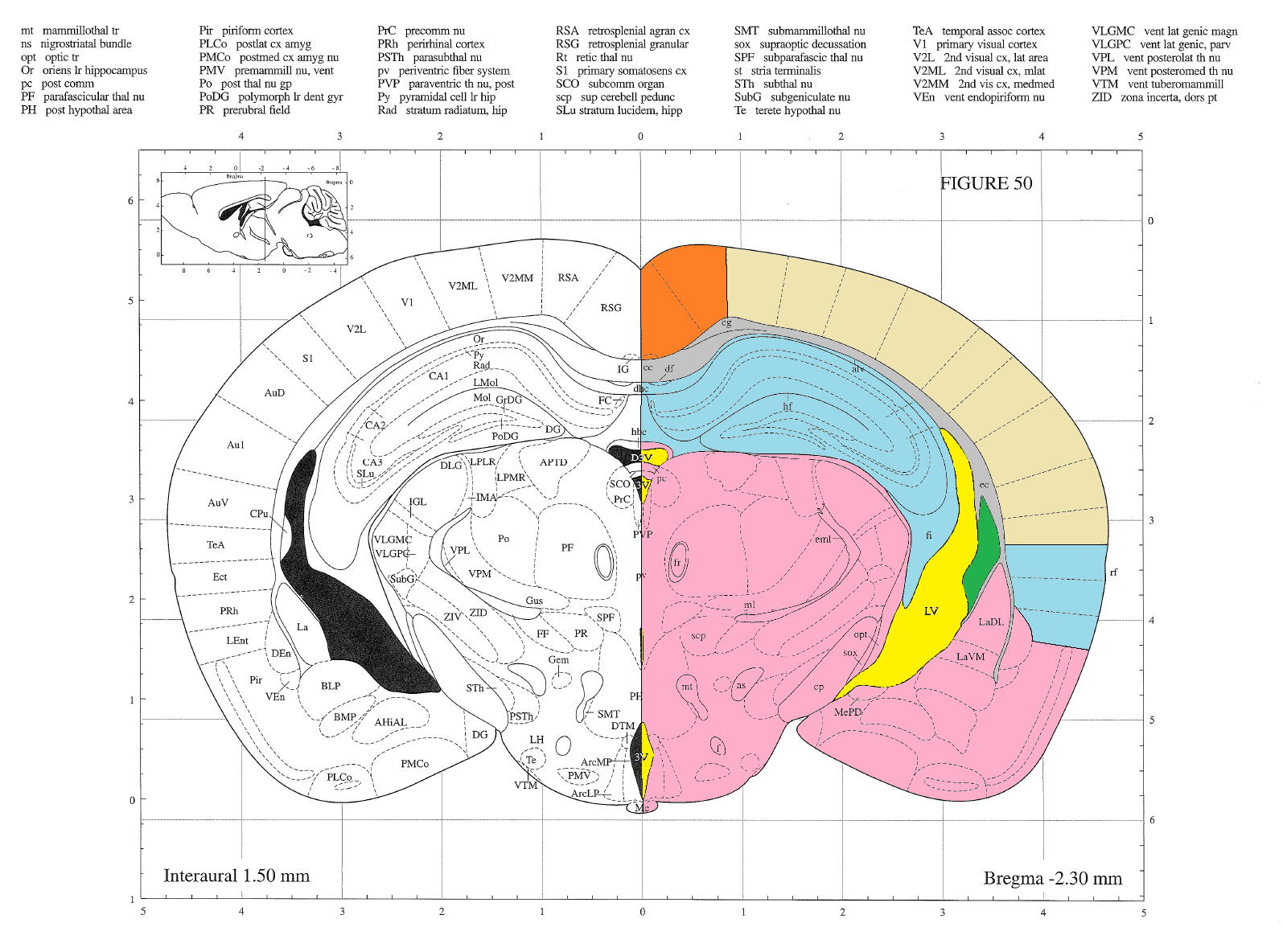

Supplement: Supplementary file 1 [file Data_Sheet_1.ZIP › Fig. S12.png]

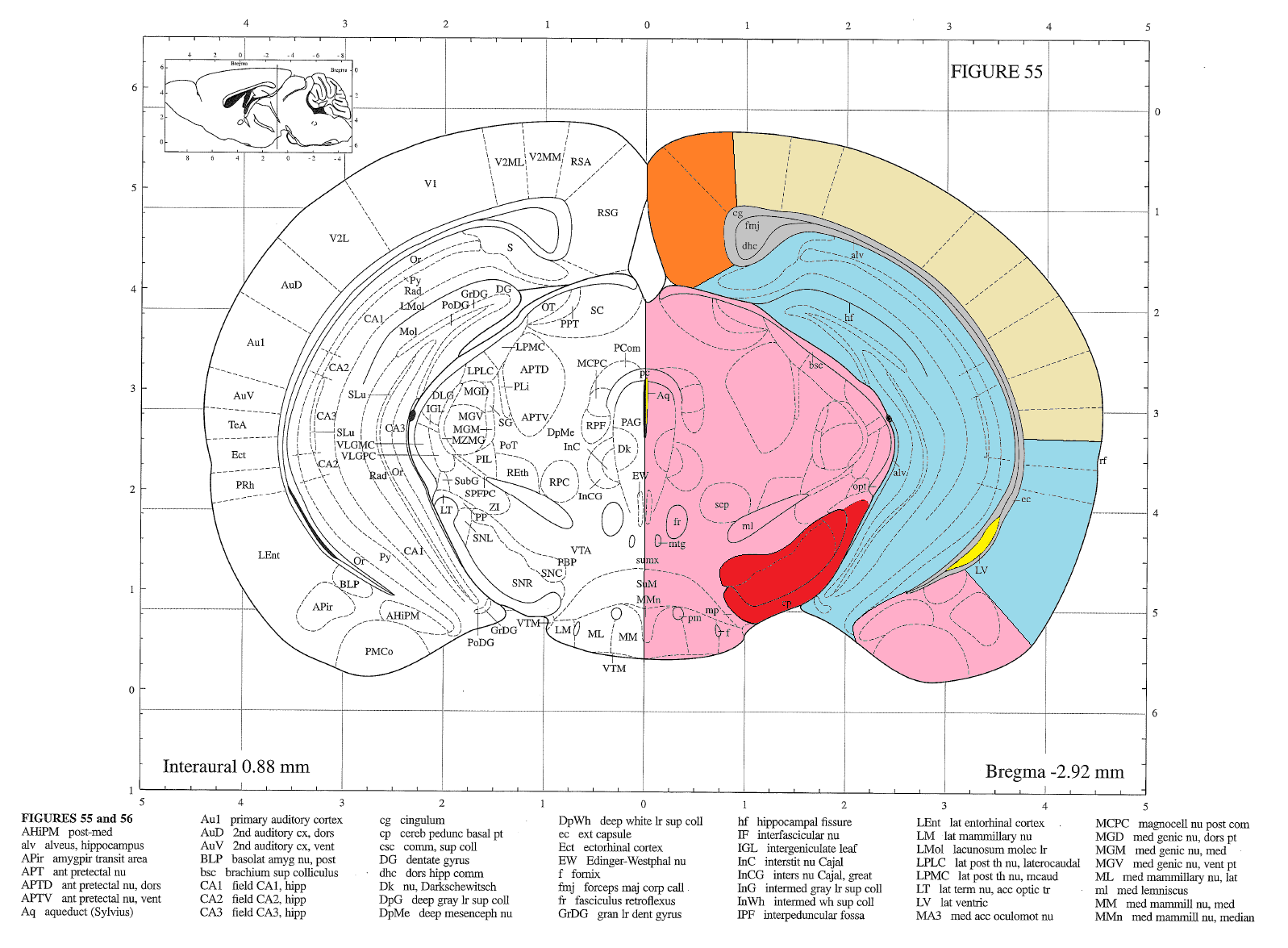

Supplement: Supplementary file 1 [file Data_Sheet_1.ZIP › Fig. S13.png]

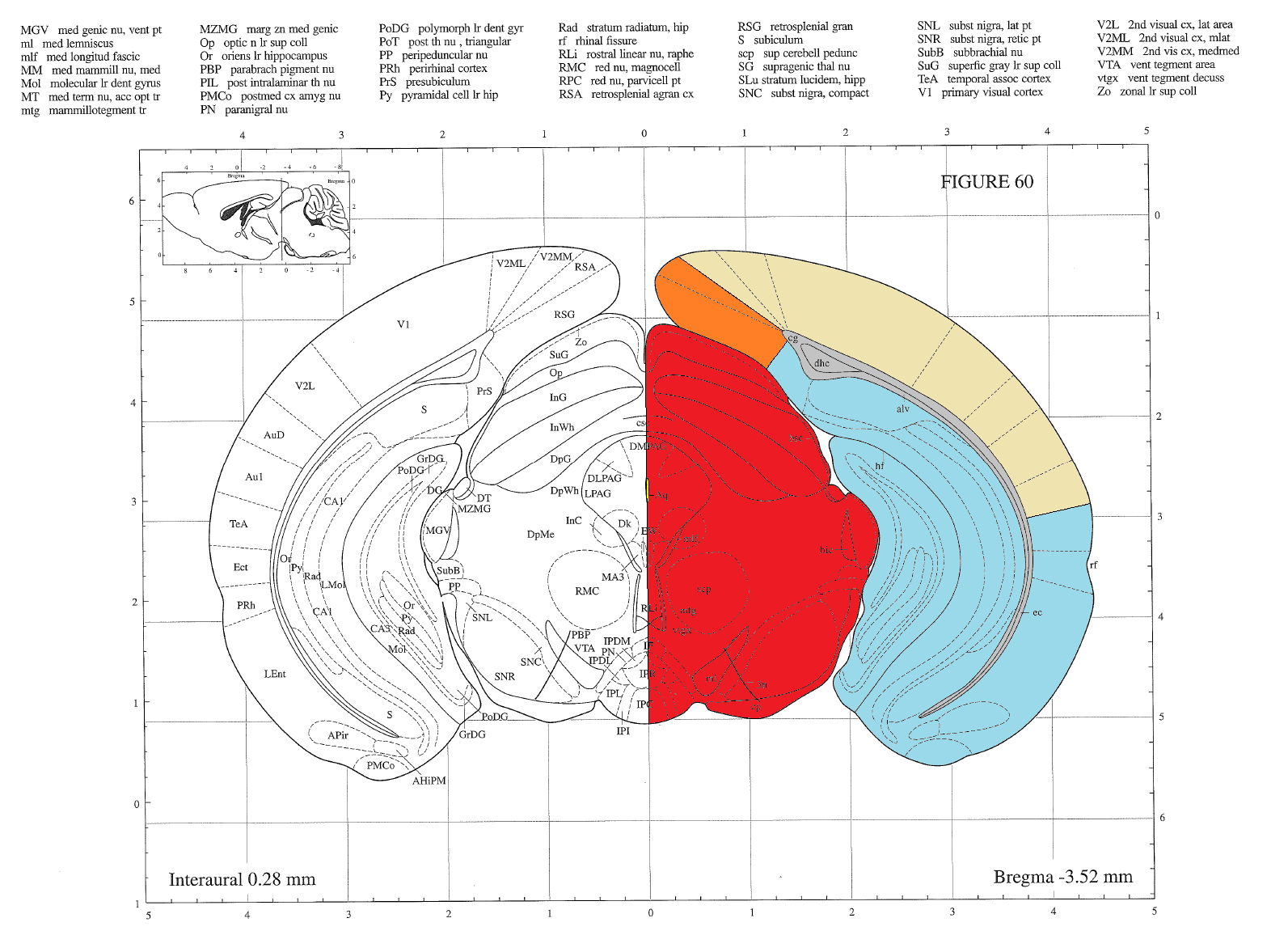

Supplement: Supplementary file 1 [file Data_Sheet_1.ZIP › Fig. S14.png]

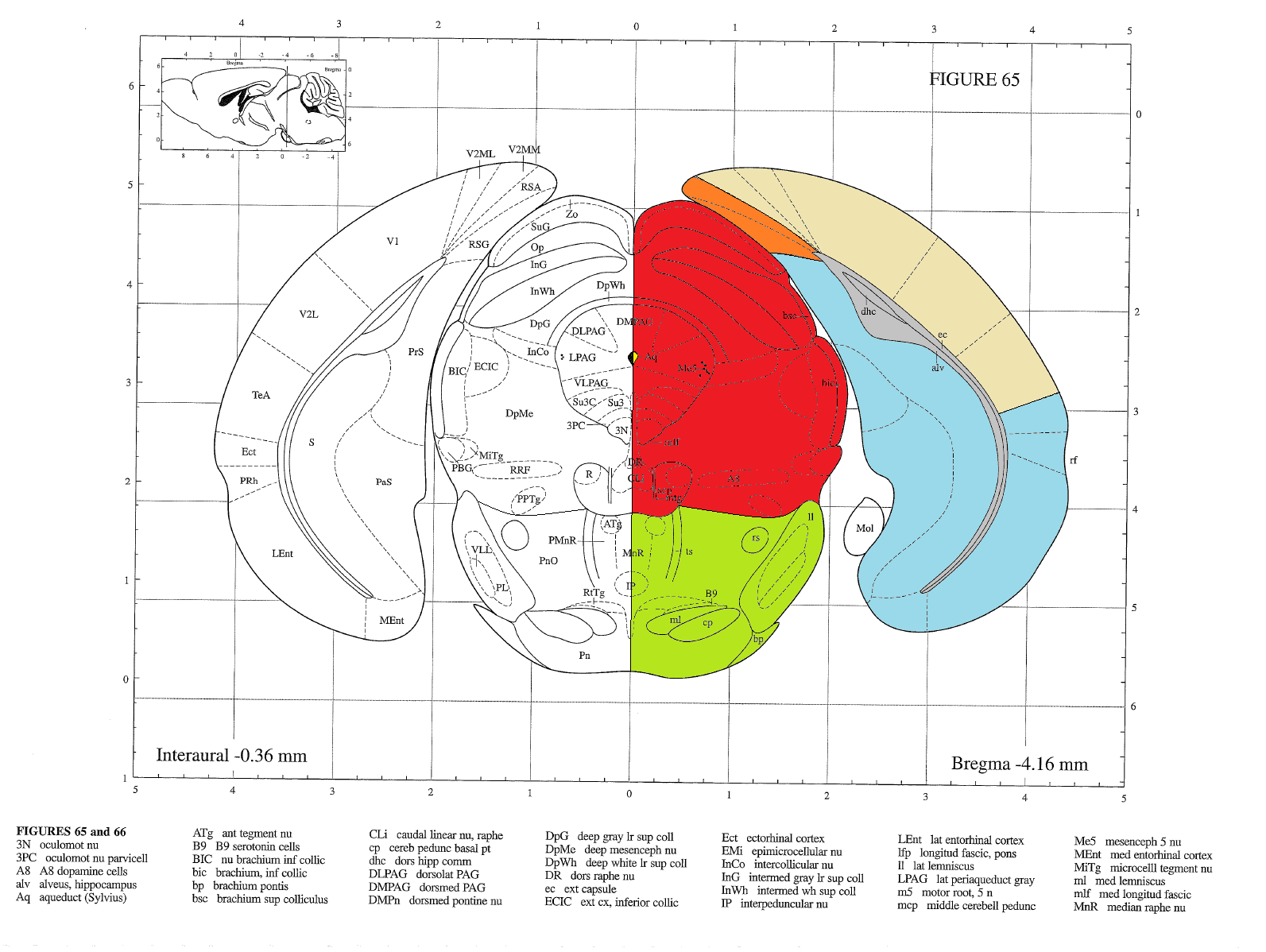

Supplement: Supplementary file 1 [file Data_Sheet_1.ZIP › Fig. S15.png]

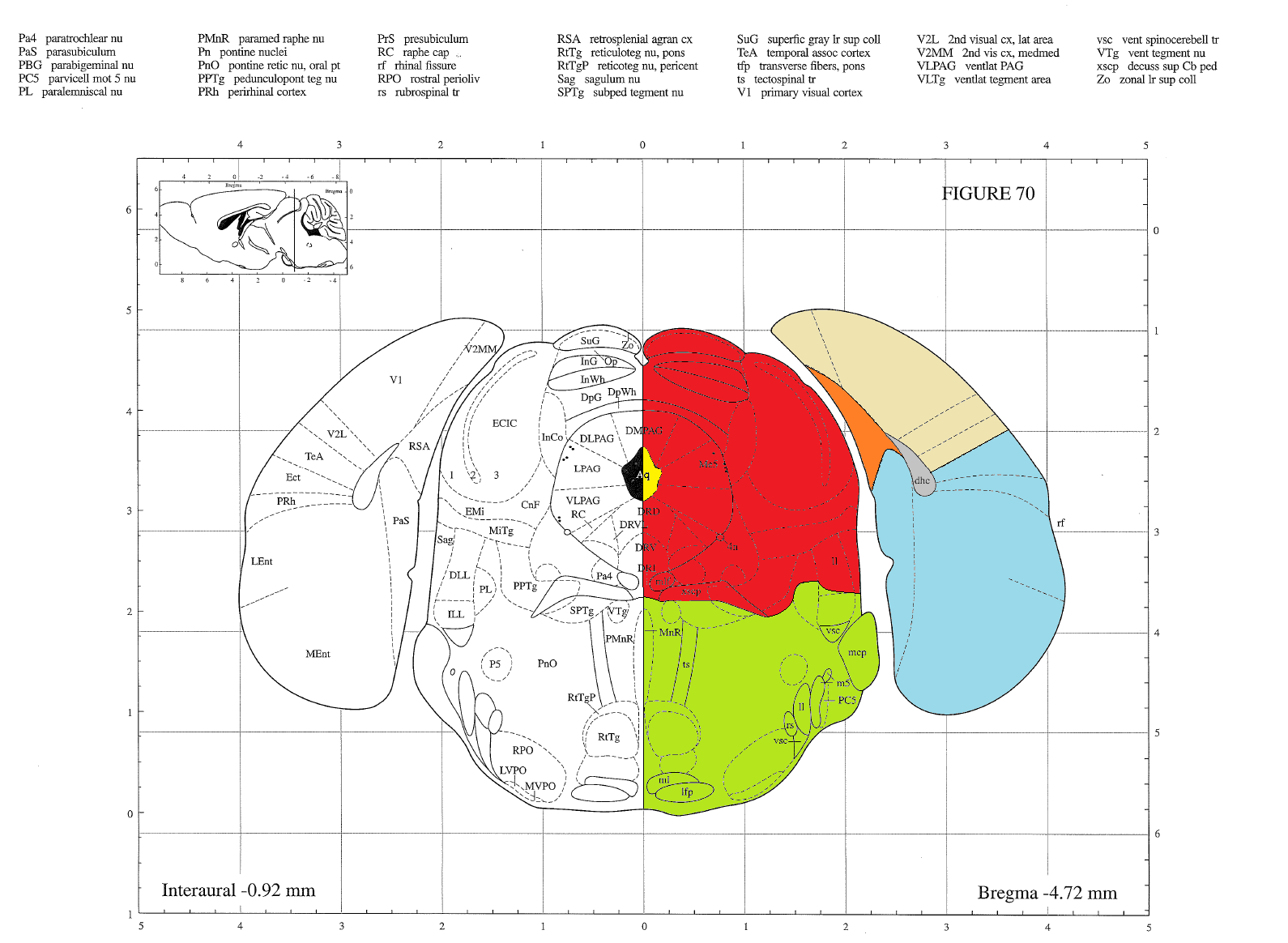

Supplement: Supplementary file 1 [file Data_Sheet_1.ZIP › Fig. S16.png]

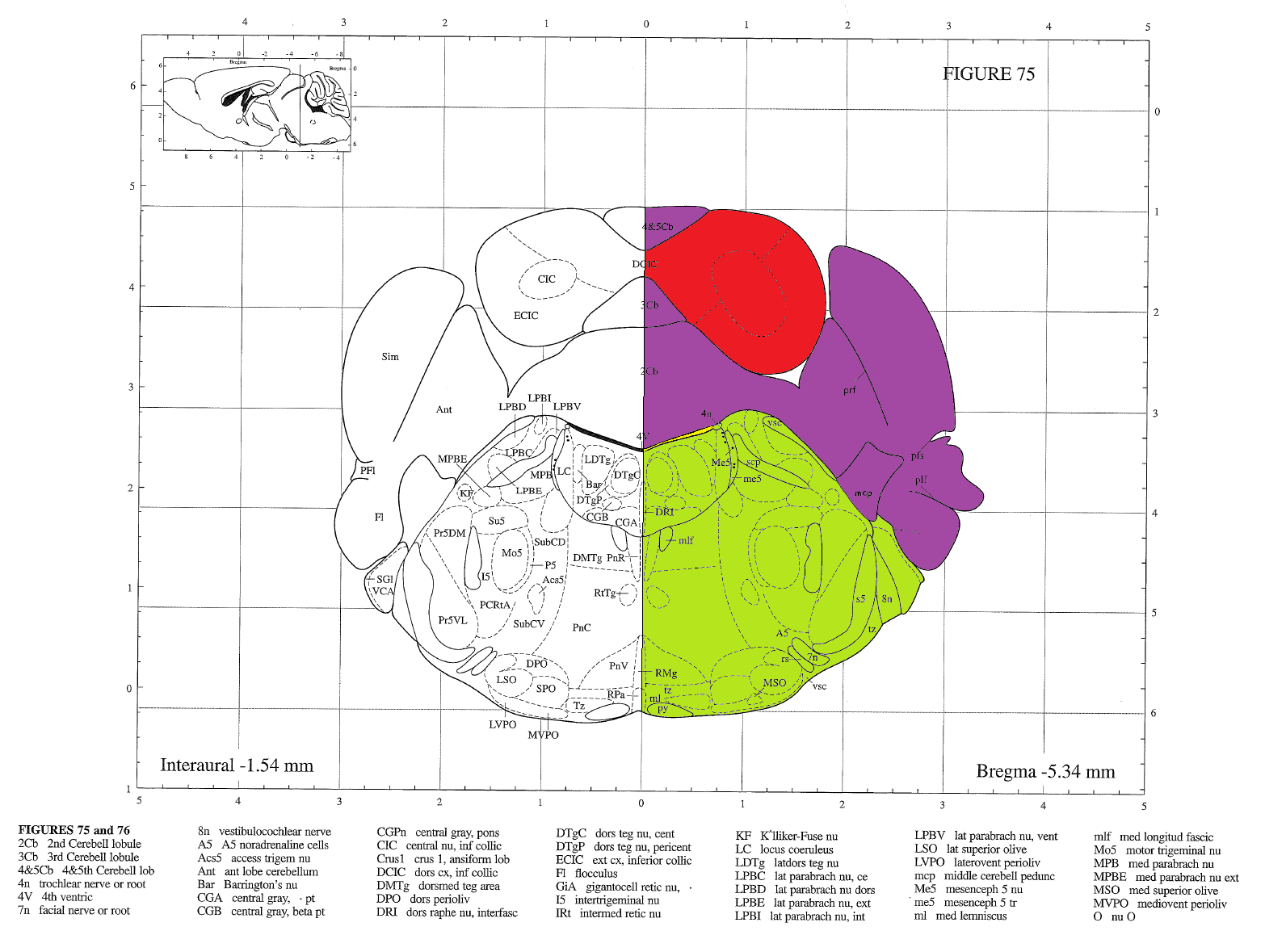

Supplement: Supplementary file 1 [file Data_Sheet_1.ZIP › Fig. S17.png]

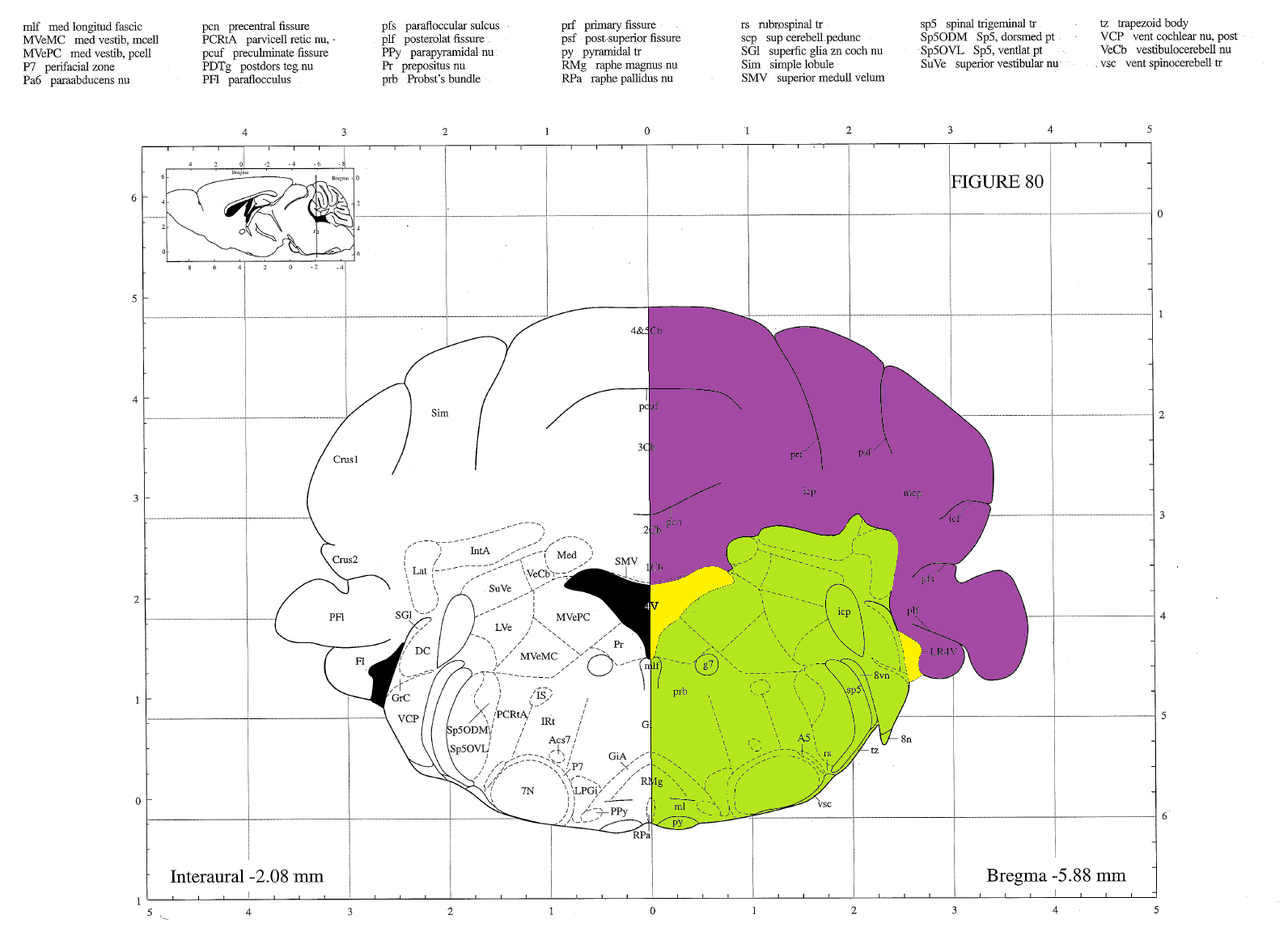

Supplement: Supplementary file 1 [file Data_Sheet_1.ZIP › Fig. S18.png]

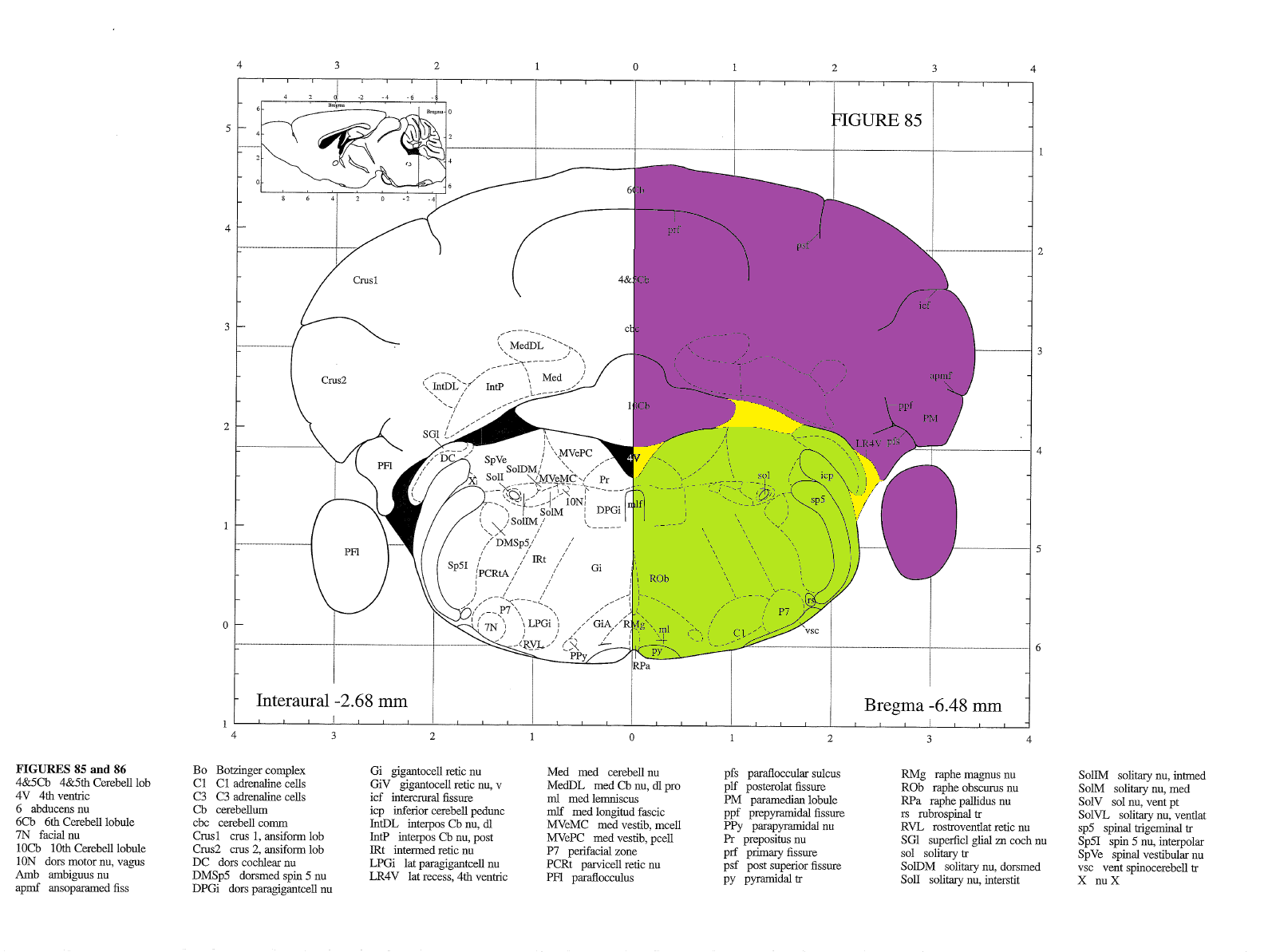

Supplement: Supplementary file 1 [file Data_Sheet_1.ZIP › Fig. S19.png]

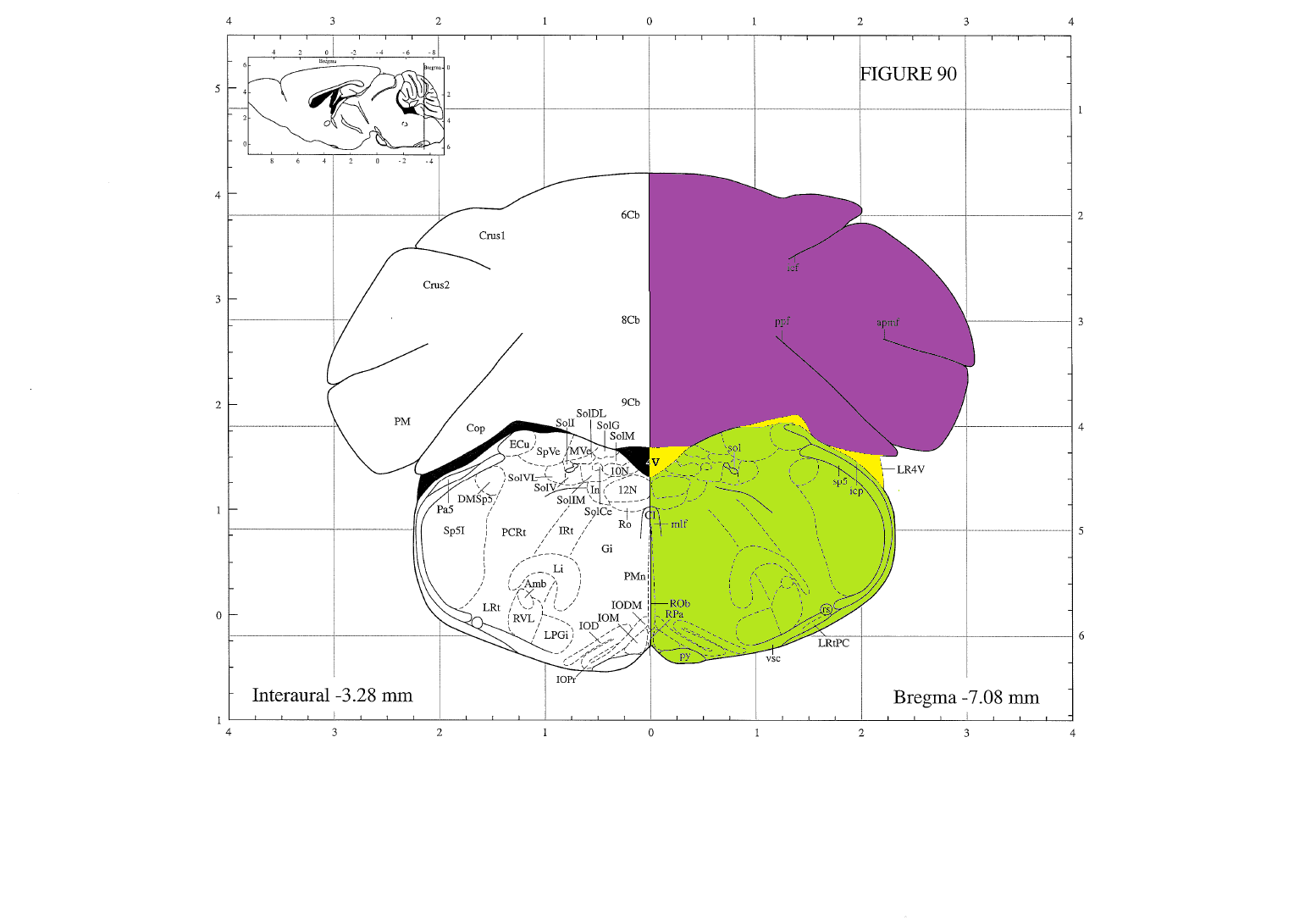

Supplement: Supplementary file 1 [file Data_Sheet_1.ZIP › Fig. S20.png]

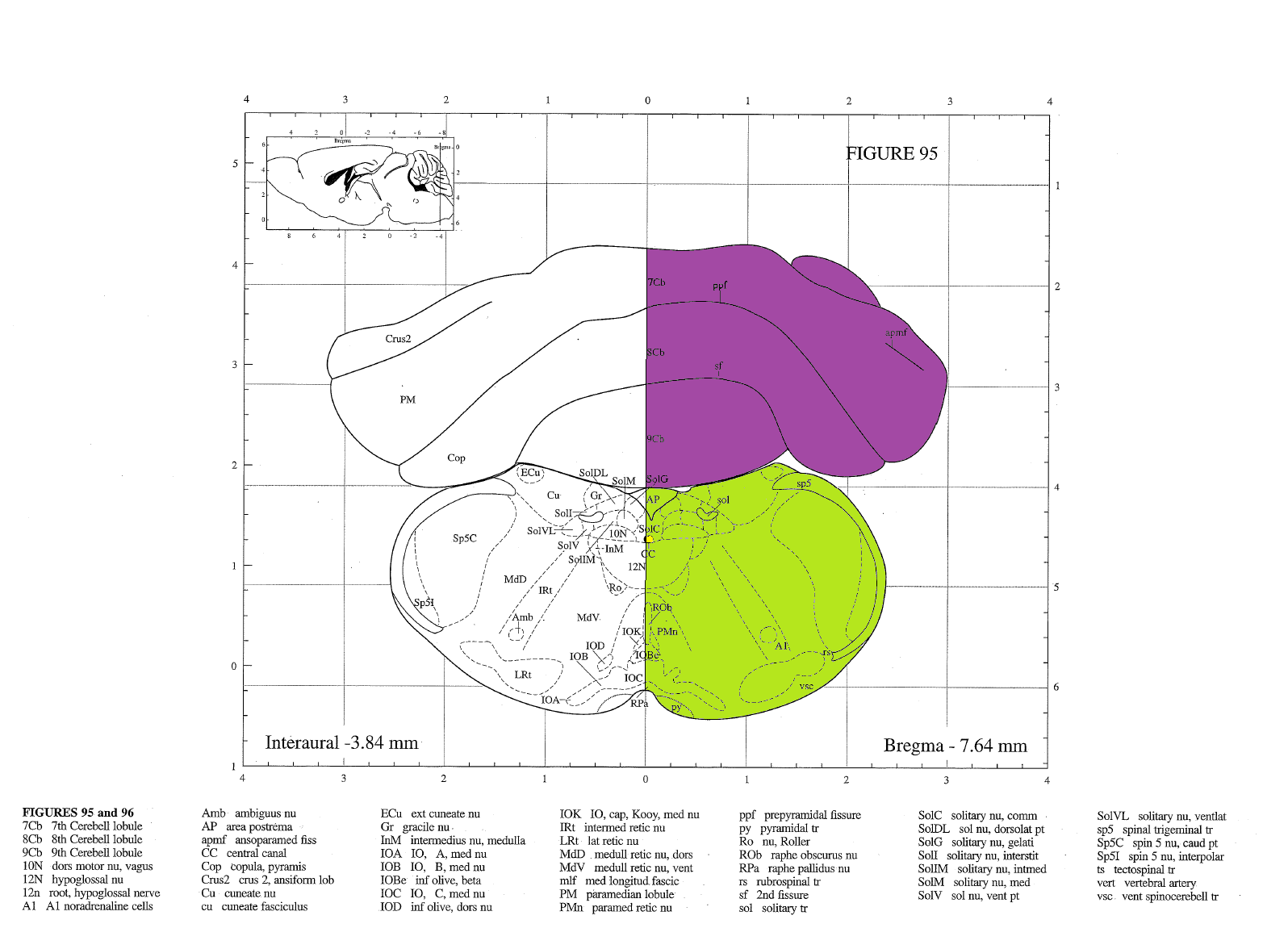

Supplement: Supplementary file 1 [file Data_Sheet_1.ZIP › Fig. S21.png]
